# Supplementary material for: Neural Network Potential with Multiresolution Approach Enables Accurate Prediction of Reaction Free Energies in Solution
Source: J Am Chem Soc. 2025 Feb 17;147(8):6835–56. doi: 10.1021/jacs.4c17015 (PMC11869291; doi:10.1021/jacs.4c17015)
Supplement: Supplementary file 1 — ja4c17015_si_001.pdf [file ja4c17015_si_001.pdf]

SUPPORTING INFORMATION
Neural Network Potential with Multiresolution Approach Enables Accurate Prediction of Reaction Free Energies in Solution

Felix Pultar,[a,†] Moritz Thürlemann,[a,†] Igor Gordiy,[a] Eva Doloszeski,[a] and Sereina Riniker[a*]

[a] *Department of Chemistry and Applied Biosciences, ETH Zürich, Vladimir-Prelog-Weg 2, 8093 Zürich, Switzerland. E-mail: sriniker@ethz.ch*

† *These authors contributed equally.*

Contents

S1 Statistical Descriptors and Correlation Plots **S2**
- S1.1 Alanine Dipeptide – AMP Model (600'000 Parameters) S2
- S1.2 Alanine Dipeptide – AMP Model (2.7 Million Parameters) S13
- S1.3 Alanine Dipeptide – GFN2-xTB S24
- S1.4 Nickel Phosphine Complexes – AMP Model (600'000 Parameters) S26
- S1.5 Nickel Phosphine Complexes – AMP Model (2.7 Million Parameters) S36
- S1.6 Nickel Phosphine Complexes – GFN2-xTB S46
- S1.7 Pyridine and Quinoline Dimers – AMP Model (600'000 Parameters) S48
- S1.8 Pyridine and Quinoline Dimers – AMP Model (2.7 Million Parameters) S58
- S1.9 Pyridine and Quinoline Dimers – GFN2-xTB S68

S2 Umbrella Definitions **S70**
- S2.1 Alanine Dipeptide S70
- S2.2 Nickel Phosphine Complexes S73
- S2.3 Pyridine and Quinoline Dimers S74

S3 Free-Energy Calculations **S76**
- S3.1 Alanine Dipeptide – AMP Model (600'000 Parameters) S76
- S3.2 Alanine Dipeptide – AMP Model (2.7 Million Parameters) S79
- S3.3 Alanine Dipeptide – GFN2-xTB S82
- S3.4 Nickel Phosphine Complexes S83
- S3.5 Pyridine and Quinoline Dimers S86

S1 Statistical Descriptors and Correlation Plots

S1.1 Alanine Dipeptide – AMP Model (600'000 Parameters)

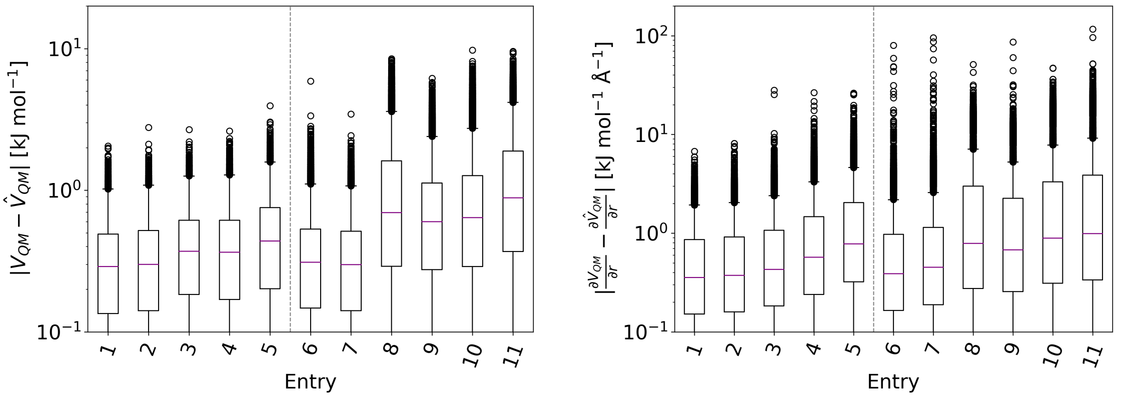

Figure S1: Distribution of the absolute errors of predicted QM energies and gradients of alanine dipeptide with respect to the reference for models trained on different training sets (see Table 1 in the main text for split definitions). All predictions were performed on the respective test sets.

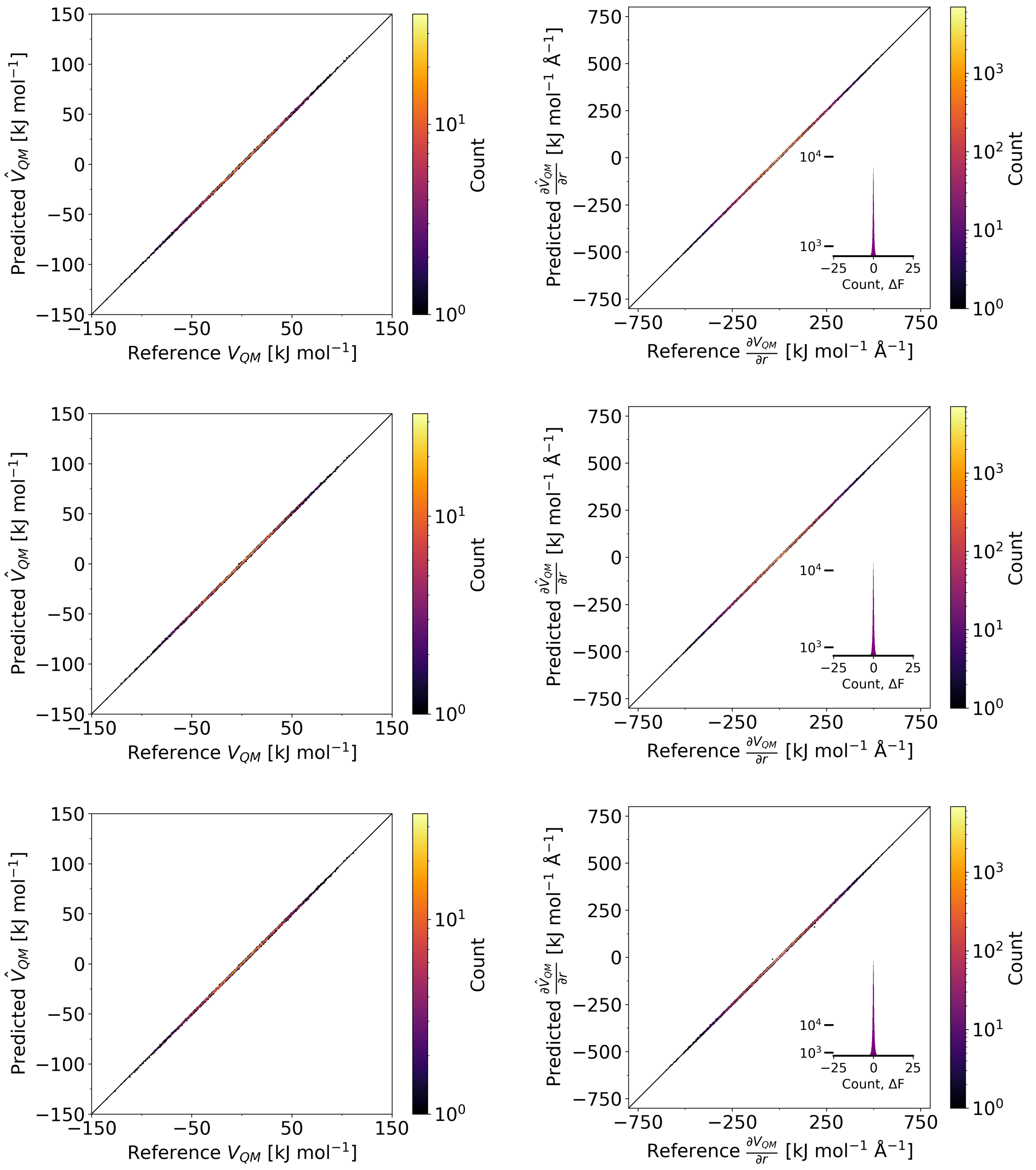

Figure S2: Correlation of predicted QM energies and gradients and their respective references using AMP. Top to bottom: entries 1–5 from Table 1 (see main text).

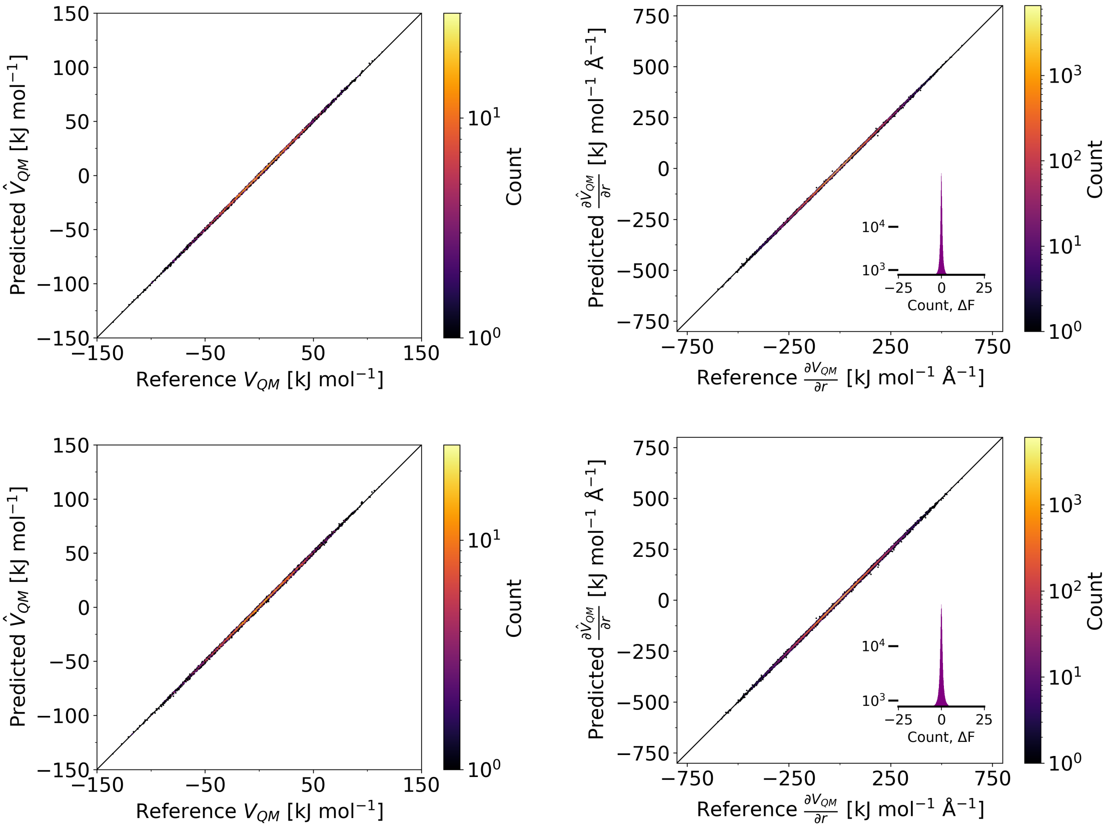

Figure S2: (cont.) Correlation of predicted QM energies and gradients and their respective references using AMP. Top to bottom: entries 1–5 from Table 1 (see main text).

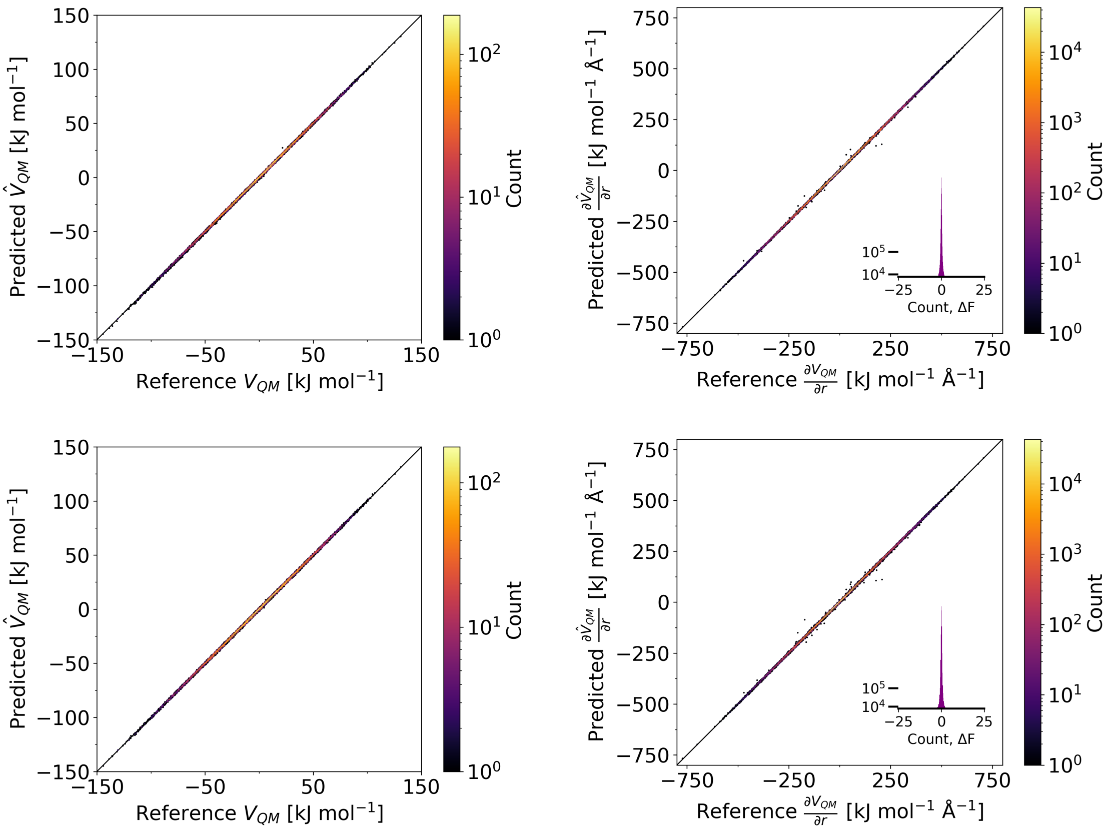

Figure S3: Correlation of predicted QM energies and gradients and their respective references using AMP. Top to bottom: entries 6–11 from Table 1 (see main text).

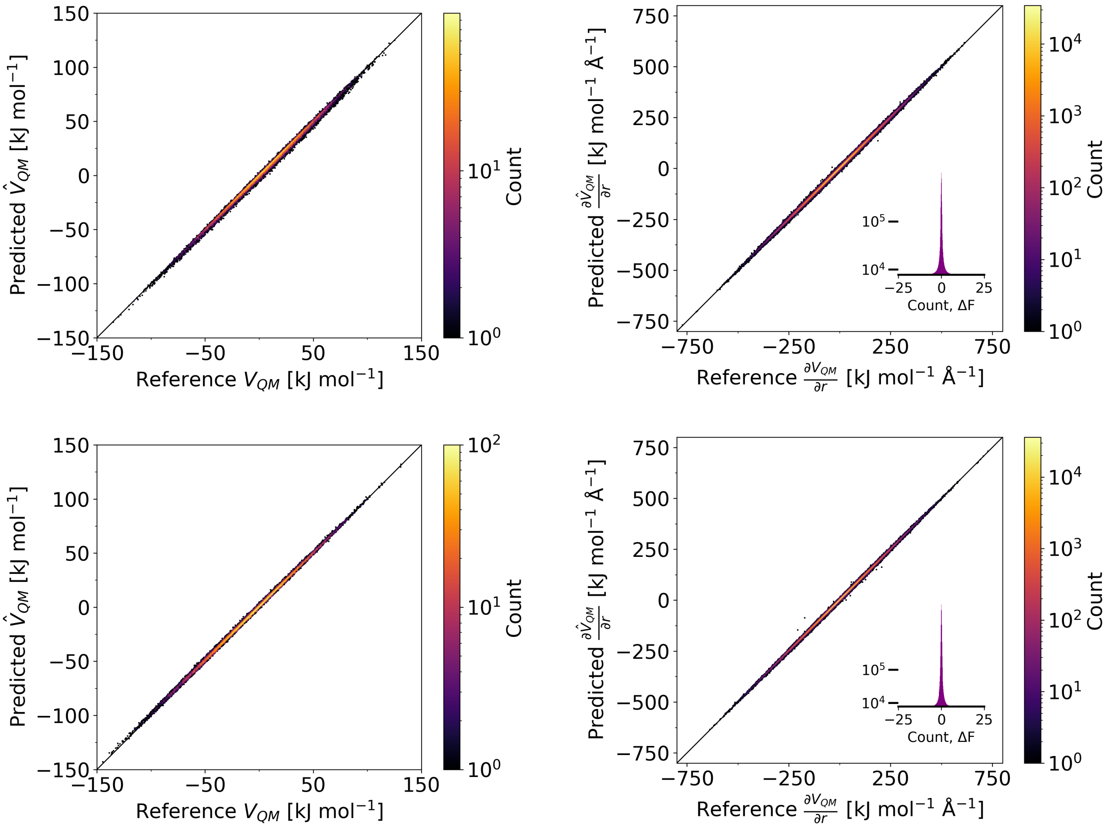

Figure S3: (cont.) Correlation of predicted QM energies and gradients and their respective references using AMP. Top to bottom: entries 6–11 from Table 1 (see main text).

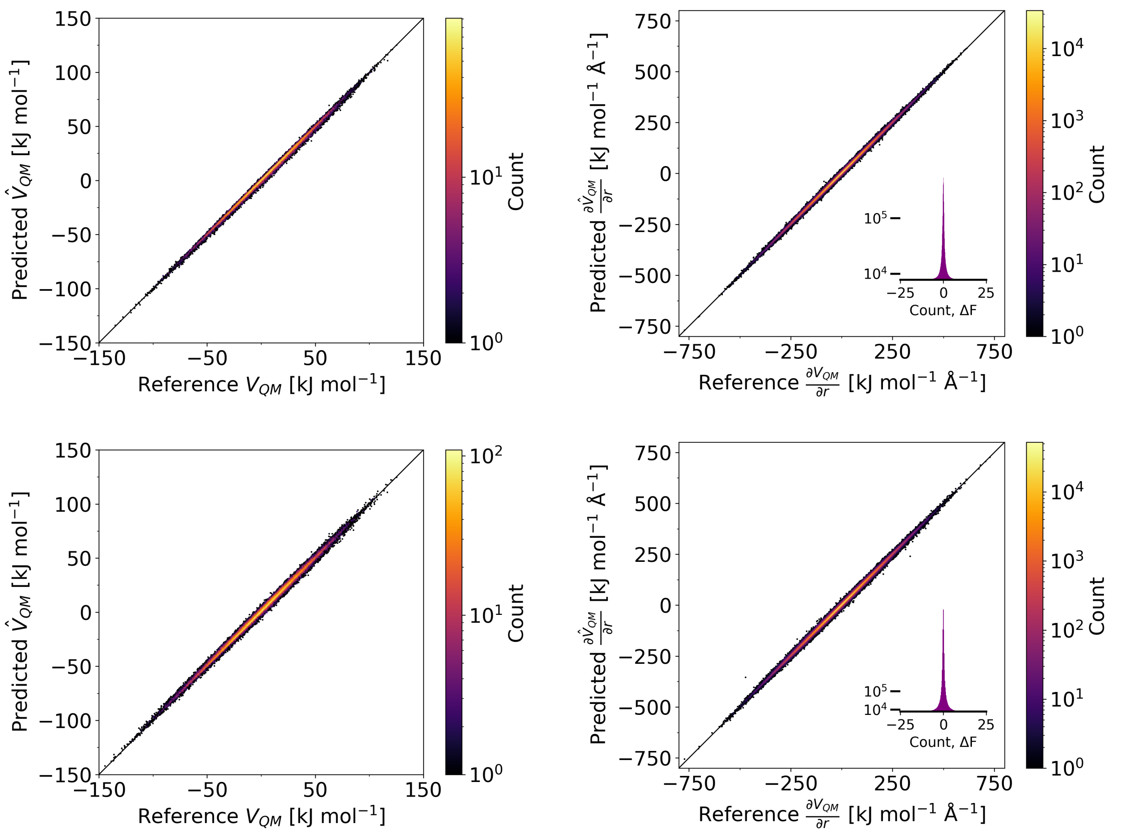

Figure S3: (cont.) Correlation of predicted QM energies and gradients and their respective references using AMP. Top to bottom: entries 6–11 from Table 1 (see main text).

Table S1: Statistical descriptors for QM energy predictions on the respective test set by models trained according to entries 1–5 from Table 1 (see main text). Values related to energy are reported in kJ mol^{-1}. Percentage Chemical Accuracy and Twice Percentage Chemical Accuracy refer to percentage of predictions within 4.184 and 8.368 kJ mol^{-1}, respectively.

Entry	1	2	3	4	5
Mean Absolute Error	0.345	0.360	0.429	0.433	0.530
Median Absolute Error	0.289	0.300	0.371	0.365	0.438
Quantile 25	0.135	0.141	0.184	0.169	0.202
Quantile 75	0.490	0.519	0.615	0.614	0.754
RMSD	0.437	0.457	0.530	0.549	0.677
Max Unsigned Error	2.05	2.78	2.68	2.62	3.95
Max Signed Error	1.43	2.12	2.68	2.17	3.95
Min Signed Error	-2.05	-2.78	-1.70	-2.62	-3.03
Spearman ρ	1.00	1.00	1.00	1.00	1.00
Kendall τ	0.992	0.992	0.992	0.989	0.987
Spearman P-Value	0	0	0	0	0
Kendall τ P-Value	0	0	0	0	0
Perc. Chem. Acc.	1.00	1.00	1.00	1.00	1.00
Twice Perc. Chem. Acc.	1.00	1.00	1.00	1.00	1.00

Table S2: Statistical descriptors for QM energy predictions on the respective test set by models trained according to entries 6–11 from Table 1 (see main text). Values related to energy are reported in kJ mol^{-1}. Percentage Chemical Accuracy and Twice Percentage Chemical Accuracy refer to percentage of predictions within 4.184 and 8.368 kJ mol^{-1}, respectively.

Entry	6	7	8	9	10	11
Mean Absolute Error	0.374	0.360	1.11	0.801	0.948	1.27
Median Absolute Error	0.310	0.298	0.695	0.600	0.641	0.885
Quantile 25	0.147	0.141	0.291	0.275	0.289	0.369
Quantile 75	0.531	0.515	1.61	1.13	1.27	1.89
RMSD	0.478	0.458	1.57	1.07	1.34	1.73
Max Unsigned Error	5.89	3.45	8.48	6.17	9.75	9.57
Max Signed Error	3.36	3.45	8.48	3.22	9.75	9.50
Min Signed Error	-5.89	-2.44	-4.07	-6.17	-6.14	-9.57
Spearman ρ	1.00	1.00	0.999	1.00	0.999	0.999
Kendall τ	0.992	0.991	0.973	0.982	0.976	0.969
Spearman P-Value	0	0	0	0	0	0
Kendall τ P-Value	0	0	0	0	0	0
Perc. Chem. Acc.	1.00	1.00	0.979	0.999	0.987	0.973
Twice Perc. Chem. Acc.	1.00	1.00	1.00	1.00	1.00	1.00

Table S3: Statistical descriptors for QM gradient predictions on the respective test set by models trained according to entries 1–5 from Table 1 (see main text). Values related to gradients are reported in kJ mol^{-1} Å^{-1}.

	1	2	3	4	5
Mean Absolute Error	0.375	0.397	0.475	0.642	0.907
Median Absolute Error	0.267	0.279	0.328	0.433	0.595
Quantile 25	0.120	0.126	0.147	0.192	0.262
Quantile 75	0.507	0.536	0.635	0.856	1.20
RMSD	0.523	0.559	0.680	0.931	1.33
Max Unsigned Error	6.73	8.13	28.0	26.5	26.2
Max Signed Error	6.73	8.13	28.0	26.5	26.2
Min Signed Error	-5.93	-8.11	-25.4	-21.6	-25.3
Spearman ρ	1.00	1.00	1.00	1.00	1.00
Kendall τ	0.996	0.996	0.995	0.994	0.991
Spearman P-Value	0	0	0	0	0
Kendall τ P-Value	0	0	0	0	0

Table S4: Statistical descriptors for QM gradient predictions on the respective test set by models trained according to entries 6–11 from Table 1 (see main text). Values related to gradients are reported in kJ mol^{-1} Å^{-1}.

	6	7	8	9	10	11
Mean Absolute Error	0.412	0.485	1.12	0.880	1.27	1.46
Median Absolute Error	0.290	0.334	0.533	0.479	0.611	0.677
Quantile 25	0.131	0.150	0.214	0.202	0.247	0.268
Quantile 75	0.555	0.649	1.34	1.08	1.50	1.73
RMSD	0.586	0.701	1.94	1.46	2.22	2.58
Max Unsigned Error	79.7	95.5	51.3	86.3	47.1	117
Max Signed Error	79.7	95.5	51.3	44.3	47.1	95.9
Min Signed Error	-48.8	-86.8	-42.7	-86.3	-46.8	-117
Spearman ρ	1.00	1.00	1.00	1.00	1.00	0.999
Kendall τ	0.996	0.995	0.988	0.991	0.986	0.984
Spearman P-Value	0	0	0	0	0	0
Kendall τ P-Value	0	0	0	0	0	0

Table S5: Statistical descriptors for MM gradient predictions on the respective test set by models trained according to entries 1–5 from Table 1 (see main text). Values related to gradients are reported in kJ mol^{-1} Å^{-1}.

	1	2	3	4	5
Mean Absolute Error	0.0203	0.0196	0.0201	0.0219	0.0245
Median Absolute Error	0.0112	0.0109	0.0111	0.0115	0.0123
Quantile 25	0.00476	0.00466	0.00473	0.00489	0.00520
Quantile 75	0.0242	0.0233	0.0238	0.0250	0.0272
RMSD	0.0354	0.0351	0.0360	0.0425	0.0496
Max Unsigned Error	5.60	5.65	6.64	11.1	10.0
Max Signed Error	5.43	4.79	6.54	11.1	10.0
Min Signed Error	-5.60	-5.65	-6.64	-8.08	-9.40
Spearman ρ	0.999	0.999	0.999	0.999	0.999
Kendall τ	0.977	0.978	0.978	0.977	0.975
Spearman P-Value	0	0	0	0	0
Kendall τ P-Value	0	0	0	0	0

Table S6: Statistical descriptors for MM gradient predictions on the respective test set by models trained according to entries 6–11 from Table 1 (see main text). Values related to gradients are reported in kJ mol^{-1} Å^{-1}.

	6	7	8	9	10	11
Mean Absolute Error	0.0194	0.0199	0.0228	0.0210	0.0229	0.0243
Median Absolute Error	0.0108	0.0110	0.0117	0.0111	0.0115	0.0119
Quantile 25	0.00463	0.00469	0.00493	0.00474	0.00490	0.00504
Quantile 75	0.0230	0.0235	0.0257	0.0239	0.0252	0.0259
RMSD	0.0356	0.0363	0.0441	0.0409	0.0469	0.0531
Max Unsigned Error	23.9	11.0	7.17	12.3	7.74	18.0
Max Signed Error	23.9	11.0	6.35	10.4	7.74	18.0
Min Signed Error	-6.64	-5.66	-7.17	-12.3	-5.49	-17.8
Spearman ρ	0.999	0.999	0.999	0.999	0.999	0.999
Kendall τ	0.979	0.978	0.976	0.978	0.977	0.978
Spearman P-Value	0	0	0	0	0	0
Kendall τ P-Value	0	0	0	0	0	0

Table S7: Statistical descriptors for QM dipole predictions on the respective test set by models trained according to entries 1–5 from Table 1 (see main text). Values related to QM dipoles are reported in eÅ.

	1	2	3	4	5
Mean Absolute Error	0.0308	0.0305	0.0307	0.0316	0.0331
Median Absolute Error	0.0259	0.0256	0.0257	0.0265	0.0280
Quantile 25	0.0123	0.0123	0.0122	0.0126	0.0132
Quantile 75	0.0444	0.0437	0.0444	0.0454	0.0474
RMSD	0.0388	0.0383	0.0387	0.0398	0.0416
Max Unsigned Error	0.174	0.172	0.176	0.174	0.189
Max Signed Error	0.174	0.172	0.176	0.174	0.189
Min Signed Error	-0.164	-0.162	-0.162	-0.172	-0.171
Spearman ρ	0.999	0.999	0.999	0.999	0.999
Kendall τ	0.971	0.971	0.971	0.970	0.969
Spearman P-Value	0	0	0	0	0
Kendall τ P-Value	0	0	0	0	0

Table S8: Statistical descriptors for QM dipole predictions on the respective test set by models trained according to entries 6–11 from Table 1 (see main text). Values related to QM dipoles are reported in eÅ.

	6	7	8	9	10	11
Mean Absolute Error	0.0304	0.0308	0.0317	0.0310	0.0315	0.0325
Median Absolute Error	0.0256	0.0259	0.0266	0.0260	0.0265	0.0273
Quantile 25	0.012	0.0122	0.0125	0.0124	0.0125	0.0129
Quantile 75	0.0438	0.0442	0.0455	0.0445	0.0454	0.0468
RMSD	0.0382	0.0387	0.0398	0.0389	0.0397	0.0409
Max Unsigned Error	0.177	0.181	0.186	0.187	0.175	0.200
Max Signed Error	0.175	0.181	0.186	0.187	0.175	0.200
Min Signed Error	-0.177	-0.177	-0.171	-0.171	-0.173	-0.19
Spearman ρ	0.999	0.999	0.999	0.999	0.999	0.999
Kendall τ	0.971	0.971	0.970	0.971	0.970	0.971
Spearman P-Value	0	0	0	0	0	0
Kendall τ P-Value	0	0	0	0	0	0

Table S9: Statistical descriptors for QM quadrupole predictions on the respective test set by models trained according to entries 1–5 from Table 1 (see main text). Values related to QM quadrupoles are reported in eÅ^2.

	1	2	3	4	5
Mean Absolute Error	0.0262	0.0263	0.0273	0.0309	0.0354
Median Absolute Error	0.0217	0.0220	0.0227	0.0257	0.0294
Quantile 25	0.0102	0.0103	0.0107	0.0121	0.0138
Quantile 75	0.0375	0.0377	0.0392	0.0443	0.0505
RMSD	0.0332	0.0333	0.0346	0.0391	0.0450
Max Unsigned Error	0.166	0.151	0.156	0.199	0.227
Max Signed Error	0.149	0.143	0.156	0.199	0.227
Min Signed Error	-0.166	-0.151	-0.144	-0.176	-0.208
Spearman ρ	1.00	1.00	1.00	1.00	0.999
Kendall τ	0.985	0.985	0.984	0.982	0.979
Spearman P-Value	0	0	0	0	0
Kendall τ P-Value	0	0	0	0	0

Table S10: Statistical descriptors for QM quadrupole predictions on the respective test set by models trained according to entries 6–11 from Table 1 (see main text). Values related to QM quadrupoles are reported in eÅ^2.

	6	7	8	9	10	11
Mean Absolute Error	0.0265	0.0277	0.0464	0.0450	0.0525	0.0508
Median Absolute Error	0.0220	0.0231	0.0366	0.0346	0.0409	0.0398
Quantile 25	0.0104	0.0108	0.0169	0.016	0.0188	0.0183
Quantile 75	0.0379	0.0396	0.0657	0.0623	0.0744	0.0721
RMSD	0.0336	0.035	0.0602	0.0599	0.0687	0.0662
Max Unsigned Error	0.197	0.184	0.335	0.385	0.364	0.356
Max Signed Error	0.164	0.184	0.332	0.364	0.341	0.356
Min Signed Error	-0.197	-0.179	-0.335	-0.385	-0.364	-0.356
Spearman ρ	1.00	1.00	0.999	0.999	0.999	0.999
Kendall τ	0.984	0.984	0.973	0.972	0.970	0.968
Spearman P-Value	0	0	0	0	0	0
Kendall τ P-Value	0	0	0	0	0	0

S1.2 Alanine Dipeptide – AMP Model (2.7 Million Parameters)

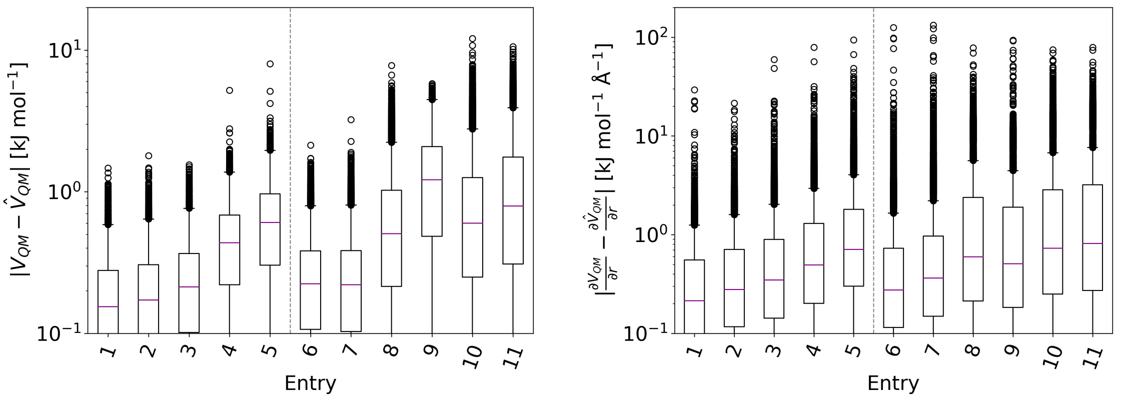

Figure S4: Distribution of the absolute errors of predicted QM energies and gradients of alanine dipeptide with respect to the reference for models trained on different training sets (see Table 1 in the main text for split definitions). All predictions were performed on the respective test sets.

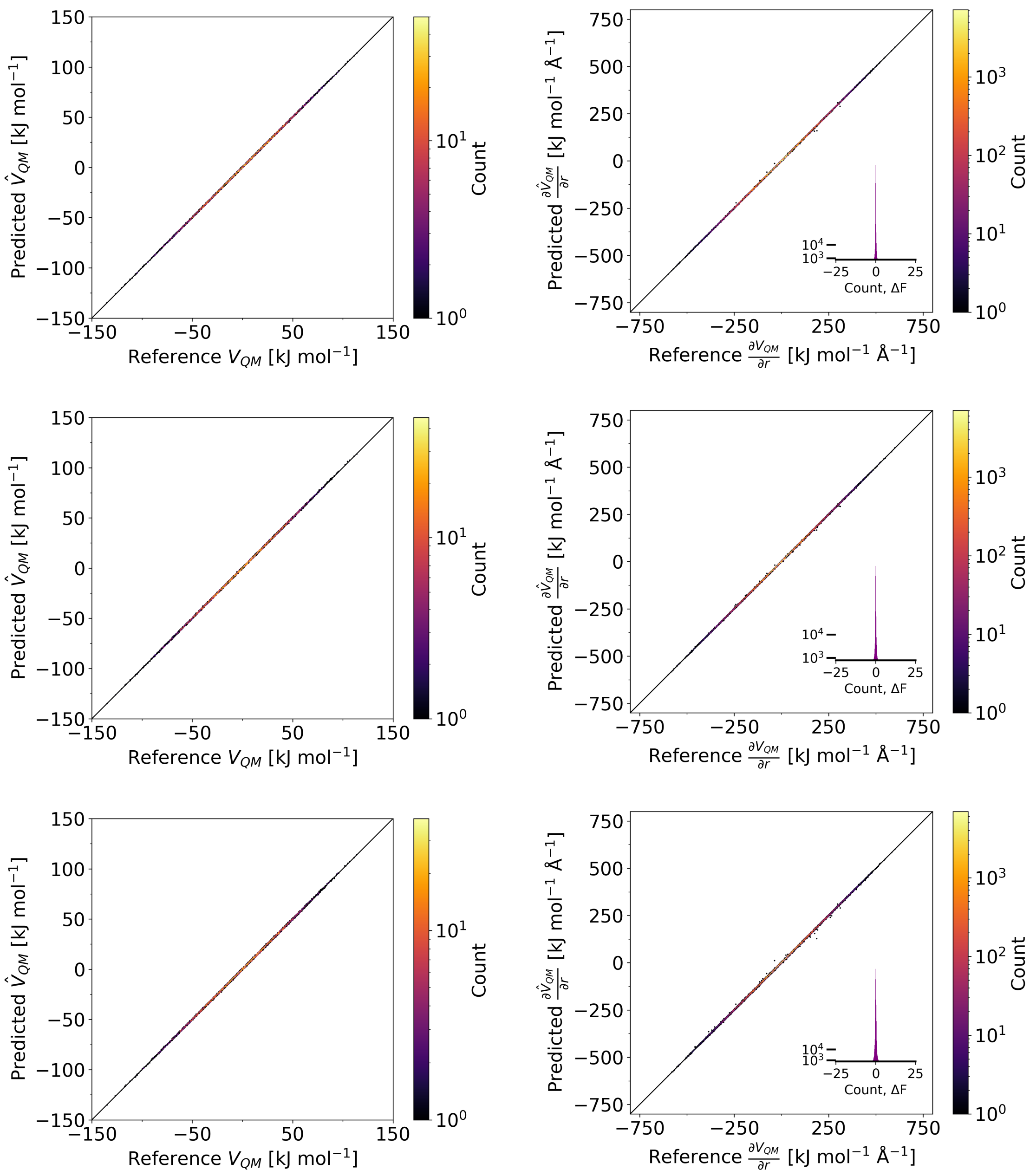

Figure S5: Correlation of predicted QM energies and gradients and their respective references using AMP. Top to bottom: entries 1–5 from Table 1 (see main text).

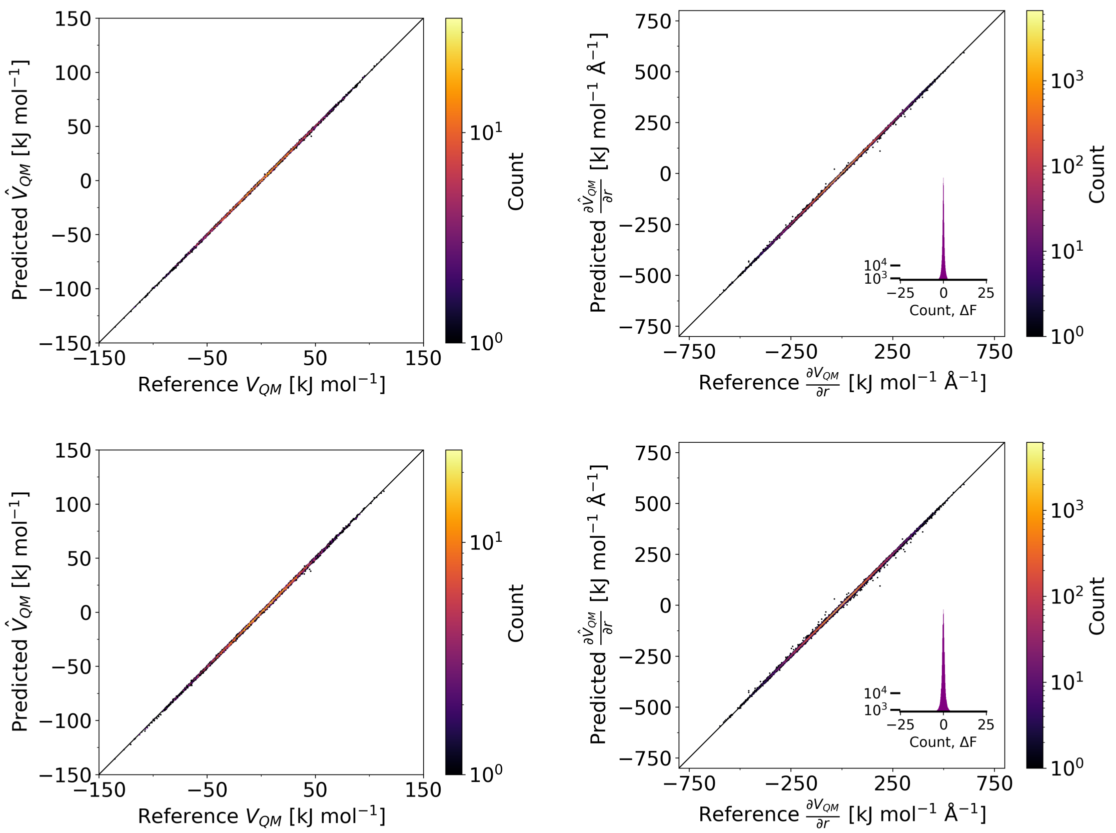

Figure S5: (cont.) Correlation of predicted QM energies and gradients and their respective references using AMP. Top to bottom: entries 1–5 from Table 1 (see main text).

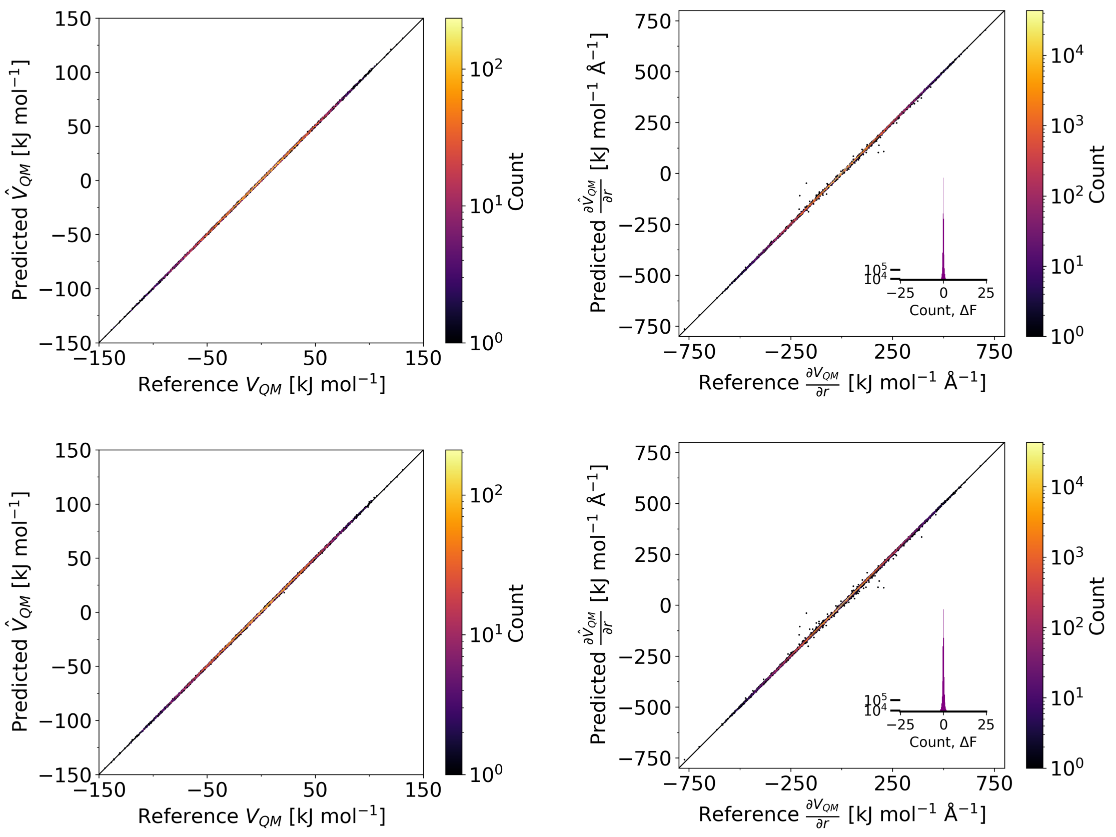

Figure S6: Correlation of predicted QM energies and gradients and their respective references using AMP. Top to bottom: entries 6–11 from Table 1 (see main text).

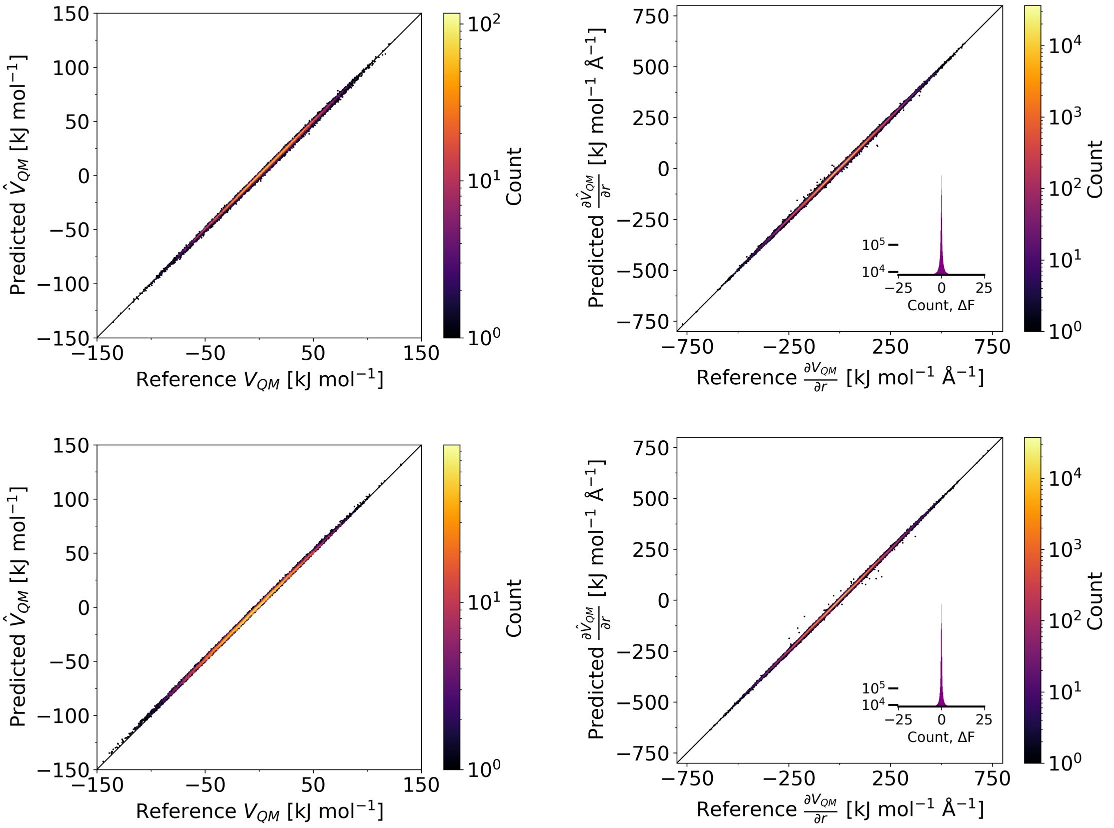

Figure S6: (cont.) Correlation of predicted QM energies and gradients and their respective references using AMP. Top to bottom: entries 6–11 from Table 1 (see main text).

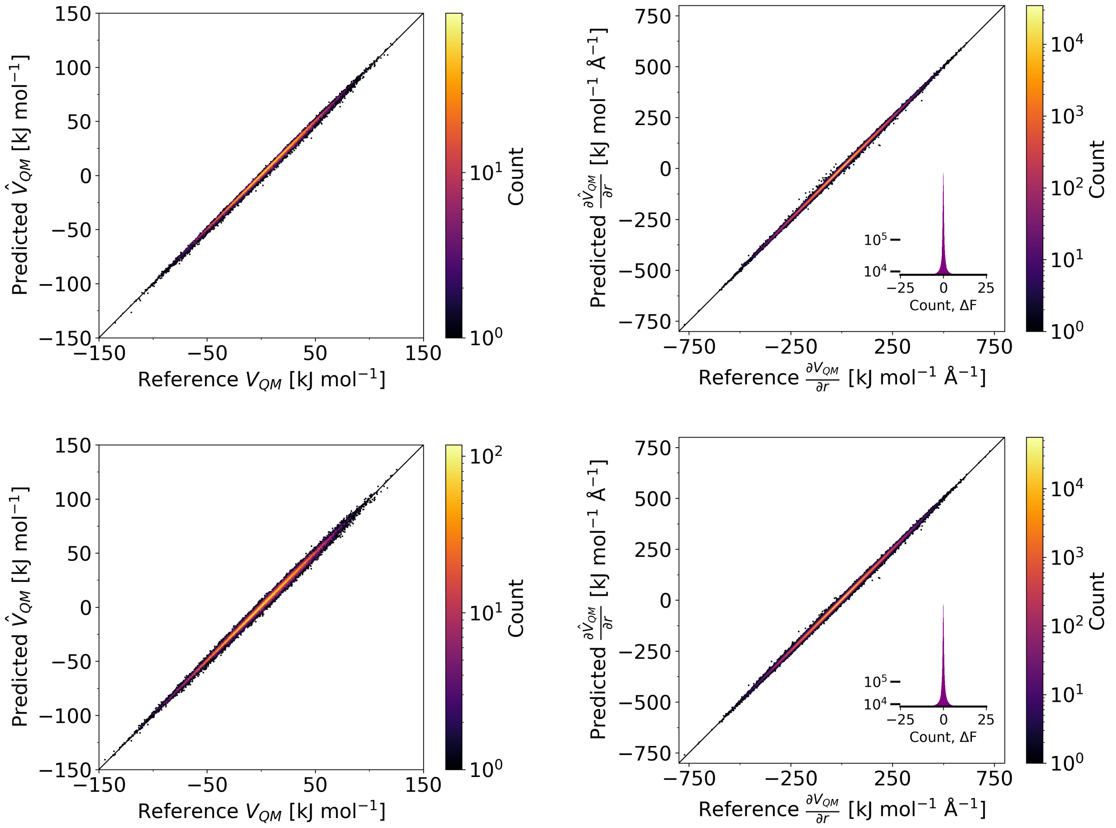

Figure S6: (cont.) Correlation of predicted QM energies and gradients and their respective references using AMP. Top to bottom: entries 6–11 from Table 1 (see main text).

Table S11: Statistical descriptors for QM energy predictions on the respective test set by models trained according to entries 1–5 from Table 1 (see main text). Values related to energy are reported in kJ mol^{-1}. Percentage Chemical Accuracy and Twice Percentage Chemical Accuracy refer to percentage of predictions within 4.184 and 8.368 kJ mol^{-1}, respectively.

	1	2	3	4	5
Mean Absolute Error	0.197	0.216	0.257	0.478	0.676
Median Absolute Error	0.155	0.172	0.213	0.436	0.606
Quantile 25	0.0733	0.0821	0.102	0.220	0.303
Quantile 75	0.279	0.306	0.367	0.683	0.967
RMSD	0.258	0.280	0.326	0.580	0.827
Max Unsigned Error	1.47	1.80	1.55	5.21	8.01
Max Signed Error	1.08	1.80	1.55	5.21	8.01
Min Signed Error	-1.47	-1.47	-1.45	-2.25	-2.51
Spearman ρ	1.00	1.00	1.00	1.00	1.00
Kendall τ	0.996	0.995	0.994	0.992	0.988
Spearman P-Value	0	0	0	0	0
Kendall τ P-Value	0	0	0	0	0
Perc. Chem. Acc.	1.00	1.00	1.00	1.00	1.00
Twice Perc. Chem. Acc.	1.00	1.00	1.00	1.00	1.00

Table S12: Statistical descriptors for QM energy predictions on the respective test set by models trained according to entries 6–11 from Table 1 (see main text). Values related to energy are reported in kJ mol^{-1}. Percentage Chemical Accuracy and Twice Percentage Chemical Accuracy refer to percentage of predictions within 4.184 and 8.368 kJ mol^{-1}, respectively.

	6	7	8	9	10	11
Mean Absolute Error	0.268	0.271	0.724	1.37	0.906	1.19
Median Absolute Error	0.224	0.220	0.506	1.21	0.599	0.792
Quantile 25	0.107	0.103	0.215	0.485	0.249	0.309
Quantile 75	0.383	0.384	1.02	2.08	1.26	1.75
RMSD	0.341	0.350	1.00	1.71	1.29	1.67
Max Unsigned Error	2.13	3.23	7.78	5.80	12.1	10.6
Max Signed Error	0.887	3.23	7.78	1.17	12.1	10.6
Min Signed Error	-2.13	-2.27	-5.53	-5.80	-4.51	-10.1
Spearman ρ	1.00	1.00	0.999	0.999	0.999	0.999
Kendall τ	0.995	0.993	0.980	0.979	0.975	0.969
Spearman P-Value	0	0	0	0	0	0
Kendall τ P-Value	0	0	0	0	0	0
Perc. Chem. Acc.	1.00	1.00	0.999	0.994	0.990	0.973
Twice Perc. Chem. Acc.	1.00	1.00	1.00	1.00	1.00	1.00

Table S13: Statistical descriptors for QM gradient predictions on the respective test set by models trained according to entries 1–5 from Table 1 (see main text). Values related to gradients are reported in kJ mol^{-1} $Å^{-1}$.

	1	2	3	4	5
Mean Absolute Error	0.230	0.300	0.385	0.561	0.814
Median Absolute Error	0.156	0.203	0.257	0.369	0.551
Quantile 25	0.0699	0.0907	0.114	0.162	0.248
Quantile 75	0.304	0.401	0.510	0.742	1.07
RMSD	0.340	0.438	0.576	0.843	1.21
Max Unsigned Error	29.3	21.5	59.5	79.1	94.0
Max Signed Error	29.3	21.5	59.5	79.1	94.0
Min Signed Error	-22.8	-17.7	-48.4	-56.4	-66.9
Spearman ρ	1.00	1.00	1.00	1.00	1.00
Kendall τ	0.998	0.997	0.996	0.994	0.992
Spearman P-Value	0	0	0	0	0
Kendall τ P-Value	0	0	0	0	0

Table S14: Statistical descriptors for QM gradient predictions on the respective test set by models trained according to entries 6–11 from Table 1 (see main text). Values related to gradients are reported in kJ mol^{-1} $Å^{-1}$.

	6	7	8	9	10	11
Mean Absolute Error	0.296	0.397	0.878	0.711	1.05	1.17
Median Absolute Error	0.199	0.264	0.404	0.341	0.489	0.543
Quantile 25	0.0887	0.117	0.164	0.140	0.195	0.213
Quantile 75	0.394	0.526	1.01	0.815	1.24	1.40
RMSD	0.448	0.603	1.57	1.27	1.83	2.05
Max Unsigned Error	126	133	78.2	93.2	74.8	78.9
Max Signed Error	98.1	122	78.2	93.2	74.8	78.9
Min Signed Error	-126	-133	-52.5	-91.9	-57.4	-56.0
Spearman ρ	1.00	1.00	1.00	1.00	1.00	1.00
Kendall τ	0.997	0.996	0.990	0.992	0.989	0.987
Spearman P-Value	0	0	0	0	0	0
Kendall τ P-Value	0	0	0	0	0	0

Table S15: Statistical descriptors for MM gradient predictions on the respective test set by models trained according to entries 1–5 from Table 1 (see main text). Values related to gradients are reported in kJ mol^{-1} $Å^{-1}$.

	1	2	3	4	5
Mean Absolute Error	0.0130	0.0140	0.0151	0.0170	0.0192
Median Absolute Error	0.00856	0.00898	0.00932	0.00999	0.0106
Quantile 25	0.00379	0.00395	0.00408	0.00433	0.00457
Quantile 75	0.0168	0.0178	0.0187	0.0205	0.0220
RMSD	0.0196	0.0221	0.0251	0.0302	0.0373
Max Unsigned Error	2.42	2.92	5.93	5.80	10.2
Max Signed Error	2.42	2.70	2.93	5.47	7.91
Min Signed Error	-1.41	-2.92	-5.93	-5.8	-10.2
Spearman ρ	0.999	0.999	0.999	0.999	0.999
Kendall τ	0.983	0.983	0.982	0.980	0.979
Spearman P-Value	0	0	0	0	0
Kendall τ P-Value	0	0	0	0	0

Table S16: Statistical descriptors for MM gradient predictions on the respective test set by models trained according to entries 6–11 from Table 1 (see main text). Values related to gradients are reported in kJ mol^{-1} $Å^{-1}$.

	6	7	8	9	10	11
Mean Absolute Error	0.0140	0.0151	0.0167	0.0158	0.0189	0.0187
Median Absolute Error	0.00898	0.00934	0.00958	0.00941	0.0102	0.0101
Quantile 25	0.00396	0.00409	0.00418	0.00411	0.00442	0.00437
Quantile 75	0.0178	0.0188	0.0195	0.019	0.0212	0.0208
RMSD	0.0219	0.0251	0.0320	0.0283	0.0375	0.0385
Max Unsigned Error	6.90	7.15	9.43	10.1	8.44	8.61
Max Signed Error	2.83	7.15	8.71	10.0	8.44	6.99
Min Signed Error	-6.90	-6.52	-9.43	-10.1	-5.39	-8.61
Spearman ρ	0.999	0.999	0.999	0.999	0.999	0.999
Kendall τ	0.983	0.982	0.981	0.982	0.980	0.981
Spearman P-Value	0	0	0	0	0	0
Kendall τ P-Value	0	0	0	0	0	0

Table S17: Statistical descriptors for QM dipole predictions on the respective test set by models trained according to entries 1–5 from Table 1 (see main text). Values related to QM dipoles are reported in eÅ.

	1	2	3	4	5
Mean Absolute Error	0.0277	0.0281	0.0286	0.0297	0.0306
Median Absolute Error	0.0233	0.0237	0.0240	0.0248	0.0258
Quantile 25	0.0109	0.0110	0.0112	0.0117	0.0120
Quantile 75	0.0399	0.0405	0.0413	0.0426	0.0439
RMSD	0.0349	0.0354	0.0360	0.0374	0.0386
Max Unsigned Error	0.166	0.170	0.163	0.172	0.185
Max Signed Error	0.143	0.149	0.161	0.172	0.185
Min Signed Error	-0.166	-0.170	-0.163	-0.170	-0.173
Spearman ρ	0.999	0.999	0.999	0.999	0.999
Kendall τ	0.974	0.973	0.973	0.972	0.971
Spearman P-Value	0	0	0	0	0
Kendall τ P-Value	0	0	0	0	0

Table S18: Statistical descriptors for QM dipole predictions on the respective test set by models trained according to entries 6–11 from Table 1 (see main text). Values related to QM dipoles are reported in eÅ.

	6	7	8	9	10	11
Mean Absolute Error	0.0282	0.0286	0.0289	0.0288	0.0299	0.0298
Median Absolute Error	0.0238	0.0241	0.0243	0.0243	0.0251	0.025
Quantile 25	0.0111	0.0114	0.0115	0.0114	0.0118	0.0118
Quantile 75	0.0406	0.0412	0.0417	0.0414	0.0430	0.0428
RMSD	0.0355	0.0360	0.0364	0.0363	0.0377	0.0375
Max Unsigned Error	0.184	0.205	0.171	0.196	0.194	0.198
Max Signed Error	0.184	0.205	0.171	0.196	0.194	0.198
Min Signed Error	-0.165	-0.156	-0.161	-0.173	-0.171	-0.188
Spearman ρ	0.999	0.999	0.999	0.999	0.999	0.999
Kendall τ	0.973	0.973	0.973	0.973	0.972	0.974
Spearman P-Value	0	0	0	0	0	0
Kendall τ P-Value	0	0	0	0	0	0

Table S19: Statistical descriptors for QM quadrupole predictions on the respective test set by models trained according to entries 1–5 from Table 1 (see main text). Values related to QM quadrupoles are reported in eÅ^2.

	1	2	3	4	5
Mean Absolute Error	0.0188	0.0203	0.0223	0.0263	0.0302
Median Absolute Error	0.0155	0.0168	0.0185	0.0218	0.0251
Quantile 25	0.00737	0.00789	0.00875	0.0101	0.0118
Quantile 75	0.0268	0.0291	0.0317	0.0376	0.0432
RMSD	0.0239	0.0259	0.0283	0.0334	0.0384
Max Unsigned Error	0.121	0.142	0.143	0.174	0.199
Max Signed Error	0.121	0.142	0.143	0.174	0.170
Min Signed Error	-0.103	-0.135	-0.126	-0.163	-0.199
Spearman ρ	1.00	1.00	1.00	1.00	1.00
Kendall τ	0.989	0.988	0.987	0.985	0.982
Spearman P-Value	0	0	0	0	0
Kendall τ P-Value	0	0	0	0	0

Table S20: Statistical descriptors for QM quadrupole predictions on the respective test set by models trained according to entries 6–11 from Table 1 (see main text). Values related to QM quadrupoles are reported in eÅ^2.

	6	7	8	9	10	11
Mean Absolute Error	0.0209	0.0225	0.0397	0.0360	0.0425	0.0486
Median Absolute Error	0.0173	0.0186	0.0309	0.0278	0.0332	0.0364
Quantile 25	0.00811	0.00873	0.0141	0.0129	0.0153	0.0165
Quantile 75	0.0298	0.0321	0.0565	0.0502	0.0601	0.0680
RMSD	0.0265	0.0285	0.0519	0.0477	0.0556	0.0652
Max Unsigned Error	0.145	0.159	0.291	0.317	0.314	0.497
Max Signed Error	0.145	0.149	0.263	0.280	0.288	0.497
Min Signed Error	-0.135	-0.159	-0.291	-0.317	-0.314	-0.369
Spearman ρ	1.00	1.00	0.999	0.999	0.999	0.999
Kendall τ	0.988	0.987	0.977	0.978	0.975	0.969
Spearman P-Value	0	0	0	0	0	0
Kendall τ P-Value	0	0	0	0	0	0

S1.3 Alanine Dipeptide – GFN2-xTB

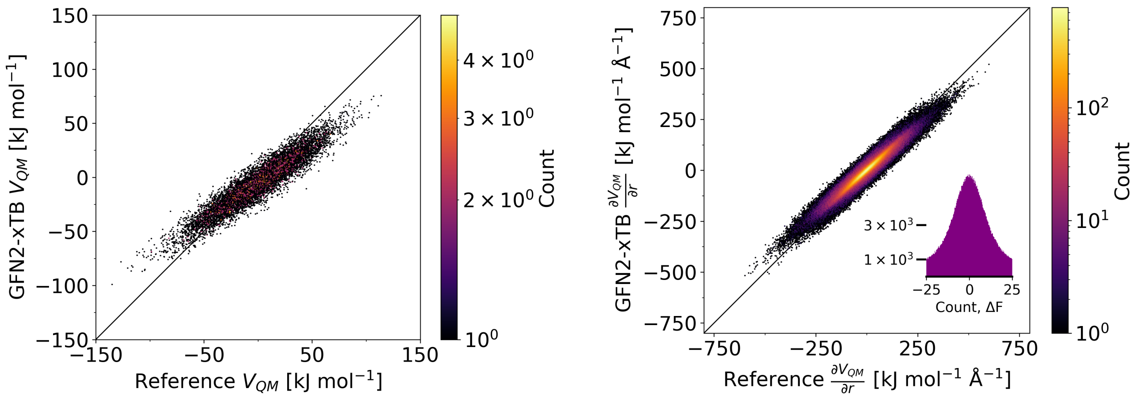

Figure S7: Correlation of GFN2-xTB energies and gradients and their respective references.

Table S21: Statistical descriptors for QM energy predictions of GFN2-xTB. Values related to energy are reported in kJ mol^{-1}. Percentage Chemical Accuracy and Twice Percentage Chemical Accuracy refer to percentage of predictions within 4.184 and 8.368 kJ mol^{-1}, respectively.

	GFN2-xTB
Mean Absolute Error	11.0
Median Absolute Error	9.33
Quantile 25	4.33
Quantile 75	15.9
RMSD	13.8
Max Unsigned Error	55.4
Max Signed Error	55.4
Min Signed Error	-49.3
Spearman ρ	0.933
Kendall τ	0.777
Spearman P-Value	0
Kendall τ P-Value	0
Perc. Chem. Acc.	0.241
Twice Perc. Chem. Acc.	0.457

Table S22: Statistical descriptors for QM gradient predictions of GFN2-xTB. Values related to gradients are reported in kJ mol^{-1} $Å^{-1}$.

	GFN2-xTB
Mean Absolute Error	16.0
Median Absolute Error	9.97
Quantile 25	4.34
Quantile 75	21.5
RMSD	23.1
Max Unsigned Error	150
Max Signed Error	150
Min Signed Error	-147
Spearman ρ	0.961
Kendall τ	0.845
Spearman P-Value	0
Kendall τ P-Value	0

Table S23: Statistical descriptors for MM gradient predictions of GFN2-xTB. Values related to gradients are reported in kJ mol^{-1} $Å^{-1}$.

	GFN2-xTB
Mean Absolute Error	0.186
Median Absolute Error	0.0372
Quantile 25	0.0154
Quantile 75	0.0923
RMSD	0.832
Max Unsigned Error	83.3
Max Signed Error	83.3
Min Signed Error	-71.7
Spearman ρ	0.978
Kendall τ	0.921
Spearman P-Value	0
Kendall τ P-Value	0

S1.4 Nickel Phosphine Complexes – AMP Model (600’000 Parameters)

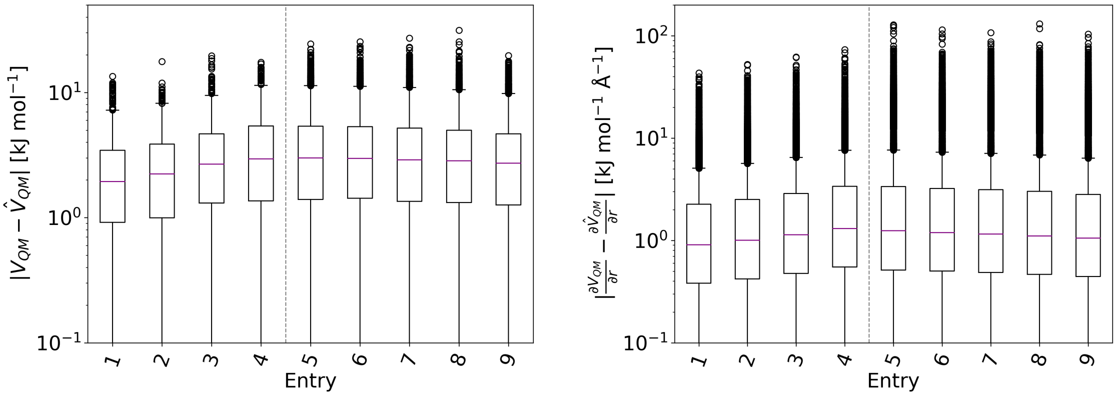

Figure S8: Distribution of the absolute errors of predicted QM energies and gradients of nickel complexes with respect to the reference for models trained on different training sets (see Table 3 in the main text for split definitions). All predictions were performed on the respective test sets.

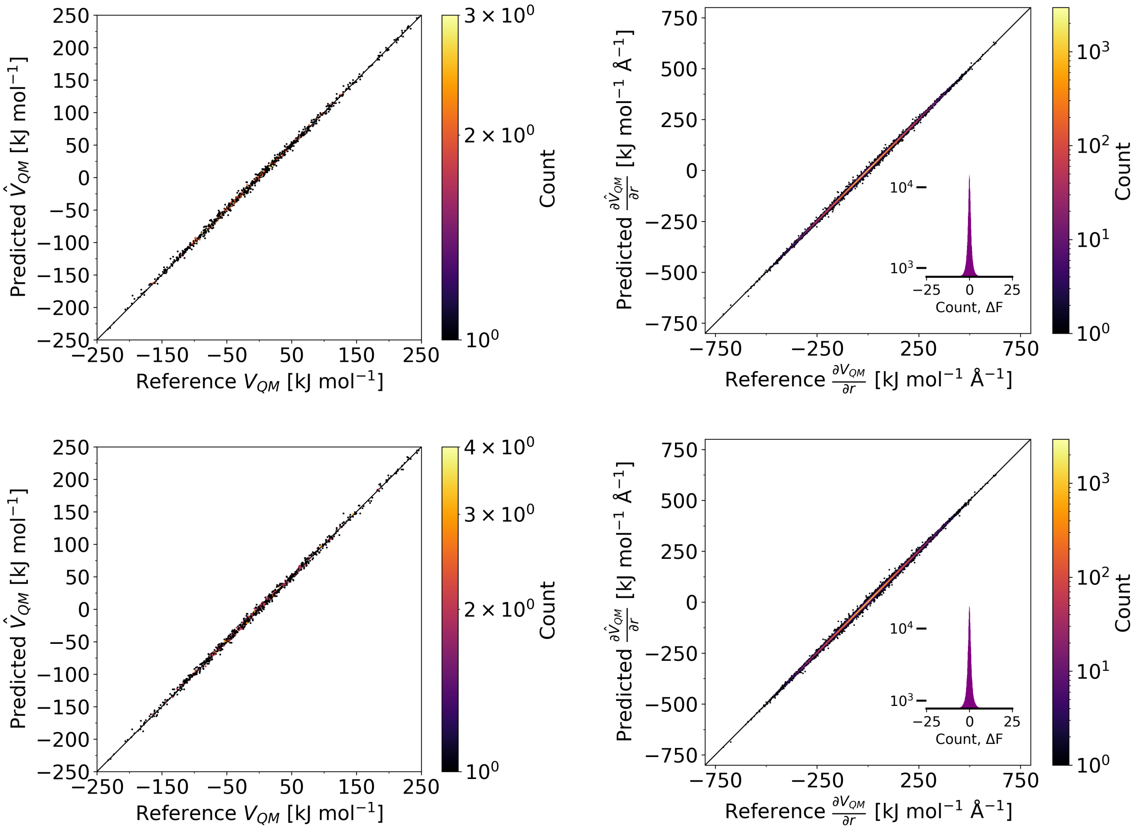

Figure S9: Correlation of predicted QM energies and gradients and their respective references using AMP. Top to bottom: entries 1–4 from Table 3 (see main text).

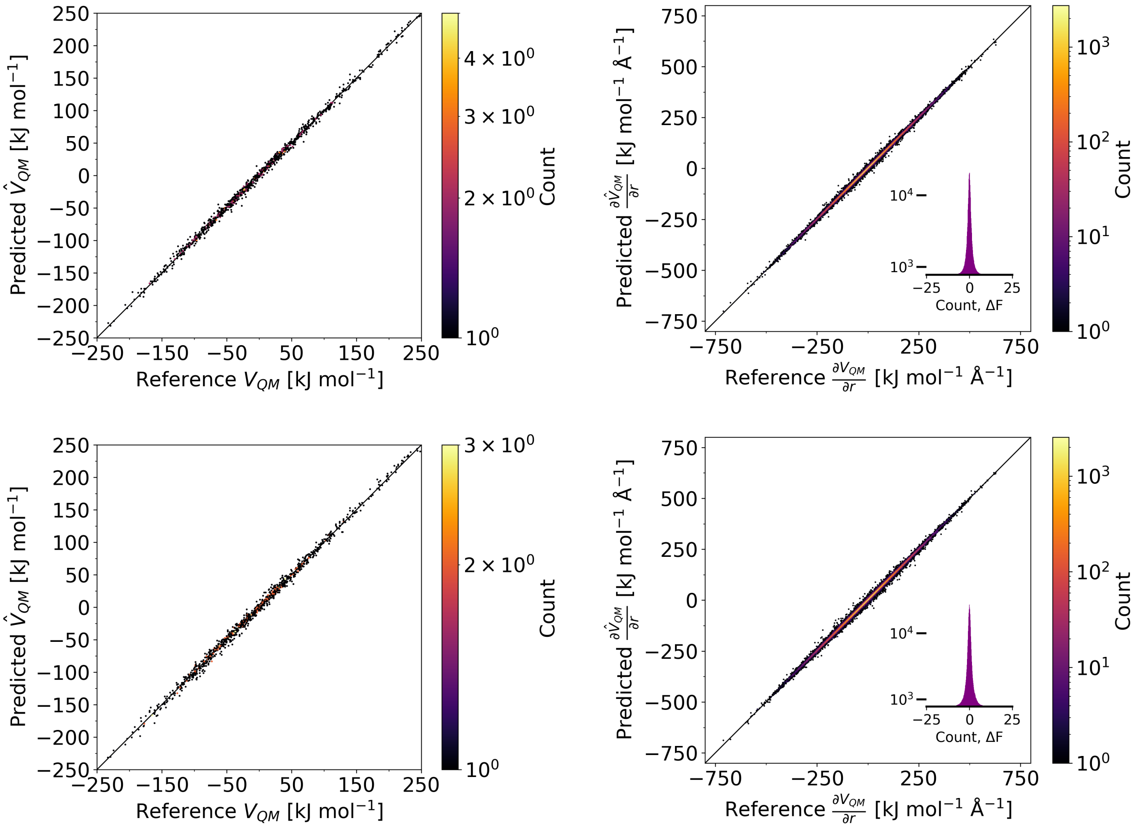

Figure S9: (cont.) Correlation of predicted QM energies and gradients and their respective references using AMP. Top to bottom: entries 1–4 from Table 3 (see main text).

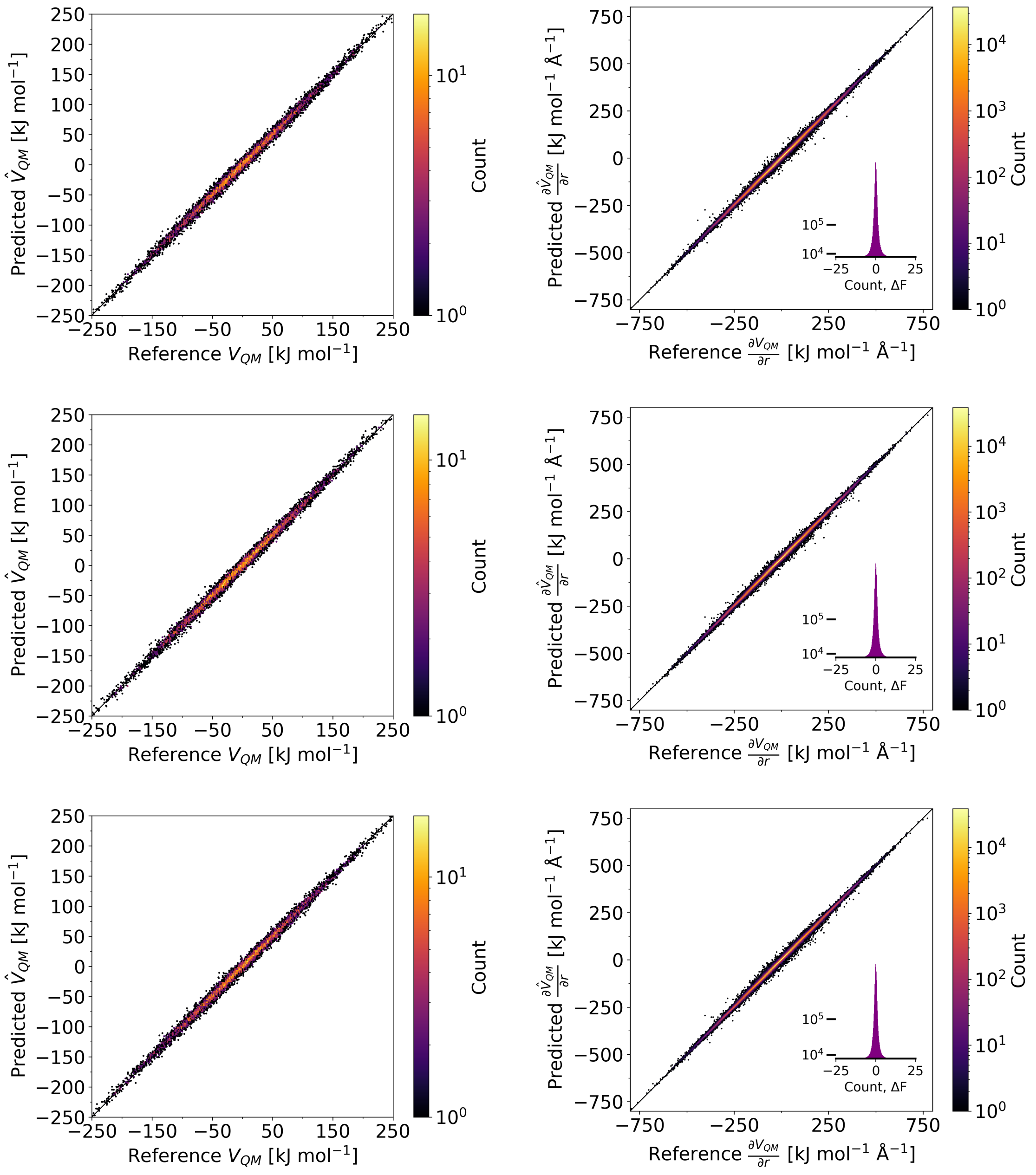

Figure S10: Correlation of predicted QM energies and gradients and their respective references using AMP. Top to bottom: entries 5–9 from Table 3 (see main text).

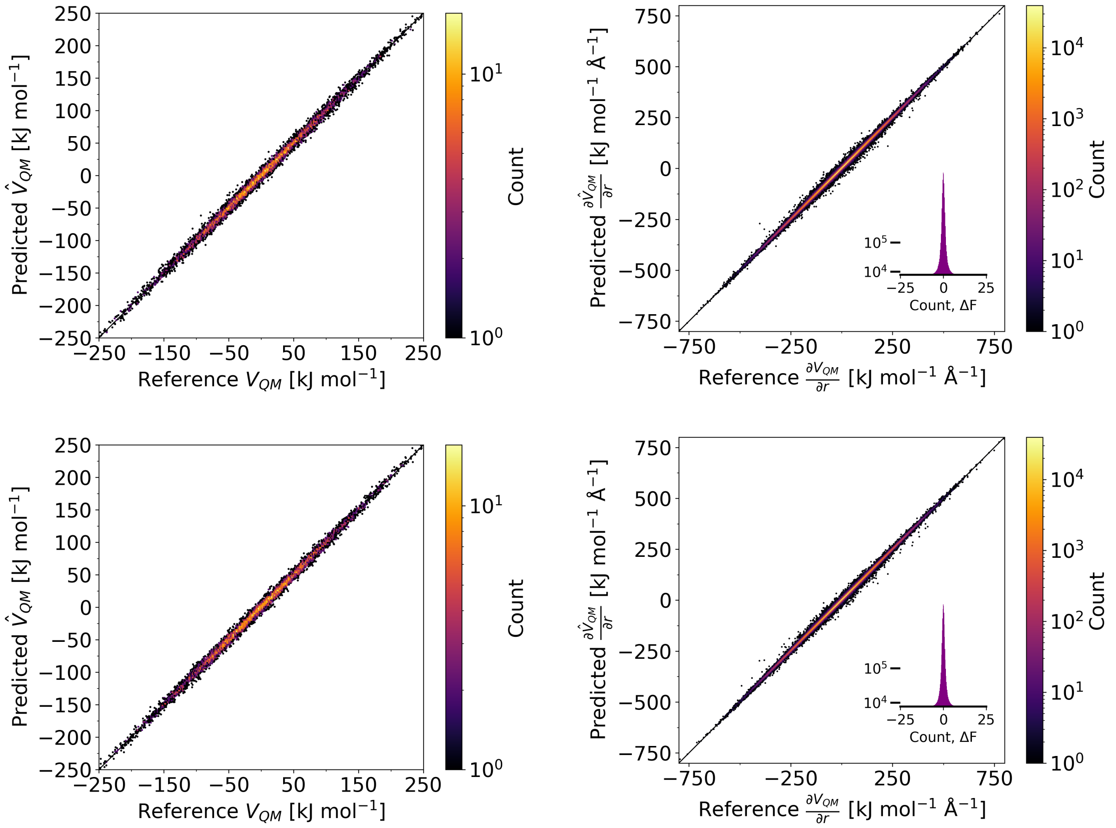

Figure S10: (cont.) Correlation of predicted QM energies and gradients and their respective references using AMP. Top to bottom: entries 5–9 from Table 3 (see main text).

Table S24: Statistical descriptors for QM energy predictions on the respective test set by models trained according to entries 1–4 from Table 3 (see main text). Values related to energy are reported in kJ mol^{-1}. Percentage Chemical Accuracy and Twice Percentage Chemical Accuracy refer to percentage of predictions within 4.184 and 8.368 kJ mol^{-1}, respectively.

	1	2	3	4
Mean Absolute Error	2.49	2.70	3.30	3.81
Median Absolute Error	1.94	2.23	2.68	2.95
Quantile 25	0.918	0.997	1.31	1.36
Quantile 75	3.45	3.87	4.68	5.40
RMSD	3.08	3.23	4.01	4.68
Max Unsigned Error	13.4	17.7	19.6	17.5
Max Signed Error	10.9	9.80	19.6	17.5
Min Signed Error	-13.4	-17.7	-18.7	-15.9
Spearman ρ	0.999	0.999	0.998	0.998
Kendall τ	0.976	0.975	0.969	0.962
Spearman P-Value	0	0	0	0
Kendall τ P-Value	0	0	0	0
Perc. Chem. Acc.	0.839	0.798	0.707	0.634
Twice Perc. Chem. Acc.	0.970	0.980	0.949	0.906

Table S25: Statistical descriptors for QM energy predictions on the respective test set by models trained according to entries 5–9 from Table 3 (see main text). Values related to energy are reported in kJ mol^{-1}. Percentage Chemical Accuracy and Twice Percentage Chemical Accuracy refer to percentage of predictions within 4.184 and 8.368 kJ mol^{-1}, respectively.

	5	6	7	8	9
Mean Absolute Error	3.82	3.79	3.68	3.53	3.33
Median Absolute Error	2.99	2.97	2.89	2.85	2.73
Quantile 25	1.39	1.43	1.35	1.32	1.26
Quantile 75	5.37	5.34	5.20	5.00	4.68
RMSD	4.66	4.61	4.5	4.29	4.00
Max Unsigned Error	24.5	25.5	27.1	31.3	19.7
Max Signed Error	24.5	22.6	19.9	21.0	19.7
Min Signed Error	-22.0	-25.5	-27.1	-31.3	-17.3
Spearman ρ	0.998	0.998	0.998	0.998	0.998
Kendall τ	0.959	0.960	0.961	0.963	0.965
Spearman P-Value	0	0	0	0	0
Kendall τ P-Value	0	0	0	0	0
Perc. Chem. Acc.	0.642	0.643	0.658	0.669	0.692
Twice Perc. Chem. Acc.	0.904	0.910	0.914	0.932	0.946

Table S26: Statistical descriptors for QM gradient predictions on the respective test set by models trained according to entries 1–4 from Table 3 (see main text). Values related to gradients are reported in kJ mol^{-1} $Å^{-1}$.

	1	2	3	4
Mean Absolute Error	1.08	1.20	1.39	1.65
Median Absolute Error	0.707	0.774	0.885	1.03
Quantile 25	0.315	0.344	0.393	0.458
Quantile 75	1.39	1.52	1.75	2.08
RMSD	1.66	1.90	2.23	2.65
Max Unsigned Error	43.4	52.6	61.9	73.1
Max Signed Error	36.6	45.6	51.6	68.5
Min Signed Error	-43.4	-52.6	-61.9	-73.1
Spearman ρ	1.00	1.00	1.00	1.00
Kendall τ	0.990	0.989	0.987	0.985
Spearman P-Value	0	0	0	0
Kendall τ P-Value	0	0	0	0

Table S27: Statistical descriptors for QM gradient predictions on the respective test set by models trained according to entries 5–9 from Table 3 (see main text). Values related to gradients are reported in kJ mol^{-1} $Å^{-1}$.

	5	6	7	8	9
Mean Absolute Error	1.47	1.40	1.36	1.31	1.24
Median Absolute Error	0.931	0.897	0.870	0.837	0.798
Quantile 25	0.409	0.401	0.388	0.373	0.357
Quantile 75	1.88	1.77	1.71	1.65	1.57
RMSD	2.32	2.20	2.16	2.07	1.95
Max Unsigned Error	128	114	107	131	104
Max Signed Error	125	114	87.3	117	96.6
Min Signed Error	-128	-94.5	-107	-131	-104
Spearman ρ	1.00	1.00	1.00	1.00	1.00
Kendall τ	0.986	0.987	0.987	0.988	0.989
Spearman P-Value	0	0	0	0	0
Kendall τ P-Value	0	0	0	0	0

Table S28: Statistical descriptors for MM gradient predictions on the respective test set by models trained according to entries 1–4 from Table 3 (see main text). Values related to gradients are reported in kJ mol^{-1} $Å^{-1}$.

	1	2	3	4
Mean Absolute Error	0.0111	0.0121	0.0130	0.0150
Median Absolute Error	0.00310	0.00353	0.00382	0.00423
Quantile 25	0.00126	0.00143	0.00156	0.00172
Quantile 75	0.00812	0.00913	0.00959	0.0108
RMSD	0.0307	0.0336	0.0367	0.0430
Max Unsigned Error	5.03	4.53	5.35	6.13
Max Signed Error	5.03	4.19	5.15	6.13
Min Signed Error	-3.66	-4.53	-5.35	-5.61
Spearman ρ	0.987	0.985	0.983	0.977
Kendall τ	0.936	0.93	0.924	0.912
Spearman P-Value	0	0	0	0
Kendall τ P-Value	0	0	0	0

Table S29: Statistical descriptors for MM gradient predictions on the respective test set by models trained according to entries 5–9 from Table 3 (see main text). Values related to gradients are reported in kJ mol^{-1} $Å^{-1}$.

	5	6	7	8	9
Mean Absolute Error	0.0122	0.0117	0.0114	0.0114	0.0115
Median Absolute Error	0.00370	0.00343	0.00344	0.00346	0.00346
Quantile 25	0.00151	0.00140	0.00141	0.00141	0.00141
Quantile 75	0.00940	0.00893	0.00860	0.00877	0.00883
RMSD	0.0325	0.0317	0.0315	0.0311	0.0308
Max Unsigned Error	9.25	9.46	8.83	9.32	5.79
Max Signed Error	5.16	6.40	8.83	6.35	5.79
Min Signed Error	-9.25	-9.46	-7.09	-9.32	-4.62
Spearman ρ	0.980	0.981	0.981	0.982	0.982
Kendall τ	0.915	0.920	0.920	0.922	0.922
Spearman P-Value	0	0	0	0	0
Kendall τ P-Value	0	0	0	0	0

Table S30: Statistical descriptors for QM dipole predictions on the respective test set by models trained according to entries 1–4 from Table 3 (see main text). Values related to QM dipoles are reported in eÅ.

	1	2	3	4
Mean Absolute Error	0.0415	0.0494	0.0577	0.0663
Median Absolute Error	0.0330	0.0385	0.0451	0.0526
Quantile 25	0.0150	0.0189	0.0211	0.0236
Quantile 75	0.0580	0.0681	0.0791	0.0925
RMSD	0.0549	0.0650	0.0770	0.0877
Max Unsigned Error	0.308	0.316	0.616	0.426
Max Signed Error	0.282	0.316	0.616	0.426
Min Signed Error	-0.308	-0.315	-0.339	-0.381
Spearman ρ	0.997	0.995	0.993	0.992
Kendall τ	0.951	0.942	0.932	0.922
Spearman P-Value	0	0	0	0
Kendall τ P-Value	0	0	0	0

Table S31: Statistical descriptors for QM dipole predictions on the respective test set by models trained according to entries 5–9 from Table 3 (see main text). Values related to QM dipoles are reported in eÅ.

	5	6	7	8	9
Mean Absolute Error	0.0560	0.0527	0.0556	0.0505	0.0507
Median Absolute Error	0.0437	0.0416	0.0435	0.0402	0.0403
Quantile 25	0.0203	0.0195	0.0203	0.0187	0.0187
Quantile 75	0.0770	0.0738	0.0772	0.0710	0.0703
RMSD	0.0748	0.0694	0.0739	0.0661	0.0665
Max Unsigned Error	0.590	0.387	0.562	0.427	0.444
Max Signed Error	0.464	0.387	0.518	0.427	0.418
Min Signed Error	-0.590	-0.380	-0.562	-0.408	-0.444
Spearman ρ	0.994	0.995	0.994	0.995	0.995
Kendall τ	0.935	0.939	0.935	0.941	0.941
Spearman P-Value	0	0	0	0	0
Kendall τ P-Value	0	0	0	0	0

Table S32: Statistical descriptors for QM quadrupole predictions on the respective test set by models trained according to entries 1–4 from Table 3 (see main text). Values related to QM quadrupoles are reported in eÅ^2.

	1	2	3	4
Mean Absolute Error	0.188	0.203	0.226	0.258
Median Absolute Error	0.141	0.152	0.169	0.192
Quantile 25	0.0661	0.0697	0.0770	0.0891
Quantile 75	0.258	0.275	0.311	0.360
RMSD	0.255	0.276	0.309	0.350
Max Unsigned Error	1.59	1.99	1.80	2.04
Max Signed Error	1.47	1.99	1.80	2.04
Min Signed Error	-1.59	-1.54	-1.62	-1.95
Spearman ρ	0.997	0.996	0.995	0.994
Kendall τ	0.954	0.951	0.944	0.935
Spearman P-Value	0	0	0	0
Kendall τ P-Value	0	0	0	0

Table S33: Statistical descriptors for QM quadrupole predictions on the respective test set by models trained according to entries 5–9 from Table 3 (see main text). Values related to QM quadrupoles are reported in eÅ^2.

	5	6	7	8	9
Mean Absolute Error	0.228	0.224	0.226	0.210	0.210
Median Absolute Error	0.171	0.168	0.169	0.159	0.157
Quantile 25	0.0787	0.0771	0.0774	0.0730	0.0726
Quantile 75	0.313	0.308	0.310	0.291	0.291
RMSD	0.309	0.303	0.307	0.284	0.284
Max Unsigned Error	2.58	2.14	2.36	2.00	2.04
Max Signed Error	2.12	1.86	2.36	1.96	1.93
Min Signed Error	-2.58	-2.14	-2.08	-2.00	-2.04
Spearman ρ	0.995	0.996	0.995	0.996	0.996
Kendall τ	0.944	0.945	0.944	0.948	0.948
Spearman P-Value	0	0	0	0	0
Kendall τ P-Value	0	0	0	0	0

S1.5 Nickel Phosphine Complexes – AMP Model (2.7 Million Parameters)

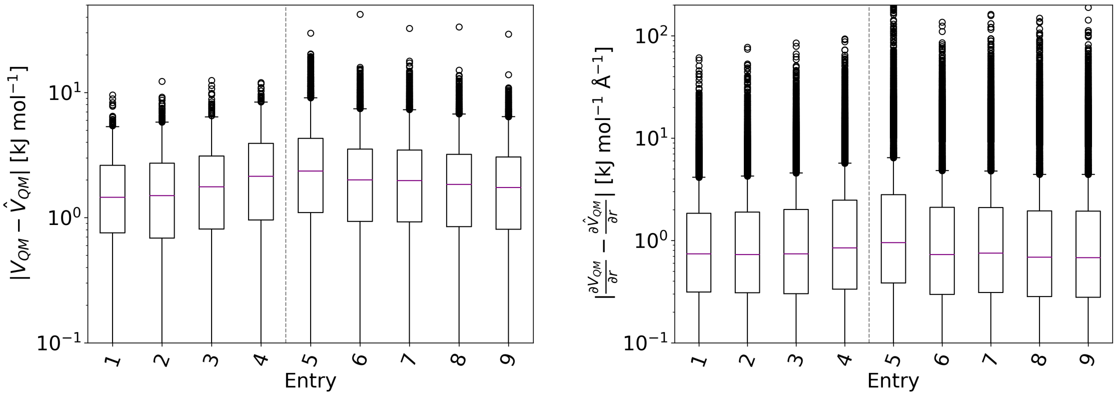

Figure S11: Distribution of the absolute errors of predicted QM energies and gradients of nickel complexes with respect to the reference for models trained on different training sets (see Table 3 in the main text for split definitions). All predictions were performed on the respective test sets.

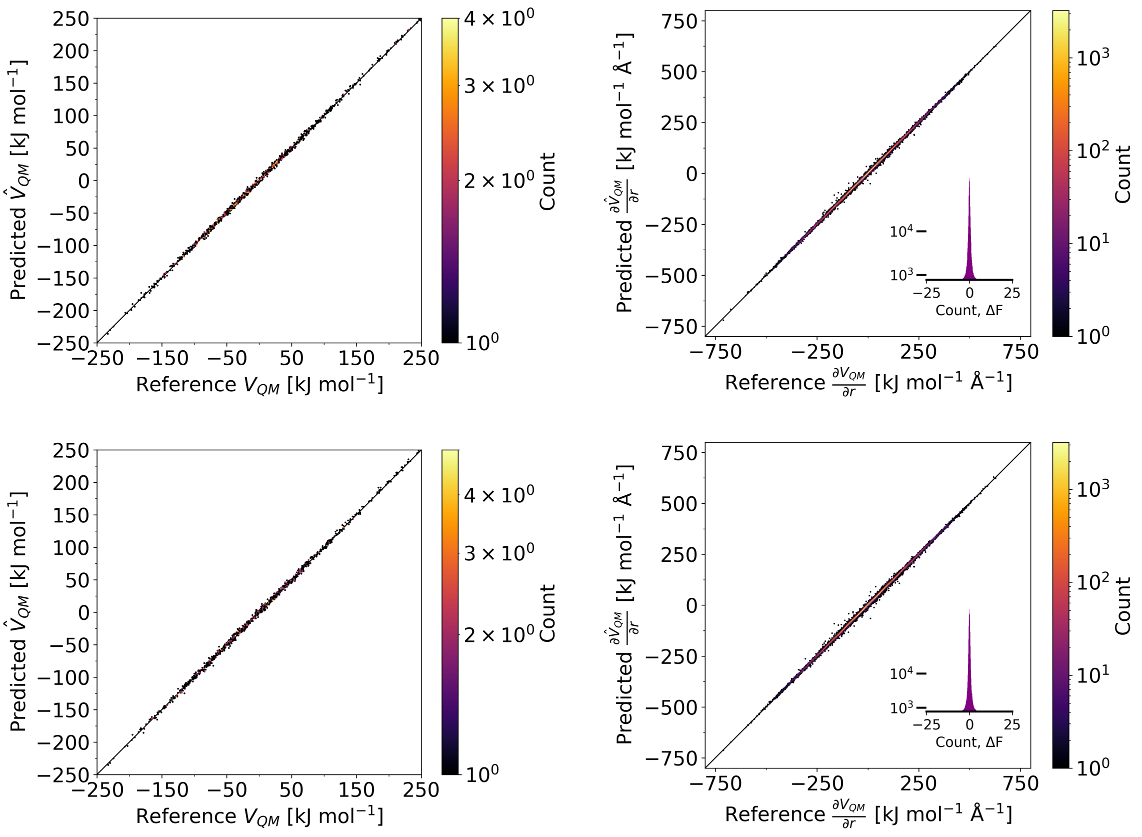

Figure S12: Correlation of predicted QM energies and gradients and their respective references using AMP. Top to bottom: entries 1–4 from Table 3 (see main text).

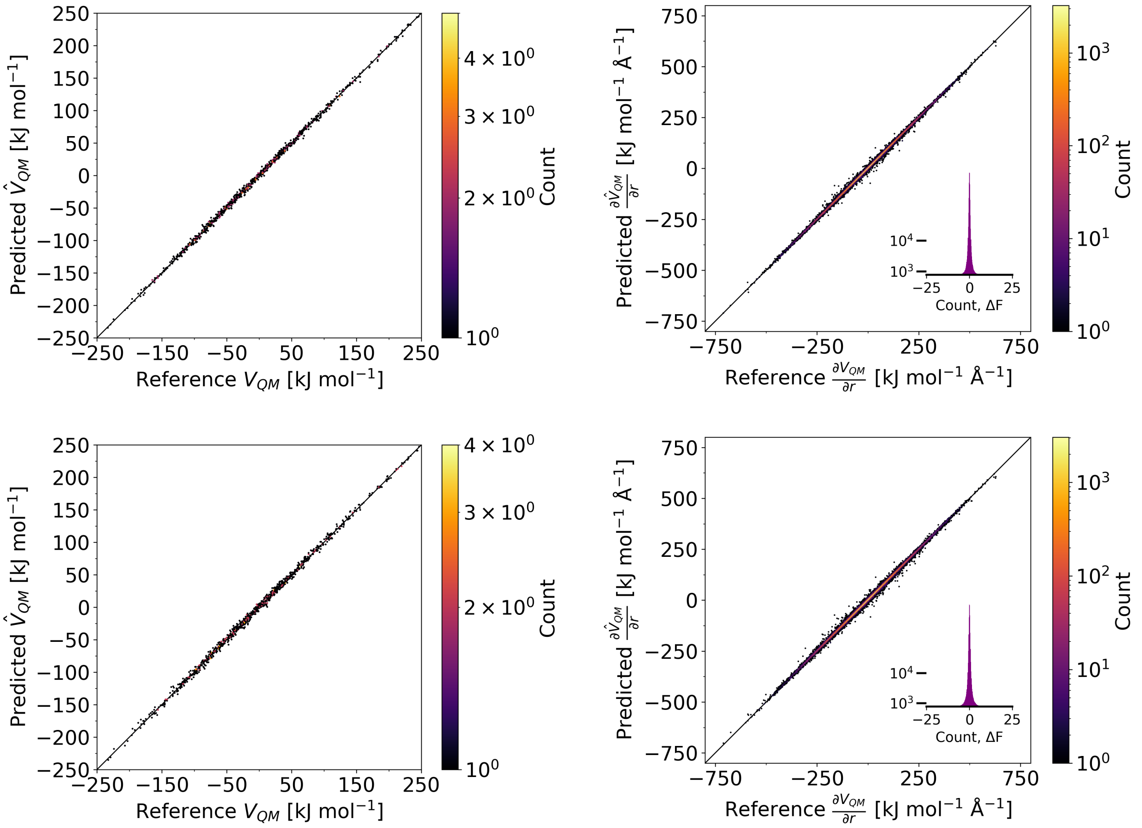

Figure S12: (cont.) Correlation of predicted QM energies and gradients and their respective references using AMP. Top to bottom: entries 1–4 from Table 3 (see main text).

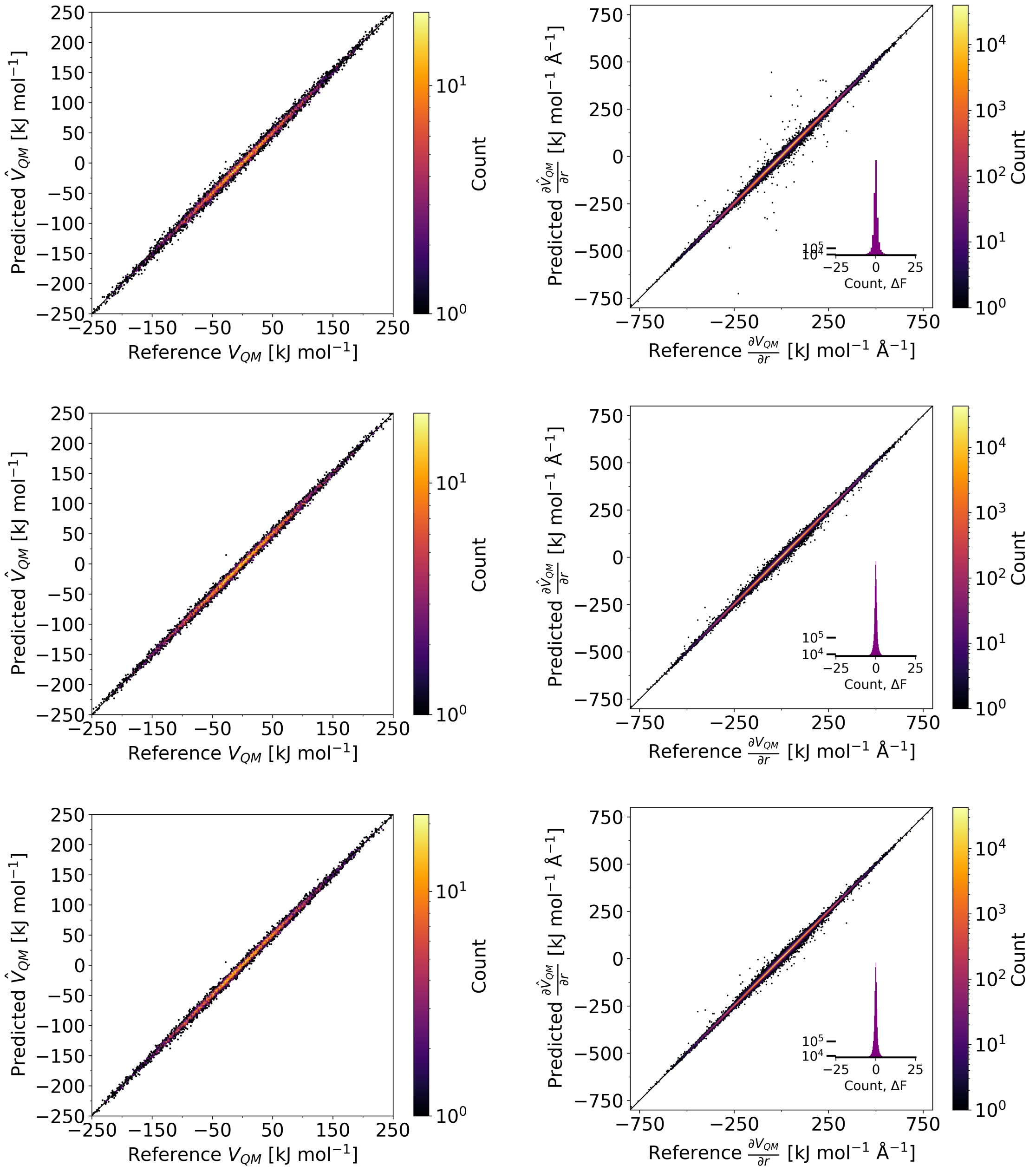

Figure S13: Correlation of predicted QM energies and gradients and their respective references using AMP. Top to bottom: entries 5–9 from Table 3 (see main text).

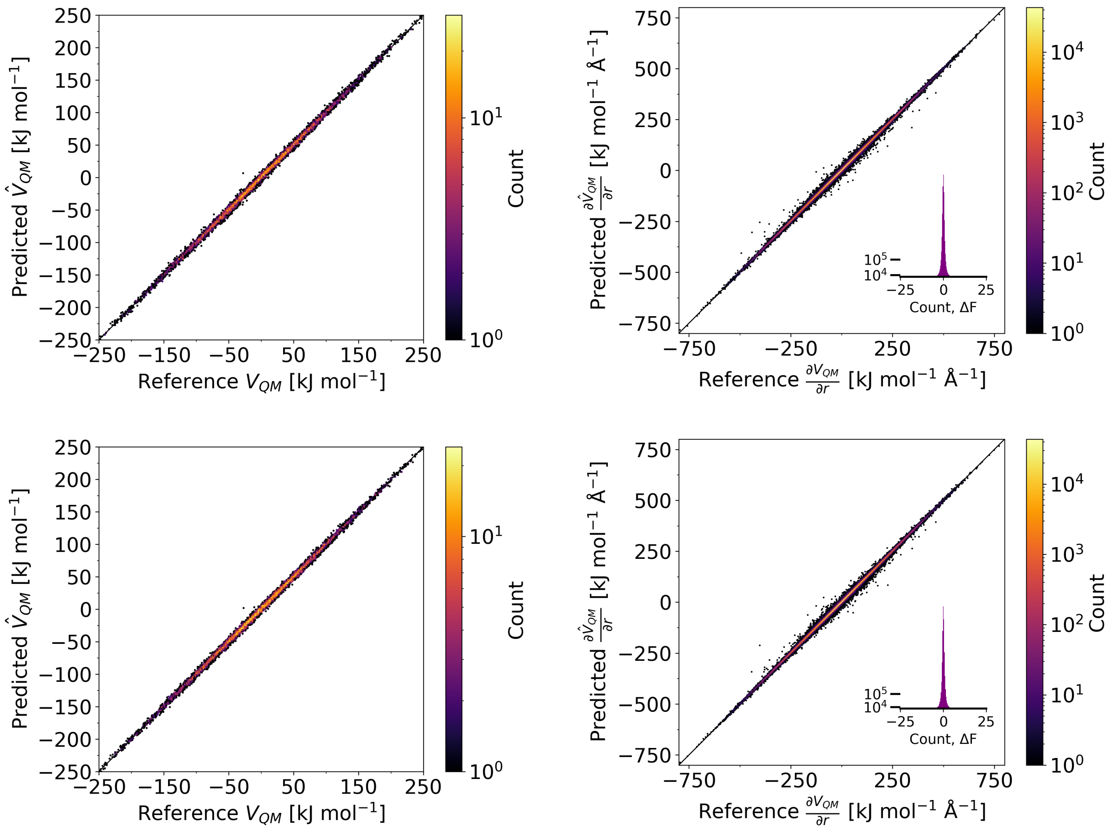

Figure S13: (cont.) Correlation of predicted QM energies and gradients and their respective references using AMP. Top to bottom: entries 5–9 from Table 3 (see main text).

Table S34: Statistical descriptors for QM energy predictions on the respective test set by models trained according to entries 1–4 from Table 3 (see main text). Values related to energy are reported in kJ mol^{-1}. Percentage Chemical Accuracy and Twice Percentage Chemical Accuracy refer to percentage of predictions within 4.184 and 8.368 kJ mol^{-1}, respectively.

	1	2	3	4
Mean Absolute Error	1.83	1.90	2.20	2.70
Median Absolute Error	1.45	1.50	1.76	2.14
Quantile 25	0.756	0.686	0.812	0.956
Quantile 75	2.61	2.73	3.10	3.93
RMSD	2.18	2.35	2.67	3.25
Max Unsigned Error	9.53	12.3	12.5	12.0
Max Signed Error	7.78	12.3	12.5	11.7
Min Signed Error	-9.53	-8.99	-11.4	-12.0
Spearman ρ	1.00	0.999	0.999	0.999
Kendall τ	0.982	0.981	0.978	0.973
Spearman P-Value	0	0	0	0
Kendall τ P-Value	0	0	0	0
Perc. Chem. Acc.	0.923	0.910	0.864	0.770
Twice Perc. Chem. Acc.	0.998	0.996	0.992	0.978

Table S35: Statistical descriptors for QM energy predictions on the respective test set by models trained according to entries 5–9 from Table 3 (see main text). Values related to energy are reported in kJ mol^{-1}. Percentage Chemical Accuracy and Twice Percentage Chemical Accuracy refer to percentage of predictions within 4.184 and 8.368 kJ mol^{-1}, respectively.

	5	6	7	8	9
Mean Absolute Error	3.09	2.56	2.50	2.26	2.15
Median Absolute Error	2.36	2.00	1.98	1.84	1.74
Quantile 25	1.10	0.931	0.923	0.845	0.808
Quantile 75	4.29	3.53	3.47	3.20	3.05
RMSD	3.87	3.17	3.08	2.76	2.59
Max Unsigned Error	29.7	42.1	32.6	33.5	29.3
Max Signed Error	20.3	15.4	17.8	13.7	10.9
Min Signed Error	-29.7	-42.1	-32.6	-33.5	-29.3
Spearman ρ	0.998	0.999	0.999	0.999	0.999
Kendall τ	0.967	0.973	0.973	0.976	0.977
Spearman P-Value	0	0	0	0	0
Kendall τ P-Value	0	0	0	0	0
Perc. Chem. Acc.	0.740	0.819	0.824	0.860	0.875
Twice Perc. Chem. Acc.	0.946	0.976	0.979	0.990	0.994

Table S36: Statistical descriptors for QM gradient predictions on the respective test set by models trained according to entries 1–4 from Table 3 (see main text). Values related to gradients are reported in kJ mol^{-1} $Å^{-1}$.

	1	2	3	4
Mean Absolute Error	0.878	0.905	0.964	1.15
Median Absolute Error	0.570	0.566	0.558	0.635
Quantile 25	0.254	0.252	0.243	0.270
Quantile 75	1.12	1.13	1.16	1.36
RMSD	1.39	1.51	1.72	2.10
Max Unsigned Error	61.0	77.4	85.0	93.3
Max Signed Error	61.0	77.4	85.0	93.3
Min Signed Error	-58.0	-73.9	-79.1	-91.8
Spearman ρ	1.00	1.00	1.00	1.00
Kendall τ	0.992	0.992	0.991	0.989
Spearman P-Value	0	0	0	0
Kendall τ P-Value	0	0	0	0

Table S37: Statistical descriptors for QM gradient predictions on the respective test set by models trained according to entries 5–9 from Table 3 (see main text). Values related to gradients are reported in kJ mol^{-1} $Å^{-1}$.

	5	6	7	8	9
Mean Absolute Error	1.17	0.901	0.917	0.839	0.822
Median Absolute Error	0.699	0.541	0.565	0.513	0.503
Quantile 25	0.307	0.238	0.253	0.227	0.223
Quantile 75	1.44	1.10	1.12	1.03	1.01
RMSD	2.06	1.56	1.57	1.45	1.42
Max Unsigned Error	498	136	162	149	189
Max Signed Error	497	125	158	135	143
Min Signed Error	-498	-136	-162	-149	-189
Spearman ρ	1.00	1.00	1.00	1.00	1.00
Kendall τ	0.989	0.992	0.991	0.992	0.992
Spearman P-Value	0	0	0	0	0
Kendall τ P-Value	0	0	0	0	0

Table S38: Statistical descriptors for MM gradient predictions on the respective test set by models trained according to entries 1–4 from Table 3 (see main text). Values related to gradients are reported in kJ mol^{-1} Å^{-1}.

	1	2	3	4
Mean Absolute Error	0.0100	0.00982	0.0103	0.0108
Median Absolute Error	0.00347	0.00342	0.00351	0.00364
Quantile 25	0.00140	0.00140	0.00143	0.00150
Quantile 75	0.00874	0.00839	0.00853	0.00875
RMSD	0.0248	0.0256	0.0281	0.0297
Max Unsigned Error	3.70	4.25	6.02	5.05
Max Signed Error	3.70	4.25	6.02	5.05
Min Signed Error	-3.33	-3.07	-4.27	-4.21
Spearman ρ	0.989	0.990	0.989	0.988
Kendall τ	0.936	0.938	0.936	0.933
Spearman P-Value	0	0	0	0
Kendall τ P-Value	0	0	0	0

Table S39: Statistical descriptors for MM gradient predictions on the respective test set by models trained according to entries 5–9 from Table 3 (see main text). Values related to gradients are reported in kJ mol^{-1} Å^{-1}.

	5	6	7	8	9
Mean Absolute Error	0.00927	0.00899	0.00927	0.00893	0.00879
Median Absolute Error	0.00293	0.00325	0.00324	0.00322	0.00314
Quantile 25	0.00121	0.00134	0.00133	0.00132	0.00129
Quantile 75	0.00725	0.00782	0.00786	0.00780	0.00763
RMSD	0.0252	0.0228	0.0238	0.0228	0.0222
Max Unsigned Error	16.3	4.72	5.28	4.92	5.37
Max Signed Error	16.3	3.78	5.28	4.92	4.69
Min Signed Error	-10.9	-4.72	-4.39	-4.61	-5.37
Spearman ρ	0.987	0.988	0.988	0.989	0.989
Kendall τ	0.935	0.934	0.932	0.934	0.935
Spearman P-Value	0	0	0	0	0
Kendall τ P-Value	0	0	0	0	0

Table S40: Statistical descriptors for QM dipole predictions on the respective test set by models trained according to entries 1–4 from Table 3 (see main text). Values related to QM dipoles are reported in eÅ.

	1	2	3	4
Mean Absolute Error Mean Absolute Error	0.0367	0.0408	0.0458	0.0531
Median Absolute Error	0.0291	0.0325	0.0362	0.0417
Quantile 25	0.0142	0.0154	0.0168	0.0191
Quantile 75	0.0496	0.0566	0.0643	0.0758
RMSD	0.0487	0.0534	0.0603	0.0697
Max Unsigned Error	0.270	0.275	0.300	0.361
Max Signed Error	0.259	0.210	0.279	0.358
Min Signed Error	-0.270	-0.275	-0.300	-0.361
Spearman ρ	0.997	0.997	0.996	0.995
Kendall τ	0.957	0.952	0.946	0.937
Spearman P-Value	0	0	0	0
Kendall τ P-Value	0	0	0	0

Table S41: Statistical descriptors for QM dipole predictions on the respective test set by models trained according to entries 5–9 from Table 3 (see main text). Values related to QM dipoles are reported in eÅ.

	5	6	7	8	9
Mean Absolute Error	0.0446	0.0434	0.0430	0.0423	0.0417
Median Absolute Error	0.0361	0.0346	0.0346	0.0341	0.0337
Quantile 25	0.0168	0.0161	0.0163	0.0162	0.0157
Quantile 75	0.0632	0.0611	0.0604	0.0592	0.0586
RMSD	0.0578	0.0566	0.0558	0.0550	0.0541
Max Unsigned Error	0.389	0.351	0.331	0.414	0.349
Max Signed Error	0.389	0.351	0.302	0.278	0.313
Min Signed Error	-0.291	-0.315	-0.331	-0.414	-0.349
Spearman ρ	0.997	0.997	0.997	0.997	0.997
Kendall τ	0.948	0.949	0.950	0.951	0.952
Spearman P-Value	0	0	0	0	0
Kendall τ P-Value	0	0	0	0	0

Table S42: Statistical descriptors for QM quadrupole predictions on the respective test set by models trained according to entries 1–4 from Table 3 (see main text). Values related to QM quadrupoles are reported in eÅ^2.

	1	2	3	4
Mean Absolute Error	0.163	0.176	0.193	0.217
Median Absolute Error	0.122	0.131	0.142	0.163
Quantile 25	0.0559	0.0608	0.0661	0.0763
Quantile 75	0.223	0.240	0.267	0.297
RMSD	0.222	0.241	0.265	0.294
Max Unsigned Error	1.63	1.60	1.91	2.24
Max Signed Error	1.25	1.60	1.91	2.24
Min Signed Error	-1.63	-1.37	-1.91	-1.66
Spearman ρ	0.998	0.997	0.997	0.996
Kendall τ	0.960	0.956	0.952	0.946
Spearman P-Value	0	0	0	0
Kendall τ P-Value	0	0	0	0

Table S43: Statistical descriptors for QM quadrupole predictions on the respective test set by models trained according to entries 5–9 from Table 3 (see main text). Values related to QM quadrupoles are reported in eÅ^2.

	5	6	7	8	9
Mean Absolute Error	0.189	0.187	0.185	0.181	0.177
Median Absolute Error	0.142	0.142	0.140	0.138	0.134
Quantile 25	0.0656	0.0643	0.0646	0.0630	0.0621
Quantile 75	0.260	0.258	0.255	0.248	0.245
RMSD	0.254	0.253	0.249	0.243	0.238
Max Unsigned Error	2.11	1.85	1.65	1.74	2.06
Max Signed Error	2.11	1.64	1.47	1.60	1.81
Min Signed Error	-1.83	-1.85	-1.65	-1.74	-2.06
Spearman ρ	0.997	0.997	0.997	0.997	0.997
Kendall τ	0.954	0.954	0.954	0.956	0.957
Spearman P-Value	0	0	0	0	0
Kendall τ P-Value	0	0	0	0	0

S1.6 Nickel Phosphine Complexes – GFN2-xTB

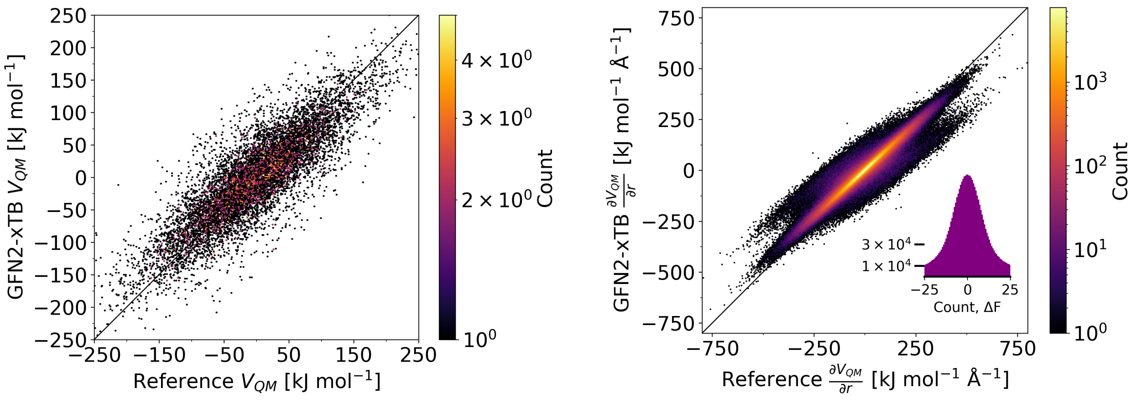

Figure S14: Correlation of GFN2-xTB energies and gradients and their respective references.

Table S44: Statistical descriptors for QM energy predictions of GFN2-xTB. Values related to energy are reported in kJ mol^{-1}. Percentage Chemical Accuracy and Twice Percentage Chemical Accuracy refer to percentage of predictions within 4.184 and 8.368 kJ mol^{-1}, respectively.

	GFN2-xTB
Mean Absolute Error	30.1
Median Absolute Error	24.3
Quantile 25	10.8
Quantile 75	44.4
RMSD	36.2
Max Unsigned Error	140
Max Signed Error	140
Min Signed Error	-127
Spearman ρ	0.868
Kendall τ	0.690
Spearman P-Value	0
Kendall τ P-Value	0
Perc. Chem. Acc.	0.0933
Twice Perc. Chem. Acc.	0.195

Table S45: Statistical descriptors for QM gradient predictions of GFN2-xTB. Values related to gradients are reported in kJ mol^{-1} $Å^{-1}$.

	GFN2-xTB
Mean Absolute Error	14.4
Median Absolute Error	8.38
Quantile 25	3.73
Quantile 75	17.6
RMSD	24.2
Max Unsigned Error	325
Max Signed Error	325
Min Signed Error	-303
Spearman ρ	0.970
Kendall τ	0.879
Spearman P-Value	0
Kendall τ P-Value	0

Table S46: Statistical descriptors for MM gradient predictions of GFN2-xTB. Values related to gradients are reported in kJ mol^{-1} $Å^{-1}$.

	GFN2-xTB
Mean Absolute Error	0.0503
Median Absolute Error	0.0131
Quantile 25	0.00539
Quantile 75	0.0331
RMSD	0.142
Max Unsigned Error	17.7
Max Signed Error	17.7
Min Signed Error	-16.8
Spearman ρ	0.848
Kendall τ	0.725
Spearman P-Value	0
Kendall τ P-Value	0

S1.7 Pyridine and Quinoline Dimers – AMP Model (600’000 Parameters)

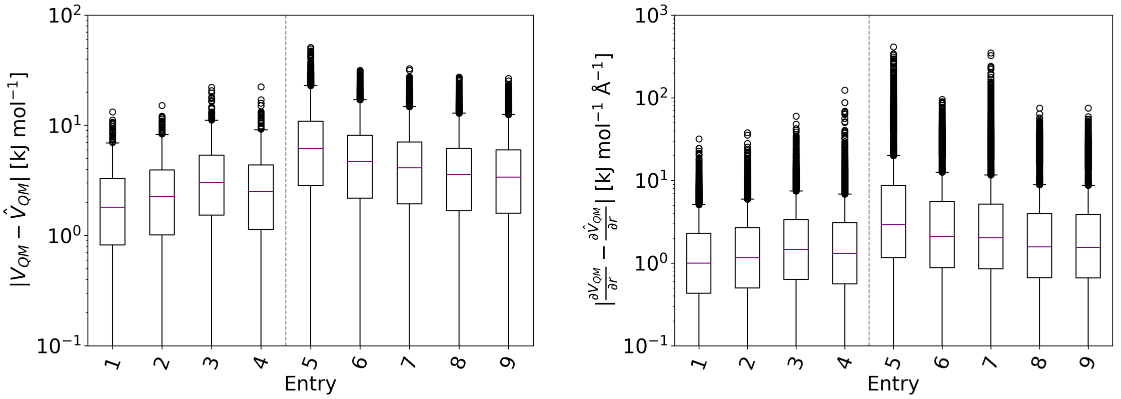

Figure S15: Distribution of the absolute errors of predicted QM energies and gradients of pyridine and quinoline dimers with respect to the reference for models trained on different training sets (see Table 4 in the main text for split definitions). All predictions were performed on the respective test sets.

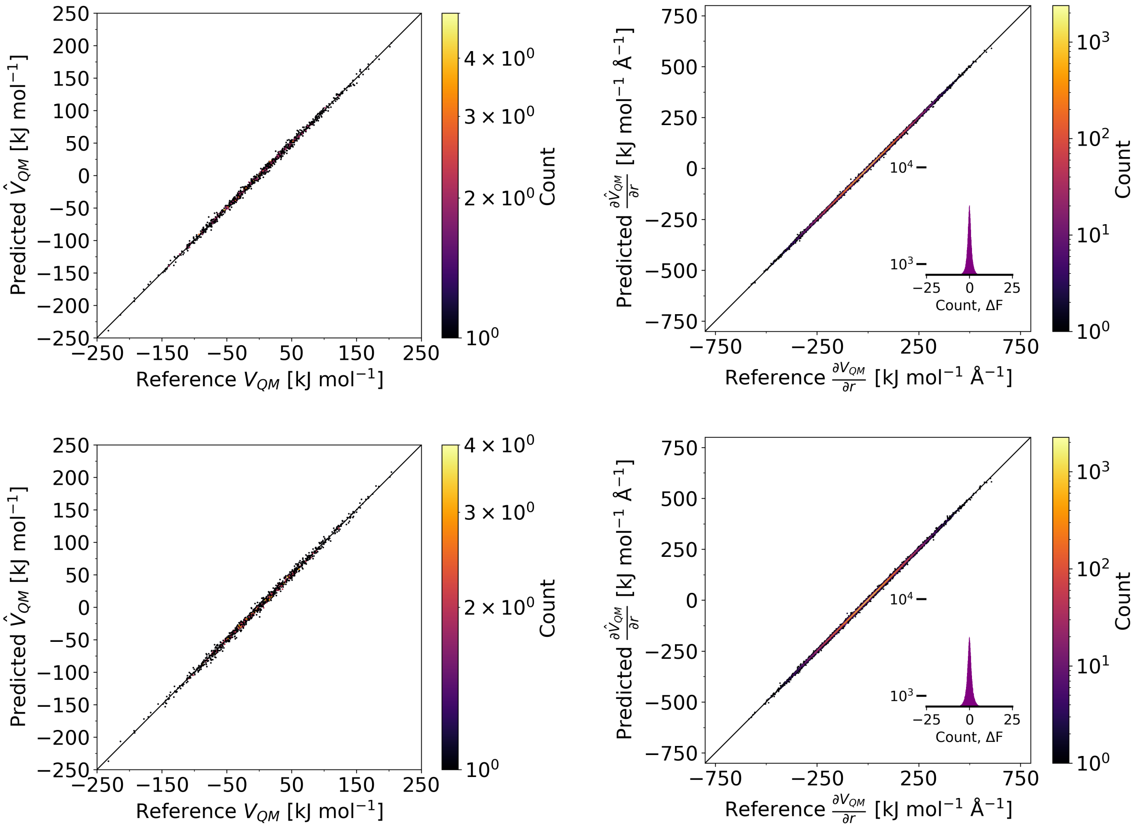

Figure S16: Correlation of predicted QM energies and gradients and their respective references using AMP. Top to bottom: entries 1–4 from Table 4 (see main text).

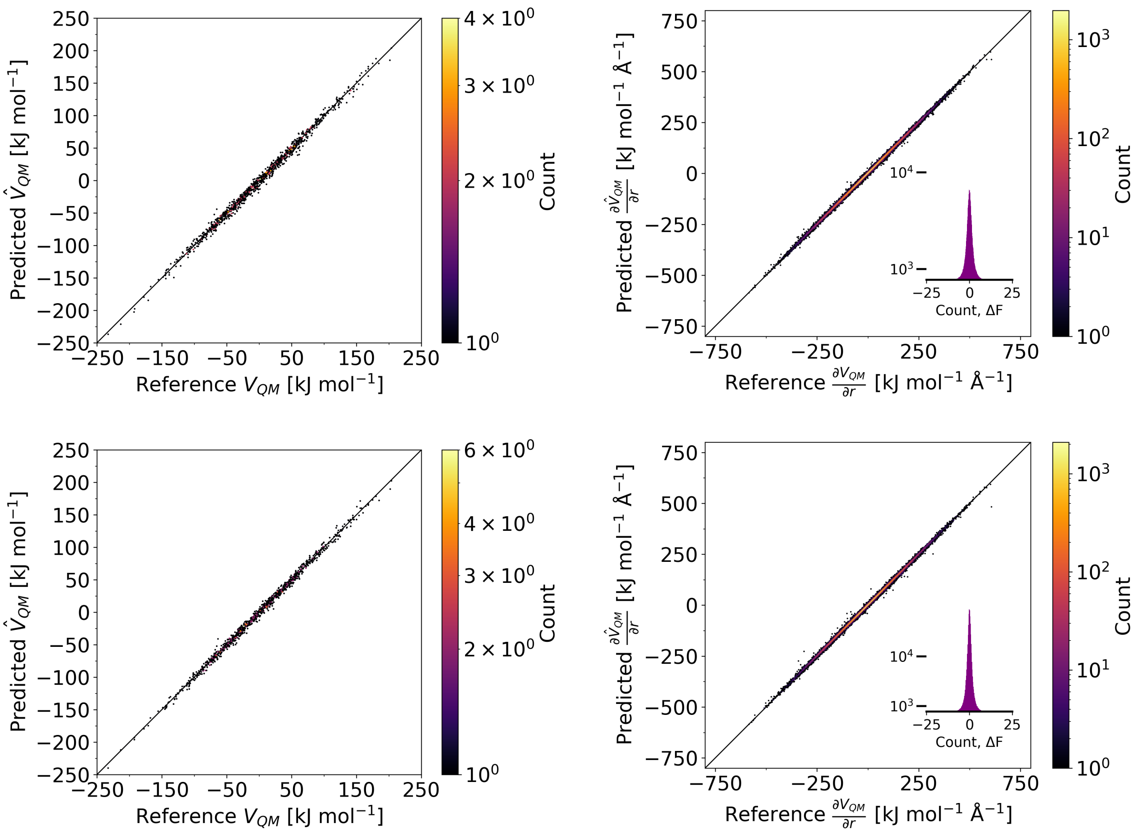

Figure S16: (cont.) Correlation of predicted QM energies and gradients and their respective references using AMP. Top to bottom: entries 1–4 from Table 4 (see main text).

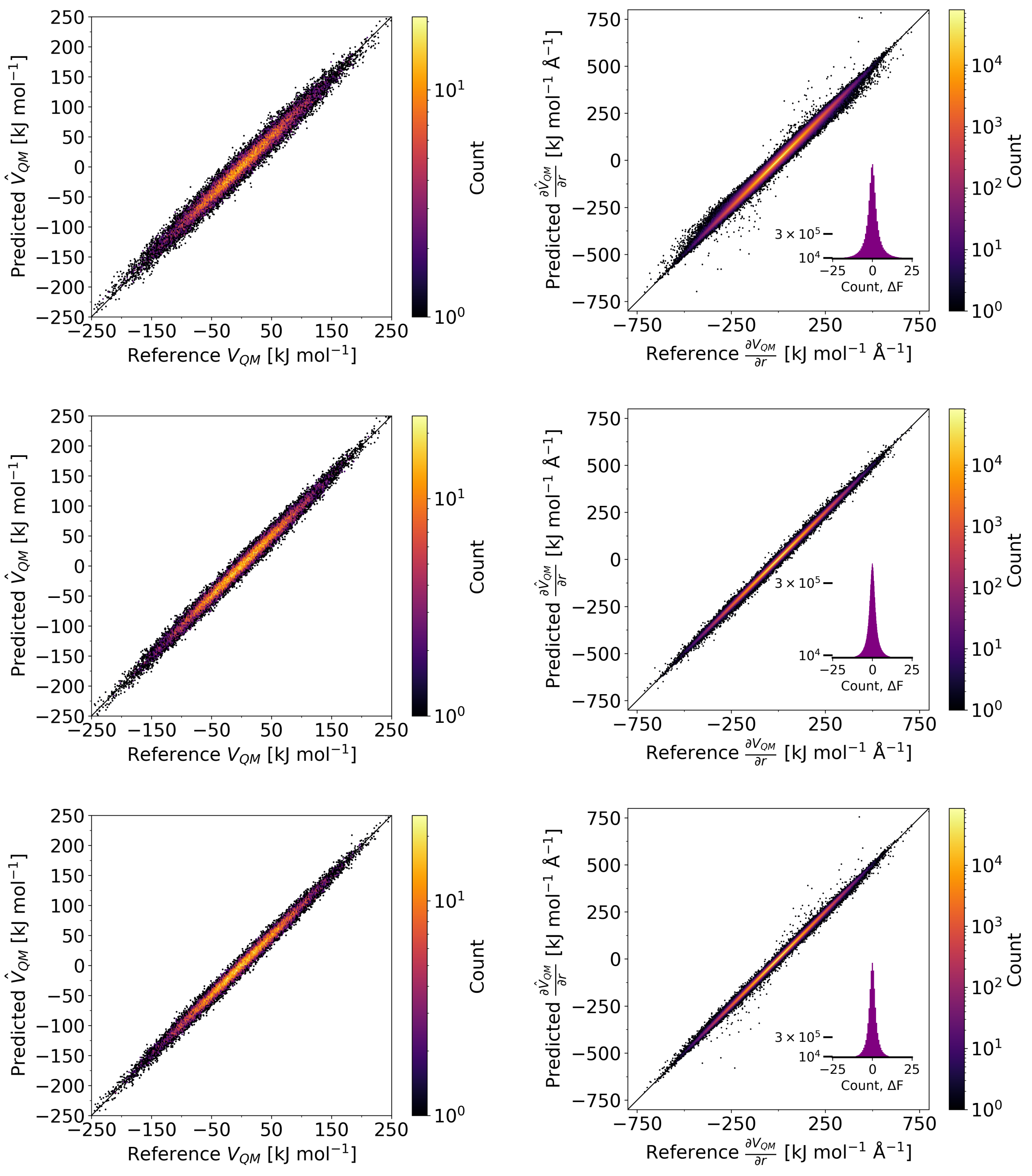

Figure S17: Correlation of predicted QM energies and gradients and their respective references using AMP. Top to bottom: entries 5–9 from Table 4 (see main text).

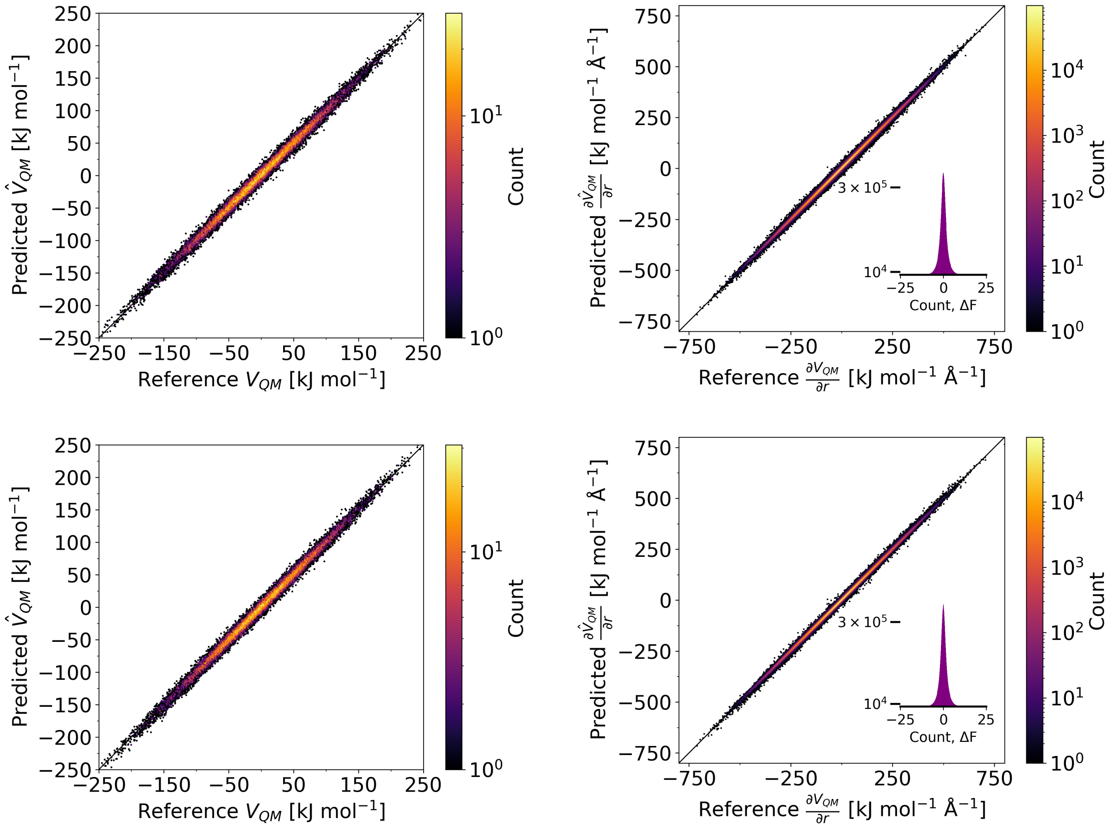

Figure S17: (cont.) Correlation of predicted QM energies and gradients and their respective references using AMP. Top to bottom: entries 5–9 from Table 4 (see main text).

Table S47: Statistical descriptors for QM energy predictions on the respective test set by models trained according to entries 1–4 from Table 4 (see main text). Values related to energy are reported in kJ mol^{-1}. Percentage Chemical Accuracy and Twice Percentage Chemical Accuracy refer to percentage of predictions within 4.184 and 8.368 kJ mol^{-1}, respectively.

	1	2	3	4
Mean Absolute Error	2.31	2.76	3.88	3.10
Median Absolute Error	1.81	2.25	3.03	2.50
Quantile 25	0.823	1.01	1.53	1.13
Quantile 75	3.29	3.93	5.38	4.36
RMSD	2.83	3.34	4.73	3.79
Max Unsigned Error	13.3	15.1	22.1	22.4
Max Signed Error	10.7	15.1	22.1	17.1
Min Signed Error	-13.3	-12.1	-20.6	-22.4
Spearman ρ	0.999	0.998	0.997	0.998
Kendall τ	0.970	0.965	0.953	0.961
Spearman P-Value	0	0	0	0
Kendall τ P-Value	0	0	0	0
Perc. Chem. Acc.	0.851	0.779	0.639	0.728
Twice Perc. Chem. Acc.	0.986	0.969	0.903	0.965

Table S48: Statistical descriptors for QM energy predictions on the respective test set by models trained according to entries 5–9 from Table 4 (see main text). Values related to energy are reported in kJ mol^{-1}. Percentage Chemical Accuracy and Twice Percentage Chemical Accuracy refer to percentage of predictions within 4.184 and 8.368 kJ mol^{-1}, respectively.

	5	6	7	8	9
Mean Absolute Error	7.62	5.68	4.95	4.31	4.16
Median Absolute Error	6.14	4.68	4.11	3.58	3.39
Quantile 25	2.85	2.18	1.94	1.67	1.60
Quantile 75	10.9	8.13	7.07	6.16	5.98
RMSD	9.16	6.76	5.87	5.13	4.97
Max Unsigned Error	51.1	31.6	32.7	27.4	26.6
Max Signed Error	49.5	31.6	27.6	27.4	26.6
Min Signed Error	-51.1	-30.4	-32.7	-26.8	-25.3
Spearman ρ	0.989	0.994	0.995	0.996	0.997
Kendall τ	0.909	0.933	0.941	0.949	0.951
Spearman P-Value	0	0	0	0	0
Kendall τ P-Value	0	0	0	0	0
Perc. Chem. Acc.	0.357	0.453	0.508	0.569	0.589
Twice Perc. Chem. Acc.	0.631	0.763	0.823	0.878	0.886

Table S49: Statistical descriptors for QM gradient predictions on the respective test set by models trained according to entries 1–4 from Table 4 (see main text). Values related to gradients are reported in kJ mol^{-1} $Å^{-1}$.

	1	2	3	4
Mean Absolute Error	1.10	1.31	1.65	1.51
Median Absolute Error	0.792	0.928	1.17	1.04
Quantile 25	0.358	0.419	0.530	0.467
Quantile 75	1.49	1.76	2.20	2.00
RMSD	1.55	1.84	2.36	2.25
Max Unsigned Error	31.9	37.8	60.0	124
Max Signed Error	24.6	28.0	48.1	124
Min Signed Error	-31.9	-37.8	-60.0	-87.5
Spearman ρ	1.00	1.00	1.00	1.00
Kendall τ	0.990	0.988	0.985	0.986
Spearman P-Value	0	0	0	0
Kendall τ P-Value	0	0	0	0

Table S50: Statistical descriptors for QM gradient predictions on the respective test set by models trained according to entries 5–9 from Table 4 (see main text). Values related to gradients are reported in kJ mol^{-1} $Å^{-1}$.

	5	6	7	8	9
Mean Absolute Error	3.70	2.37	2.24	1.73	1.69
Median Absolute Error	2.19	1.59	1.54	1.20	1.18
Quantile 25	0.940	0.708	0.692	0.543	0.535
Quantile 75	4.62	3.13	2.99	2.31	2.26
RMSD	5.93	3.46	3.24	2.50	2.41
Max Unsigned Error	414	95.4	350	74.9	74.9
Max Signed Error	414	89.9	350	56.7	59.2
Min Signed Error	-328	-95.4	-324	-74.9	-74.9
Spearman ρ	0.998	0.999	0.999	1.00	1.00
Kendall τ	0.966	0.978	0.980	0.984	0.985
Spearman P-Value	0	0	0	0	0
Kendall τ P-Value	0	0	0	0	0

Table S51: Statistical descriptors for MM gradient predictions on the respective test set by models trained according to entries 1–4 from Table 4 (see main text). Values related to gradients are reported in kJ mol^{-1} Å^{-1}.

	1	2	3	4
Mean Absolute Error	0.0139	0.015	0.0166	0.0175
Median Absolute Error	0.0045	0.00484	0.00520	0.00555
Quantile 25	0.00181	0.00197	0.00210	0.00224
Quantile 75	0.0113	0.0121	0.0130	0.0138
RMSD	0.0338	0.0360	0.0407	0.0436
Max Unsigned Error	2.66	2.36	4.26	7.17
Max Signed Error	2.52	2.31	4.26	4.04
Min Signed Error	-2.66	-2.36	-2.98	-7.17
Spearman ρ	0.999	0.998	0.998	0.998
Kendall τ	0.982	0.980	0.979	0.978
Spearman P-Value	0	0	0	0
Kendall τ P-Value	0	0	0	0

Table S52: Statistical descriptors for MM gradient predictions on the respective test set by models trained according to entries 5–9 from Table 4 (see main text). Values related to gradients are reported in kJ mol^{-1} Å^{-1}.

	5	6	7	8	9
Mean Absolute Error	0.0297	0.0196	0.0185	0.0173	0.0168
Median Absolute Error	0.0111	0.00664	0.00621	0.00561	0.00528
Quantile 25	0.00437	0.00270	0.00253	0.00229	0.00215
Quantile 75	0.0270	0.0162	0.0152	0.0139	0.0132
RMSD	0.0642	0.0450	0.0428	0.0406	0.0395
Max Unsigned Error	4.50	8.58	4.23	5.16	4.10
Max Signed Error	3.94	8.58	4.23	5.16	4.10
Min Signed Error	-4.50	-6.64	-3.47	-3.80	-3.82
Spearman ρ	0.995	0.997	0.998	0.998	0.998
Kendall τ	0.960	0.974	0.976	0.977	0.978
Spearman P-Value	0	0	0	0	0
Kendall τ P-Value	0	0	0	0	0

Table S53: Statistical descriptors for QM dipole predictions on the respective test set by models trained according to entries 1–4 from Table 4 (see main text). Values related to QM dipoles are reported in eÅ.

	1	2	3	4
Mean Absolute Error	0.0822	0.0877	0.0947	0.107
Median Absolute Error	0.0613	0.0670	0.0712	0.0815
Quantile 25	0.0289	0.0310	0.0329	0.0361
Quantile 75	0.113	0.122	0.132	0.146
RMSD	0.112	0.119	0.129	0.146
Max Unsigned Error	0.674	0.630	0.663	0.915
Max Signed Error	0.674	0.630	0.663	0.854
Min Signed Error	-0.554	-0.499	-0.658	-0.915
Spearman ρ	1.00	1.00	0.999	0.999
Kendall τ	0.983	0.981	0.980	0.978
Spearman P-Value	0	0	0	0
Kendall τ P-Value	0	0	0	0

Table S54: Statistical descriptors for QM dipole predictions on the respective test set by models trained according to entries 5–9 from Table 4 (see main text). Values related to QM dipoles are reported in eÅ.

	5	6	7	8	9
Mean Absolute Error	0.226	0.139	0.128	0.111	0.100
Median Absolute Error	0.165	0.106	0.0991	0.0876	0.0799
Quantile 25	0.0726	0.0486	0.0457	0.0402	0.0374
Quantile 75	0.316	0.193	0.178	0.155	0.141
RMSD	0.308	0.187	0.171	0.147	0.130
Max Unsigned Error	2.06	1.49	1.31	1.06	0.824
Max Signed Error	2.06	1.49	1.31	1.03	0.824
Min Signed Error	-1.52	-1.21	-1.07	-1.06	-0.764
Spearman ρ	0.996	0.998	0.999	0.999	0.999
Kendall τ	0.950	0.966	0.970	0.973	0.975
Spearman P-Value	0	0	0	0	0
Kendall τ P-Value	0	0	0	0	0

Table S55: Statistical descriptors for QM quadrupole predictions on the respective test set by models trained according to entries 1–4 from Table 4 (see main text). Values related to QM quadrupoles are reported in eÅ^2.

	1	2	3	4
Mean Absolute Error	0.380	0.400	0.425	0.461
Median Absolute Error	0.246	0.260	0.280	0.299
Quantile 25	0.0995	0.109	0.118	0.127
Quantile 75	0.511	0.544	0.578	0.619
RMSD	0.562	0.585	0.618	0.675
Max Unsigned Error	3.85	4.11	5.62	5.42
Max Signed Error	3.85	4.11	5.62	5.42
Min Signed Error	-3.29	-3.48	-3.93	-4.65
Spearman ρ	0.999	0.999	0.999	0.999
Kendall τ	0.978	0.977	0.975	0.973
Spearman P-Value	0	0	0	0
Kendall τ P-Value	0	0	0	0

Table S56: Statistical descriptors for QM quadrupole predictions on the respective test set by models trained according to entries 5–9 from Table 4 (see main text). Values related to QM quadrupoles are reported in eÅ^2.

	5	6	7	8	9
Mean Absolute Error	1.23	0.655	0.555	0.471	0.441
Median Absolute Error	0.815	0.442	0.382	0.329	0.307
Quantile 25	0.321	0.191	0.165	0.144	0.136
Quantile 75	1.71	0.891	0.758	0.641	0.597
RMSD	1.77	0.941	0.788	0.666	0.627
Max Unsigned Error	16.8	9.21	7.21	6.89	6.25
Max Signed Error	12.1	7.82	6.19	6.89	5.85
Min Signed Error	-16.8	-9.21	-7.21	-6.21	-6.25
Spearman ρ	0.985	0.996	0.997	0.998	0.998
Kendall τ	0.906	0.952	0.959	0.966	0.968
Spearman P-Value	0	0	0	0	0
Kendall τ P-Value	0	0	0	0	0

S1.8 Pyridine and Quinoline Dimers – AMP Model (2.7 Million Parameters)

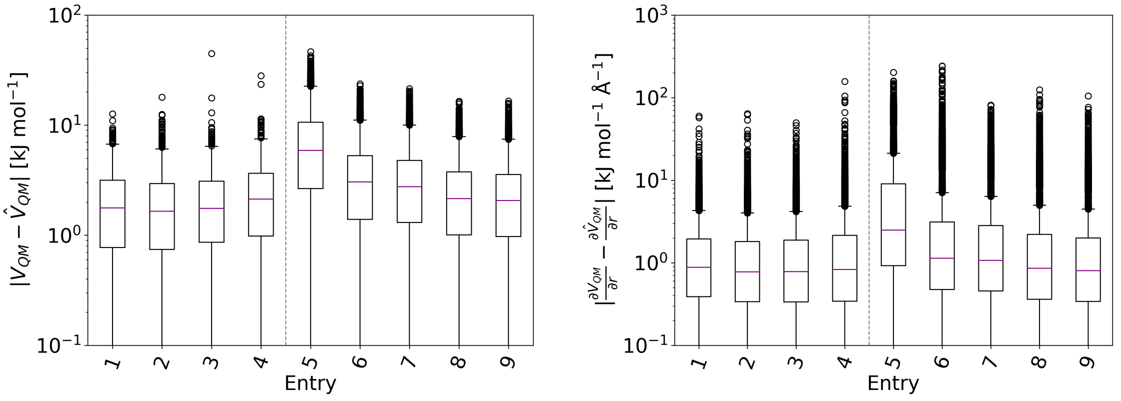

Figure S18: Distribution of the absolute errors of predicted QM energies and gradients of pyridine and quinoline dimers with respect to the reference for models trained on different training sets (see Table 3 in the main text for split definitions). All predictions were performed on the respective test sets.

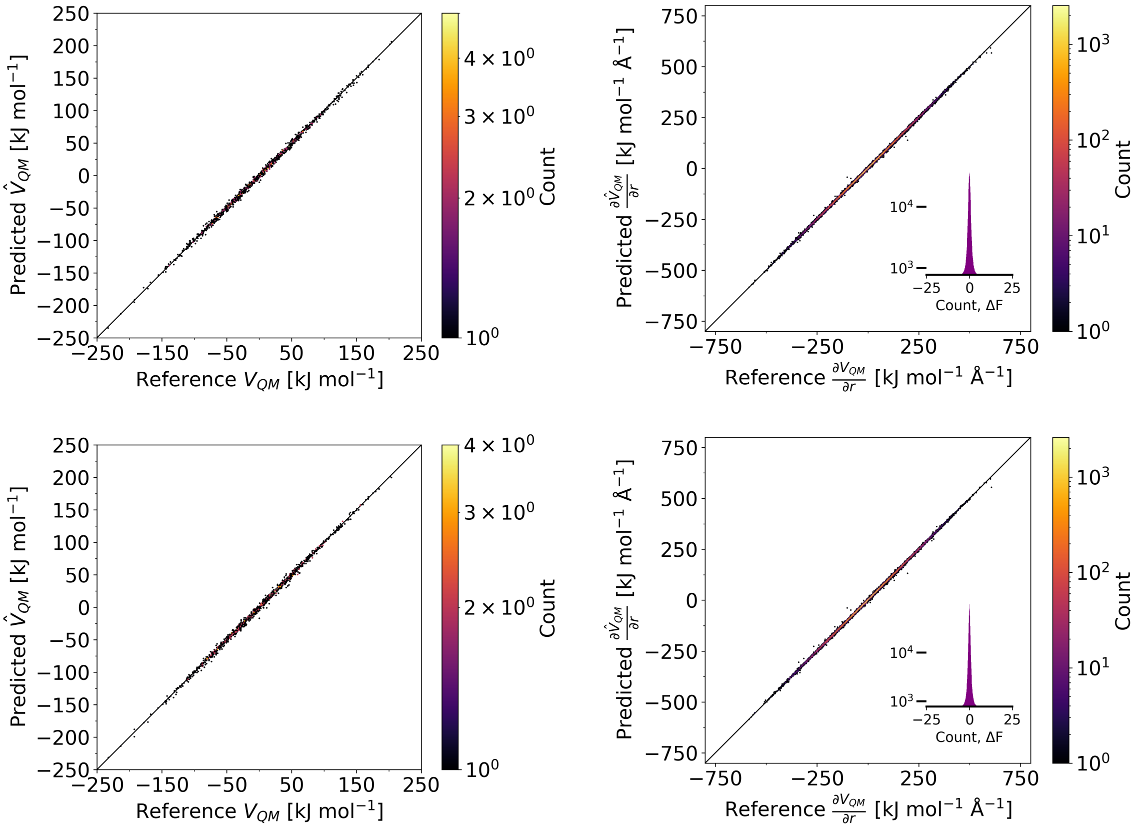

Figure S19: Correlation of predicted QM energies and gradients and their respective references using AMP. Top to bottom: entries 1–4 from Table 4 (see main text).

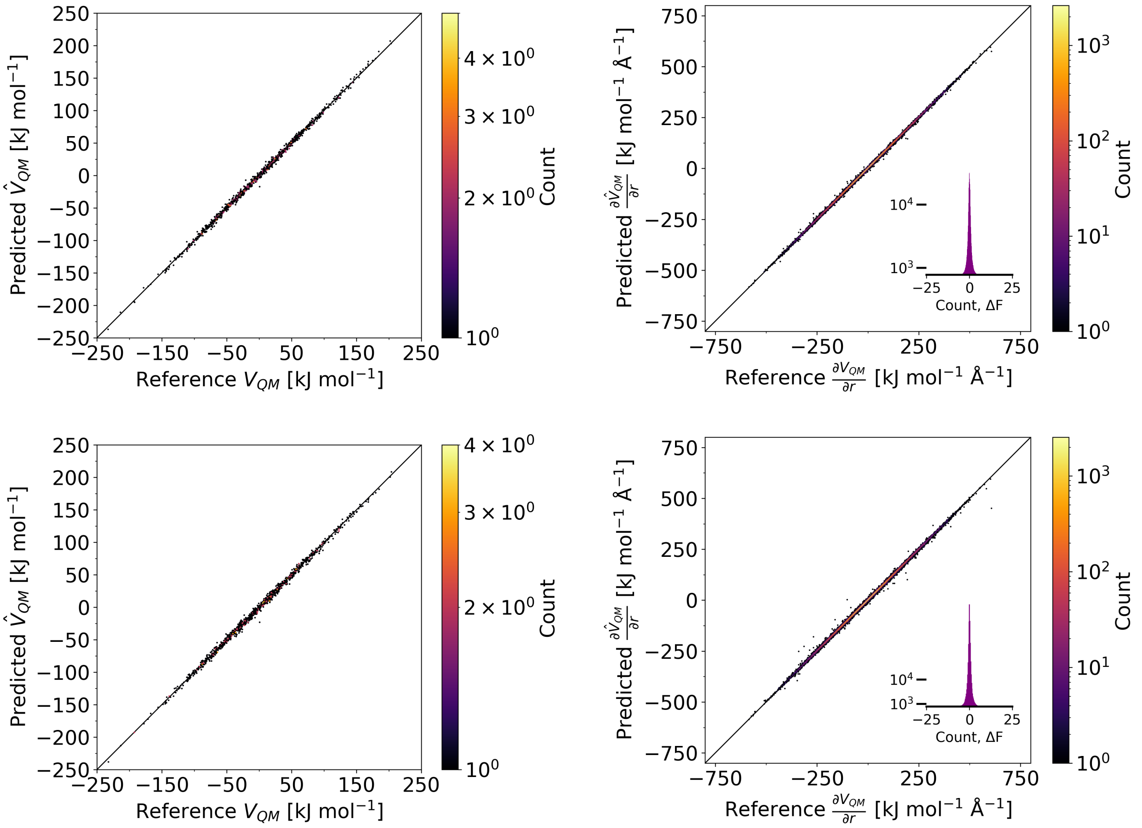

Figure S19: (cont.) Correlation of predicted QM energies and gradients and their respective references using AMP. Top to bottom: entries 1–4 from Table 4 (see main text).

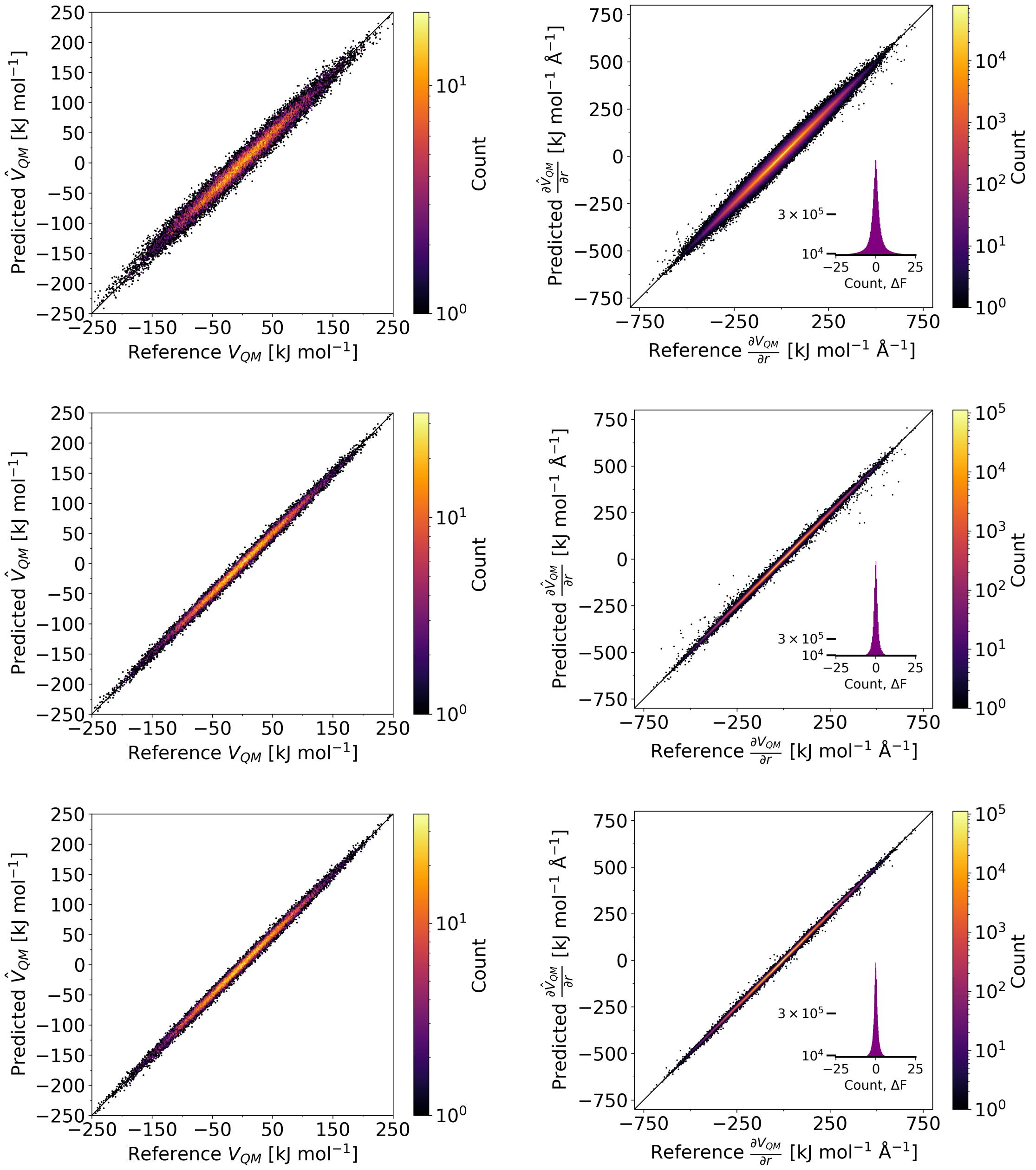

Figure S20: Correlation of predicted QM energies and gradients and their respective references using AMP. Top to bottom: entries 5–9 from Table 4 (see main text).

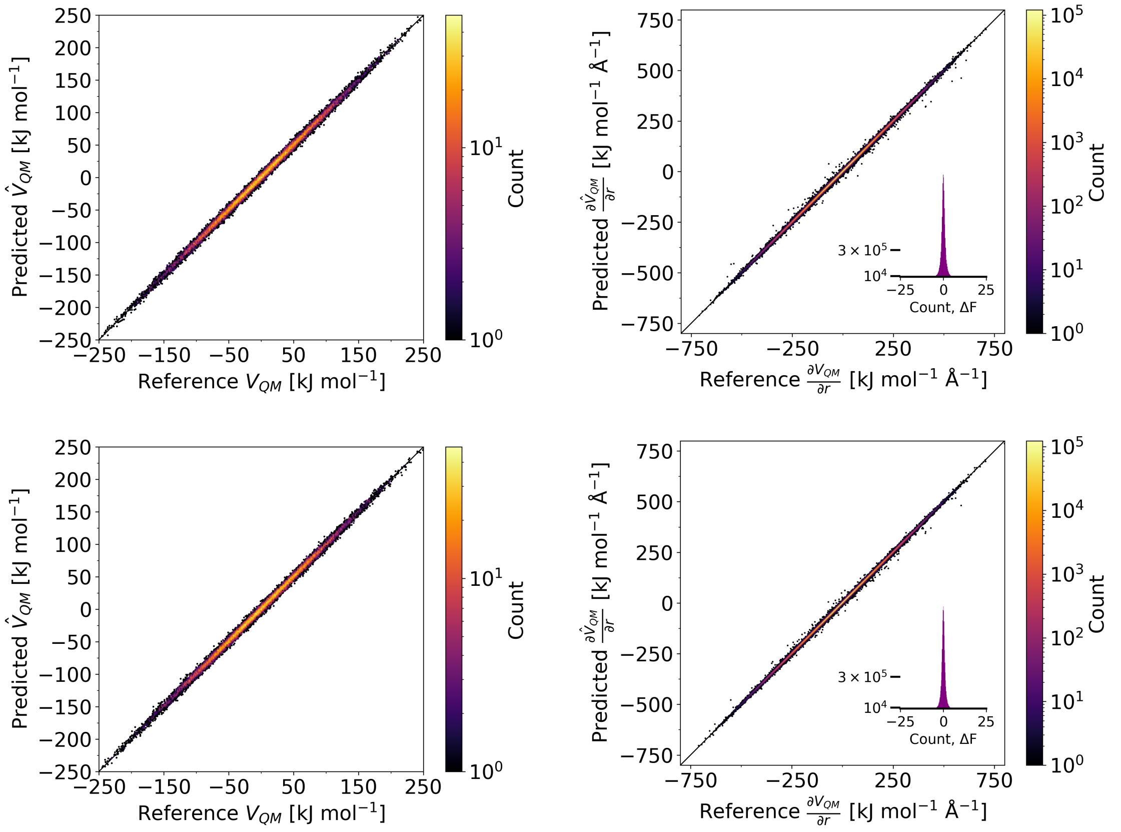

Figure S20: (cont.) Correlation of predicted QM energies and gradients and their respective references using AMP. Top to bottom: entries 5–9 from Table 4 (see main text).

Table S57: Statistical descriptors for QM energy predictions on the respective test set by models trained according to entries 1–4 from Table 4 (see main text). Values related to energy are reported in kJ mol^{-1}. Percentage Chemical Accuracy and Twice Percentage Chemical Accuracy refer to percentage of predictions within 4.184 and 8.368 kJ mol^{-1}, respectively.

	1	2	3	4
Mean Absolute Error	2.23	2.15	2.26	2.61
Median Absolute Error	1.77	1.65	1.76	2.13
Quantile 25	0.772	0.743	0.861	0.982
Quantile 75	3.15	2.94	3.09	3.65
RMSD	2.71	2.72	2.99	3.21
Max Unsigned Error	12.7	18.0	44.8	28.1
Max Signed Error	11.0	18.0	17.7	23.6
Min Signed Error	-12.7	-10.6	-44.8	-28.1
Spearman ρ	0.999	0.999	0.999	0.998
Kendall τ	0.972	0.972	0.972	0.967
Spearman P-Value	0	0	0	0
Kendall τ P-Value	0	0	0	0
Perc. Chem. Acc.	0.854	0.876	0.858	0.810
Twice Perc. Chem. Acc.	0.993	0.987	0.991	0.984

Table S58: Statistical descriptors for QM energy predictions on the respective test set by models trained according to entries 5–9 from Table 4 (see main text). Values related to energy are reported in kJ mol^{-1}. Percentage Chemical Accuracy and Twice Percentage Chemical Accuracy refer to percentage of predictions within 4.184 and 8.368 kJ mol^{-1}, respectively.

	5	6	7	8	9
Mean Absolute Error	7.35	3.69	3.36	2.62	2.5
Median Absolute Error	5.89	3.05	2.76	2.15	2.06
Quantile 25	2.66	1.39	1.30	1.01	0.970
Quantile 75	10.6	5.28	4.79	3.76	3.57
RMSD	8.86	4.41	4.00	3.12	2.98
Max Unsigned Error	46.5	23.8	21.5	16.5	16.6
Max Signed Error	46.5	21.2	20.5	16.5	14.4
Min Signed Error	-42.8	-23.8	-21.5	-13.9	-16.6
Spearman ρ	0.989	0.997	0.998	0.999	0.999
Kendall τ	0.912	0.956	0.960	0.969	0.970
Spearman P-Value	0	0	0	0	0
Kendall τ P-Value	0	0	0	0	0
Perc. Chem. Acc.	0.376	0.641	0.686	0.797	0.822
Twice Perc. Chem. Acc.	0.648	0.923	0.947	0.985	0.988

Table S59: Statistical descriptors for QM gradient predictions on the respective test set by models trained according to entries 1–4 from Table 4 (see main text). Values related to gradients are reported in kJ mol^{-1} $Å^{-1}$.

	1	2	3	4
Mean Absolute Error	0.958	0.875	0.899	1.01
Median Absolute Error	0.699	0.613	0.605	0.634
Quantile 25	0.323	0.277	0.272	0.279
Quantile 75	1.29	1.17	1.18	1.29
RMSD	1.36	1.28	1.35	1.67
Max Unsigned Error	60.0	64.1	49.6	157
Max Signed Error	57.1	62.4	49.6	157
Min Signed Error	-60.0	-64.1	-45.9	-105
Spearman ρ	1.00	1.00	1.00	1.00
Kendall τ	0.991	0.992	0.992	0.991
Spearman P-Value	0	0	0	0
Kendall τ P-Value	0	0	0	0

Table S60: Statistical descriptors for QM gradient predictions on the respective test set by models trained according to entries 5–9 from Table 4 (see main text). Values related to gradients are reported in kJ mol^{-1} $Å^{-1}$.

	5	6	7	8	9
Mean Absolute Error	3.59	1.31	1.21	0.948	0.858
Median Absolute Error	1.80	0.855	0.808	0.652	0.600
Quantile 25	0.745	0.380	0.360	0.292	0.271
Quantile 75	4.07	1.70	1.60	1.26	1.15
RMSD	6.33	1.99	1.77	1.38	1.23
Max Unsigned Error	202	242	81.5	125	105
Max Signed Error	202	242	80.7	125	105
Min Signed Error	-149	-208	-81.5	-108	-76.4
Spearman ρ	0.998	1.00	1.00	1.00	1.00
Kendall τ	0.966	0.988	0.989	0.991	0.992
Spearman P-Value	0	0	0	0	0
Kendall τ P-Value	0	0	0	0	0

Table S61: Statistical descriptors for MM gradient predictions on the respective test set by models trained according to entries 1–4 from Table 4 (see main text). Values related to gradients are reported in kJ mol^{-1} Å^{-1}.

	1	2	3	4
Mean Absolute Error	0.0126	0.0119	0.0120	0.0119
Median Absolute Error	0.00441	0.00432	0.0042	0.00437
Quantile 25	0.00178	0.00174	0.00171	0.00180
Quantile 75	0.0110	0.0107	0.0102	0.0103
RMSD	0.0288	0.0273	0.0299	0.0297
Max Unsigned Error	3.01	2.91	4.62	4.63
Max Signed Error	3.01	2.24	4.62	3.27
Min Signed Error	-2.76	-2.91	-4.09	-4.63
Spearman ρ	0.999	0.999	0.999	0.999
Kendall τ	0.984	0.985	0.985	0.985
Spearman P-Value	0	0	0	0
Kendall τ P-Value	0	0	0	0

Table S62: Statistical descriptors for MM gradient predictions on the respective test set by models trained according to entries 5–9 from Table 4 (see main text). Values related to gradients are reported in kJ mol^{-1} Å^{-1}.

	5	6	7	8	9
Mean Absolute Error	0.0258	0.0139	0.0137	0.0124	0.0121
Median Absolute Error	0.00923	0.00506	0.00477	0.00443	0.00440
Quantile 25	0.00357	0.00207	0.00194	0.00181	0.00180
Quantile 75	0.0229	0.0122	0.0117	0.0109	0.0107
RMSD	0.0566	0.0304	0.0309	0.0272	0.0267
Max Unsigned Error	4.28	4.55	3.40	3.96	3.96
Max Signed Error	4.28	2.85	2.67	3.96	3.96
Min Signed Error	-3.31	-4.55	-3.40	-2.65	-2.74
Spearman ρ	0.996	0.999	0.999	0.999	0.999
Kendall τ	0.965	0.981	0.982	0.983	0.984
Spearman P-Value	0	0	0	0	0
Kendall τ P-Value	0	0	0	0	0

Table S63: Statistical descriptors for QM dipole predictions on the respective test set by models trained according to entries 1–4 from Table 4 (see main text). Values related to QM dipoles are reported in eÅ.

	1	2	3	4
Mean Absolute Error	0.0780	0.0751	0.0755	0.0836
Median Absolute Error	0.0579	0.0569	0.0585	0.0634
Quantile 25	0.0271	0.0269	0.0277	0.0305
Quantile 75	0.108	0.102	0.104	0.115
RMSD	0.106	0.102	0.101	0.113
Max Unsigned Error	0.751	0.658	0.766	0.770
Max Signed Error	0.751	0.658	0.678	0.770
Min Signed Error	-0.546	-0.470	-0.766	-0.738
Spearman ρ	1.00	1.00	1.00	1.00
Kendall τ	0.984	0.984	0.984	0.982
Spearman P-Value	0	0	0	0
Kendall τ P-Value	0	0	0	0

Table S64: Statistical descriptors for QM dipole predictions on the respective test set by models trained according to entries 5–9 from Table 4 (see main text). Values related to QM dipoles are reported in eÅ.

	5	6	7	8	9
Mean Absolute Error	0.188	0.105	0.0951	0.0869	0.0849
Median Absolute Error	0.134	0.0812	0.0749	0.0690	0.0678
Quantile 25	0.0589	0.0373	0.0349	0.0324	0.0316
Quantile 75	0.263	0.145	0.132	0.122	0.119
RMSD	0.259	0.139	0.125	0.113	0.111
Max Unsigned Error	2.02	0.987	0.959	0.785	0.819
Max Signed Error	2.02	0.987	0.959	0.785	0.819
Min Signed Error	-1.39	-0.828	-0.916	-0.669	-0.779
Spearman ρ	0.997	0.999	0.999	0.999	0.999
Kendall τ	0.954	0.975	0.977	0.979	0.979
Spearman P-Value	0	0	0	0	0
Kendall τ P-Value	0	0	0	0	0

Table S65: Statistical descriptors for QM quadrupole predictions on the respective test set by models trained according to entries 1–4 from Table 4 (see main text). Values related to QM quadrupoles are reported in eÅ^2.

	1	2	3	4
Mean Absolute Error	0.370	0.375	0.389	0.412
Median Absolute Error	0.235	0.242	0.247	0.270
Quantile 25	0.0949	0.102	0.106	0.112
Quantile 75	0.499	0.506	0.520	0.554
RMSD	0.552	0.556	0.577	0.607
Max Unsigned Error	4.04	4.73	4.55	5.15
Max Signed Error	3.89	4.73	4.55	4.54
Min Signed Error	-4.04	-4.60	-4.15	-5.15
Spearman ρ	0.999	0.999	0.999	0.999
Kendall τ	0.979	0.978	0.977	0.976
Spearman P-Value	0	0	0	0
Kendall τ P-Value	0	0	0	0

Table S66: Statistical descriptors for QM quadrupole predictions on the respective test set by models trained according to entries 5–9 from Table 4 (see main text). Values related to QM quadrupoles are reported in eÅ^2.

	5	6	7	8	9
Mean Absolute Error	1.19	0.509	0.466	0.401	0.384
Median Absolute Error	0.736	0.349	0.320	0.276	0.265
Quantile 25	0.280	0.151	0.138	0.121	0.117
Quantile 75	1.60	0.691	0.635	0.543	0.523
RMSD	1.80	0.727	0.665	0.571	0.547
Max Unsigned Error	18.9	7.46	6.84	5.22	5.96
Max Signed Error	17.8	6.42	5.59	4.29	4.28
Min Signed Error	-18.9	-7.46	-6.84	-5.22	-5.96
Spearman ρ	0.984	0.998	0.998	0.999	0.999
Kendall τ	0.908	0.963	0.966	0.971	0.972
Spearman P-Value	0	0	0	0	0
Kendall τ P-Value	0	0	0	0	0

S1.9 Pyridine and Quinoline Dimers – GFN2-xTB

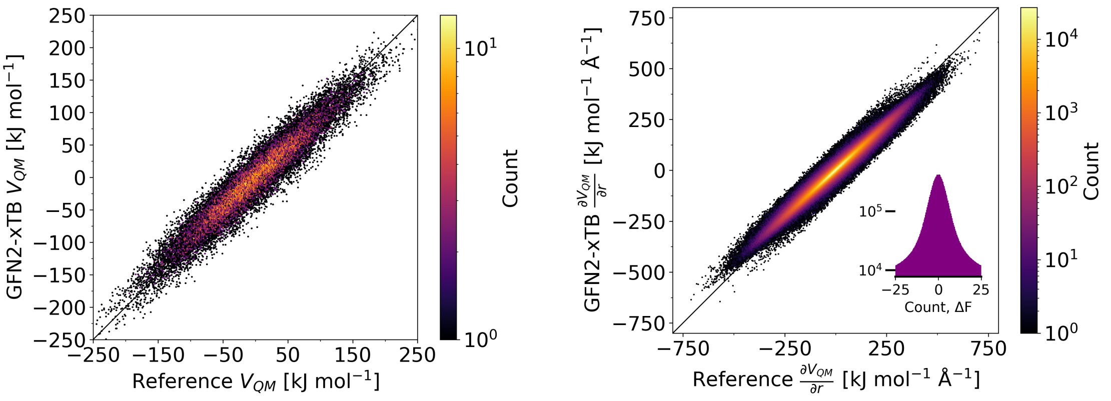

Figure S21: Correlation plots of GFN2-xTB QM energies and gradients vs their respective QM values.

Table S67: Statistical descriptors for QM energy predictions of GFN2-xTB. Values related to energy are reported in kJ mol^{-1}. Percentage Chemical Accuracy and Twice Percentage Chemical Accuracy refer to percentage of predictions within 4.184 and 8.368 kJ mol^{-1}, respectively.

	GFN2-xTB
Mean Absolute Error	17.8
Median Absolute Error	13.2
Quantile 25	5.78
Quantile 75	24.0
RMSD	22.5
Max Unsigned Error	99.6
Max Signed Error	99.6
Min Signed Error	-90.9
Spearman ρ	0.928
Kendall τ	0.777
Spearman P-Value	0
Kendall τ P-Value	0
Perc. Chem. Acc.	0.177
Twice Perc. Chem. Acc.	0.351

Table S68: Statistical descriptors for QM gradient predictions of GFN2-xTB. Values related to gradients are reported in kJ mol^{-1} $Å^{-1}$.

	GFN2-xTB
Mean Absolute Error	11.7
Median Absolute Error	7.52
Quantile 25	3.32
Quantile 75	15.4
RMSD	17.2
Max Unsigned Error	159
Max Signed Error	141
Min Signed Error	-159
Spearman ρ	0.985
Kendall τ	0.906
Spearman P-Value	0
Kendall τ P-Value	0
Perc. Chem. Acc.	0.309
Twice Perc. Chem. Acc.	0.539

Table S69: Statistical descriptors for MM gradient predictions of GFN2-xTB. Values related to gradients are reported in kJ mol^{-1} $Å^{-1}$.

	GFN2-xTB
Mean Absolute Error	0.0575
Median Absolute Error	0.0148
Quantile 25	0.00582
Quantile 75	0.0395
RMSD	0.155
Max Unsigned Error	11.9
Max Signed Error	11.9
Min Signed Error	-10.4
Spearman ρ	0.981
Kendall τ	0.932
Spearman P-Value	0
Kendall τ P-Value	0

S2 Umbrella Definitions

S2.1 Alanine Dipeptide

Table S70: Umbrella definition for alanine dipeptide free energy calculations. Dihedral minima ϕ_0 and ψ_0 in degrees and force constant k in kJ mol^{-1} deg^{-2}.

ϕ_0	ψ_0	k
15.00	15.00	0.03
15.00	51.67	0.03
15.00	88.33	0.03
15.00	125.00	0.03
15.00	161.67	0.03
15.00	-161.67	0.03
15.00	-125.00	0.03
15.00	-88.33	0.03
15.00	-51.67	0.03
15.00	-15.00	0.03
51.67	15.00	0.03
51.67	51.67	0.03
51.67	88.33	0.03
51.67	125.00	0.03
51.67	161.67	0.03
51.67	-161.67	0.03
51.67	-125.00	0.03
51.67	-88.33	0.03
51.67	-51.67	0.03
51.67	-15.00	0.03
88.33	15.00	0.03
88.33	51.67	0.03
88.33	88.33	0.03
88.33	125.00	0.03
88.33	161.67	0.03
88.33	-161.67	0.03
88.33	-125.00	0.03
88.33	-88.33	0.03
88.33	-51.67	0.03
88.33	-15.00	0.03
125.00	15.00	0.03
125.00	51.67	0.03
125.00	88.33	0.03
125.00	125.00	0.03
125.00	161.67	0.03
125.00	-161.67	0.03
125.00	-125.00	0.03
125.00	-88.33	0.03

ϕ_0	ψ_0	k
125.00	-51.67	0.03
125.00	-15.00	0.03
161.67	15.00	0.03
161.67	51.67	0.03
161.67	88.33	0.03
161.67	125.00	0.03
161.67	161.67	0.03
161.67	-161.67	0.03
161.67	-125.00	0.03
161.67	-88.33	0.03
161.67	-51.67	0.03
161.67	-15.00	0.03
-161.67	15.00	0.03
-161.67	51.67	0.03
-161.67	88.33	0.03
-161.67	125.00	0.03
-161.67	161.67	0.03
-161.67	-161.67	0.03
-161.67	-125.00	0.03
-161.67	-88.33	0.03
-161.67	-51.67	0.03
-161.67	-15.00	0.03
-125.00	15.00	0.03
-125.00	51.67	0.03
-125.00	88.33	0.03
-125.00	125.00	0.03
-125.00	161.67	0.03
-125.00	-161.67	0.03
-125.00	-125.00	0.03
-125.00	-88.33	0.03
-125.00	-51.67	0.03
-125.00	-15.00	0.03
-88.33	15.00	0.03
-88.33	51.67	0.03
-88.33	88.33	0.03
-88.33	125.00	0.03
-88.33	161.67	0.03
-88.33	-161.67	0.03
-88.33	-125.00	0.03
-88.33	-88.33	0.03
-88.33	-51.67	0.03
-88.33	-15.00	0.03
-51.67	15.00	0.03
-51.67	51.67	0.03
-51.67	88.33	0.03

ϕ_0	ψ_0	k
-51.67	125.00	0.03
-51.67	161.67	0.03
-51.67	-161.67	0.03
-51.67	-125.00	0.03
-51.67	-88.33	0.03
-51.67	-51.67	0.03
-51.67	-15.00	0.03
-15.00	15.00	0.03
-15.00	51.67	0.03
-15.00	88.33	0.03
-15.00	125.00	0.03
-15.00	161.67	0.03
-15.00	-161.67	0.03
-15.00	-125.00	0.03
-15.00	-88.33	0.03
-15.00	-51.67	0.03
-15.00	-15.00	0.03

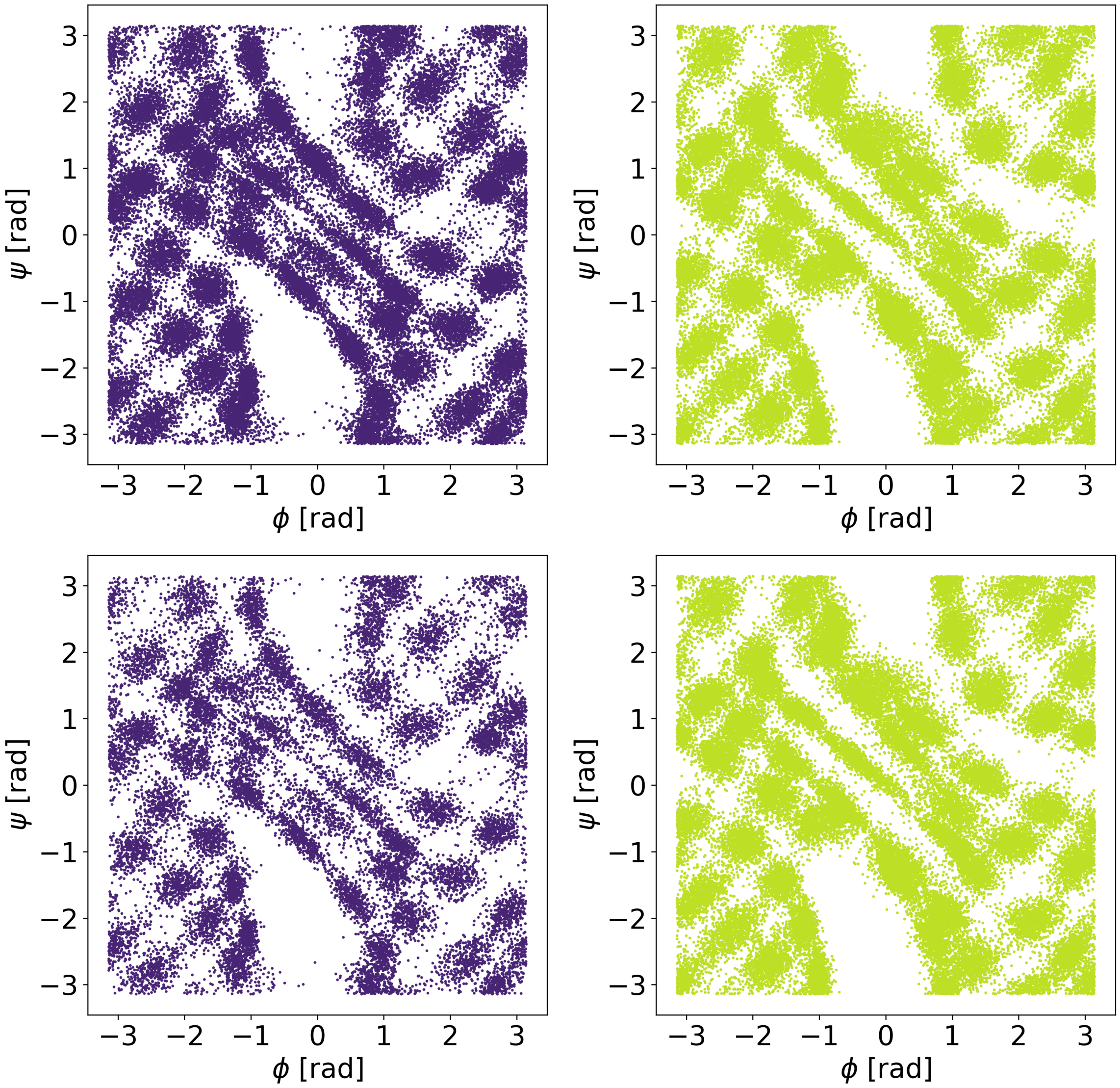

Figure S22: Visualization of checkerboard pattern of entries 6 and 7 according to Table 1 in the main text. Training (purple) and test data (yellow) shown for both entries.

S2.2 Nickel Phosphine Complexes

Table S71: Umbrella definition for nickel phosphine free energy calculations. Distance minimum r_0 in Å and force constant k in kJ mol^{-1} Å^{-2}.

r_0	k
2.0	20
2.5	20
3.0	20
3.5	20

r_0	k
4.0	20
4.5	20
5.0	20
5.5	20
6.0	20
6.5	20
7.0	20
7.5	20
8.0	20
8.5	20
9.0	20
9.5	20
10.0	20
10.5	20
11.0	20
11.5	20
12.0	20
12.5	20
13.0	20
13.5	20
14.0	20
14.5	20
15.0	20
15.5	20
16.0	20
16.5	20
17.0	20
17.5	20
18.0	20
18.5	20
19.0	20
19.5	20
20.0	20
2.75	100
3.25	100
3.75	100
4.25	100
4.75	100
5.25	100
5.75	100
6.25	100

S2.3 Pyridine and Quinoline Dimers

Table S72: Umbrella definition for pyridine and quinoline free energy calculations. Distance minimum r_0 in Å and force constant k in kJ mol^{-1} Å^{-2}.

r_0	k
2.0	20
2.5	20
3.0	20
3.5	20
4.0	20
4.5	20
5.0	20
5.5	20
6.0	20
6.5	20
7.0	20
7.5	20
8.0	20
8.5	20
9.0	20
9.5	20
10.0	20
10.5	20
11.0	20
11.5	20
12.0	20
12.5	20
13.0	20
13.5	20
14.0	20
14.5	20
15.0	20
15.5	20
16.0	20
16.5	20
17.0	20
17.5	20
18.0	20
18.5	20
19.0	20
19.5	20
20.0	20
2.7	100
3.3	100
3.7	100
4.3	100
4.7	100

S3 Free-Energy Calculations

S3.1 Alanine Dipeptide – AMP Model (600'000 Parameters)

Table S73: Torsional angles ϕ,ψ in radians and relative free energy ΔG in kJ mol^{-1} of local minima identified for alanine dipeptide with static QM calculations (B2-PLYP), QM/MM MD (GFN2-xTB), or ML/MM MD (AMP). AMP model entries correspond to those reported in Table 1 in the main text.

Method	Quantity	$C5$	P_{II}	$C7_{eq}$	α_R	α_L	$C7_{ax}$	α_D	α'_D	α'_R
B2-PLYP	ϕ	-2.73	-1.17	-1.49	-1.31	1.03	1.29	0.974	-	-
	ψ	2.75	2.50	1.25	-0.392	0.646	-0.848	-2.44	-	-
	ΔG	2.32	0.813	4.75	0.00	9.19	12.3	13.2	-	-
GFN2-xTB	ϕ	-2.32	-	-1.45	-1.07	0.817	1.19	0.817	-	-
	ψ	2.83	-	1.32	-0.691	0.817	-1.07	-2.20	-	-
	ΔG	2.63	-	0.00	6.67	11.2	6.26	11.8	-	-
AMP ϕ/ψ entry 1	ϕ	-	-1.19	-	-1.19	0.942	1.32	0.942	1.45	-2.07
	ψ	-	2.70	-	-0.565	0.817	-0.817	-2.45	2.70	-1.70
	ΔG	-	2.88	-	0.00	11.4	25.4	20.7	22.3	12.6
AMP ϕ/ψ entry 2	ϕ	-	-1.19	-	-1.32	0.942	1.32	0.942	1.32	-2.07
	ψ	-	2.70	-	-0.440	0.817	-0.817	-2.45	2.83	-1.70
	ΔG	-	2.54	-	0.00	10.2	24.0	19.0	20.0	12.4
AMP ϕ^-/ψ entry 8	ϕ	-	-1.19	-	-1.19	1.07	1.32	1.07	1.32	-2.07
	ψ	-	2.70	-	-0.565	0.691	-0.817	-2.45	2.83	-1.70
	ΔG	-	4.28	-	0.00	10.7	25.3	19.7	20.1	12.6
AMP ϕ^+/ψ entry 9	ϕ	-	-1.19	-	-1.19	0.942	1.32	0.942	1.45	-2.20
	ψ	-	2.70	-	-0.565	0.817	-0.817	-2.45	2.70	-1.70
	ΔG	-	1.86	-	0.00	9.25	22.4	17.5	19.4	9.20
AMP ϕ^-/ψ entry 10	ϕ	-	-1.19	-	-1.19	1.07	1.32	0.942	1.45	-2.07
	ψ	-	2.70	-	-0.565	0.565	-0.817	-2.45	2.70	-1.70
	ΔG	-	3.20	-	0.00	10.8	24.7	21.1	22.1	12.3
AMP ϕ^-/ψ^+ entry 11	ϕ	-	-1.19	-	-1.19	0.942	1.32	0.942	1.45	-1.95
	ψ	-	2.70	-	-0.565	0.817	-0.817	-2.45	2.45	-2.07
	ΔG	-	2.64	-	0.00	12.1	26.2	21.7	21.4	15.0

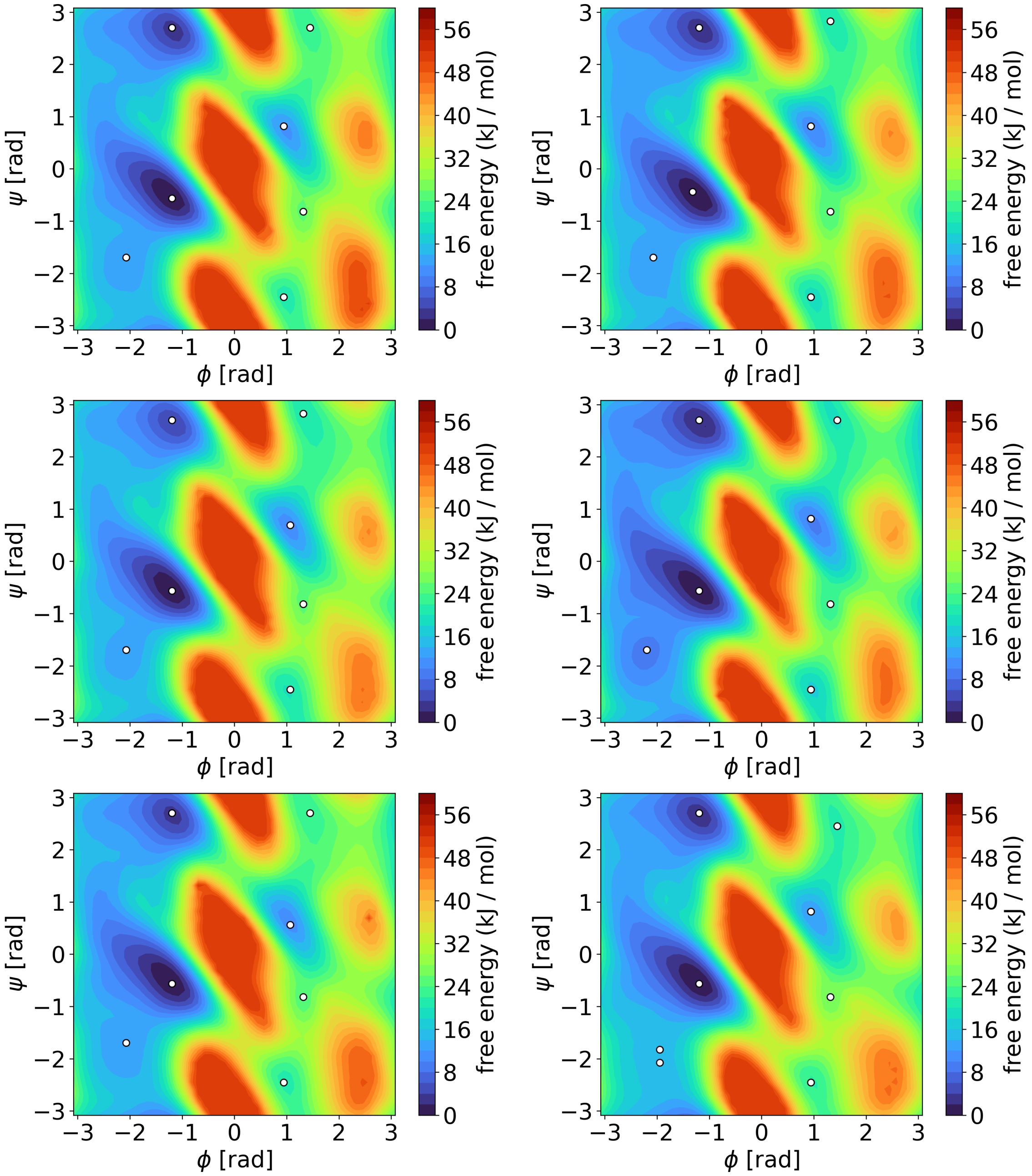

Figure S23: Free-energy landscape and local minima (white dots) calculated via umbrella sampling (600 ns overall sampling time) for alanine dipeptide **1** using the AMP Hamiltonian trained according to entries 1, 2, 8, 9, 10, 11 (top to bottom, left to right) according to Table 1 in the main text.

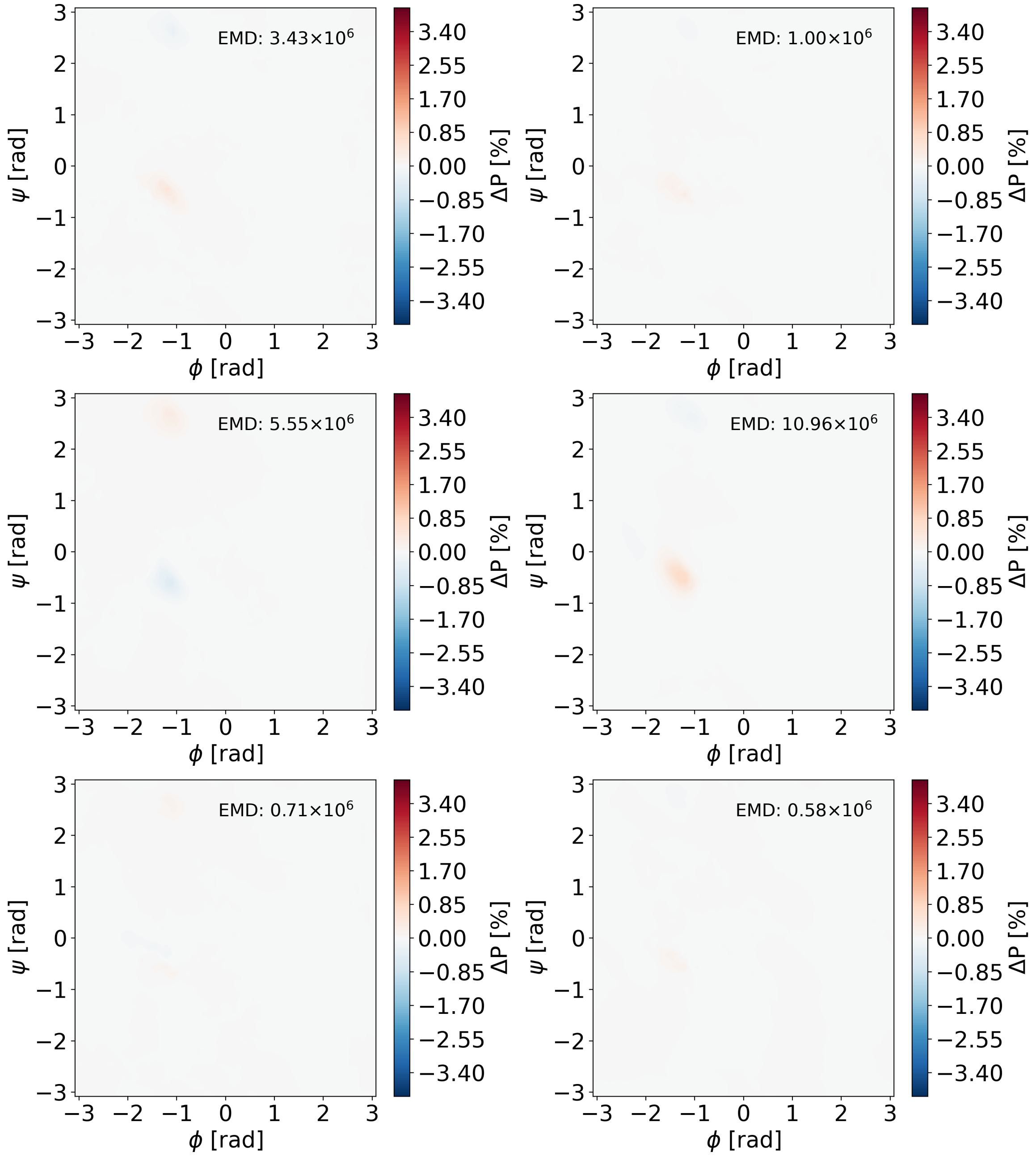

Figure S24: Difference in probability distribution and earth mover's distance (EMD) between two simulations. Difference between two simulations (200 ns overall sampling time each) using the AMP Hamiltonian trained according to entry 1 of Table 1 (top, left). Remaining entries (top to bottom, left to right): Difference between two simulations (600 ns overall sampling time each) using the AMP Hamiltonian trained according to entries 2, 8, 9, 10, and 11 to the one computed with the AMP Hamiltonian trained according to entry 1 of the same Table. EMD is a similarity measure between two probability distributions as a solution of the optimal transport problem.

S3.2 Alanine Dipeptide – AMP Model (2.7 Million Parameters)

Table S74: Torsional angles ϕ,ψ in radians and relative free energy ΔG in kJ mol^{-1} of local minima identified for alanine dipeptide with static QM calculations (B2-PLYP), QM/MM MD (GFN2-xTB), or ML/MM MD (AMP). AMP model entries correspond to those reported in Table 1 in the main text.

Method	Quantity	$C5$	P_{II}	$C7_{eq}$	α_R	α_L	$C7_{ax}$	α_D	α'_D	α'_R
B2-PLYP	ϕ	-2.73	-1.17	-1.49	-1.31	1.03	1.29	0.974	-	-
	ψ	2.75	2.50	1.25	-0.392	0.646	-0.848	-2.44	-	-
	ΔG	2.32	0.813	4.75	0.00	9.19	12.3	13.2	-	-
GFN2-xTB	ϕ	-2.32	-	-1.45	-1.07	0.817	1.19	0.817	-	-
	ψ	2.83	-	1.32	-0.691	0.817	-1.07	-2.20	-	-
	ΔG	2.63	-	0.00	6.67	11.2	6.26	11.8	-	-
AMP ϕ/ψ entry 1	ϕ	-	-1.19	-	-1.19	0.942	1.32	0.942	1.32	-2.07
	ψ	-	2.70	-	-0.565	0.817	-0.817	-2.45	2.83	-1.70
	ΔG	-	3.86	-	0.00	10.5	24.1	18.9	19.8	12.11
AMP ϕ/ψ entry 2	ϕ	-	-1.19	-	-1.19	0.942	1.32	0.942	1.32	-2.07
	ψ	-	2.70	-	-0.565	0.817	-0.817	-2.45	2.83	-1.70
	ΔG	-	3.30	-	0.00	10.4	23.8	19.0	20.2	12.3
AMP ϕ^-/ψ entry 8	ϕ	-	-1.19	-	-1.19	1.07	1.32	0.942	1.32	-2.07
	ψ	-	2.70	-	-0.565	0.691	-0.817	-2.45	2.95	-1.70
	ΔG	-	3.63	-	0.00	12.1	25.8	19.9	21.8	12.2
AMP ϕ^+/ψ entry 9	ϕ	-	-1.07	-	-1.19	0.942	1.32	0.942	1.32	-2.20
	ψ	-	2.58	-	-0.565	0.817	-0.817	-2.45	2.83	-1.82
	ΔG	-	0.486	-	0.00	7.06	21.0	16.7	17.9	9.62
AMP ϕ^-/ψ entry 10	ϕ	-	-1.19	-	-1.19	1.07	1.32	0.942	1.45	-2.07
	ψ	-	2.70	-	-0.565	0.691	-0.817	-2.45	2.58	-1.70
	ΔG	-	4.06	-	0.00	11.0	25.3	20.2	21.2	12.3
AMP ϕ^-/ψ^+ entry 11	ϕ	-	-1.19	-	-1.19	1.07	1.32	0.942	1.45	-1.95
	ψ	-	2.70	-	-0.565	0.691	-0.817	-2.45	2.58	-1.57
	ΔG	-	4.46	-	0.00	12.2	26.5	22.5	21.3	14.8

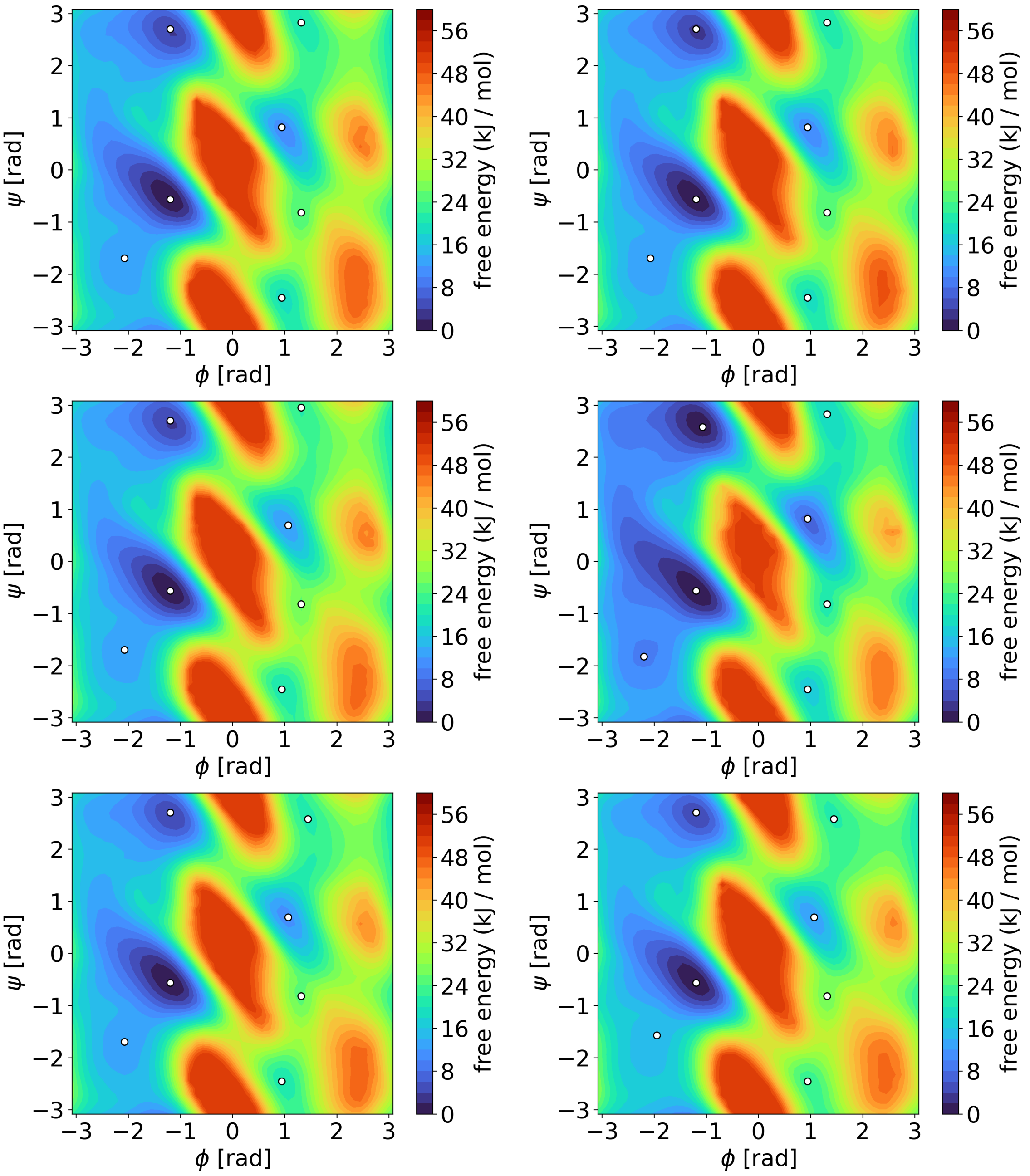

Figure S25: Free-energy landscape and local minima (white dots) calculated via umbrella sampling (600 ns overall sampling time) for alanine dipeptide **1** using the AMP Hamiltonian trained according to entries 1, 2, 8, 9, 10, 11 (top to bottom, left to right) according to Table 1 in the main text.

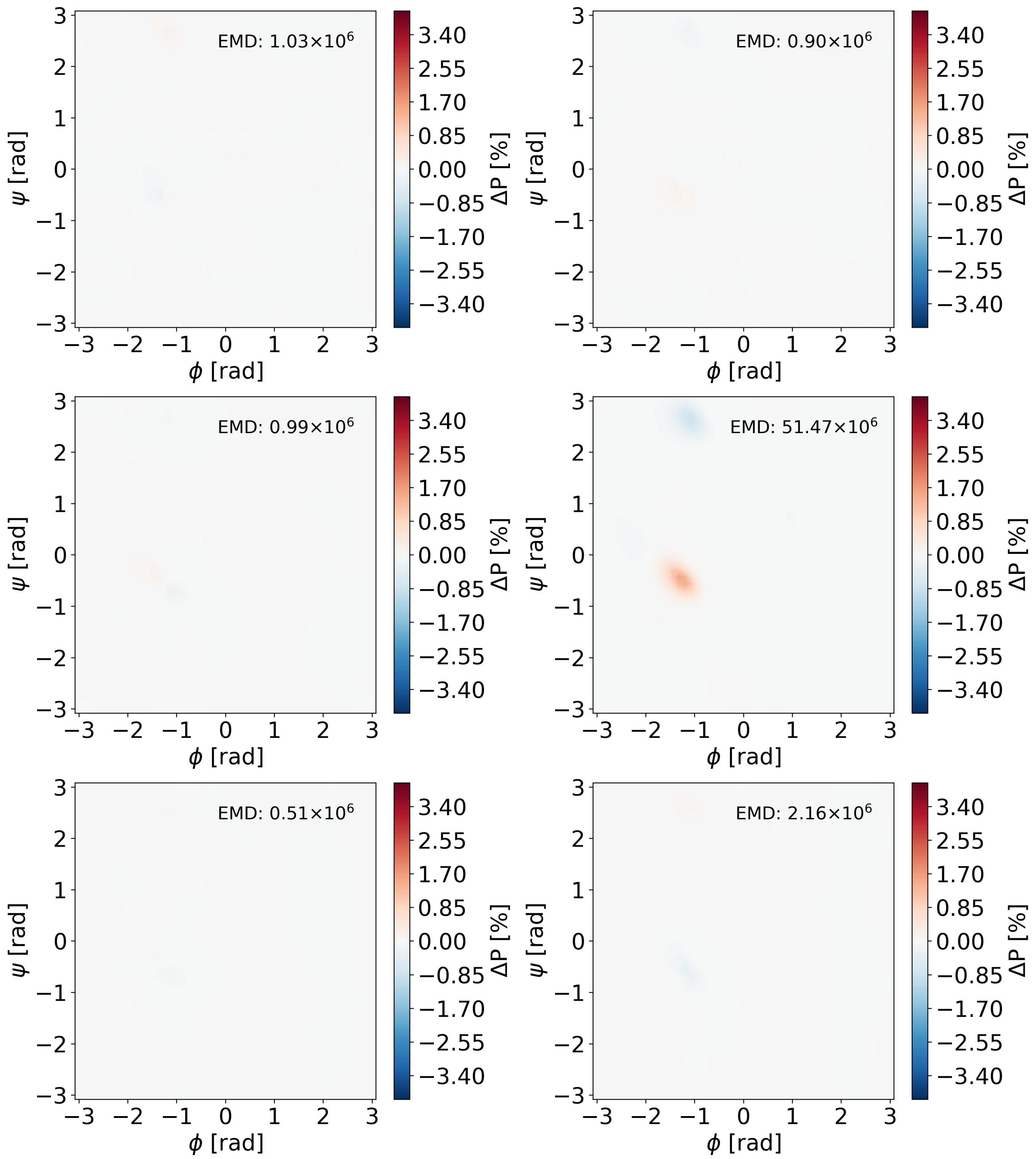

Figure S26: Difference in probability distribution and earth mover's distance (EMD) between two simulations. Difference between two simulations (200 ns overall sampling time each) using the AMP Hamiltonian trained according to entry 1 of Table 1 (top, left). Remaining entries (top to bottom, left to right): Difference between two simulations (600 ns overall sampling time each) using the AMP Hamiltonian trained according to entries 2, 8, 9, 10, and 11 to the one computed with the AMP Hamiltonian trained according to entry 1 of the same table. EMD is a similarity measure between two probability distributions as a solution of the optimal transport problem.

S3.3 Alanine Dipeptide – GFN2-xTB

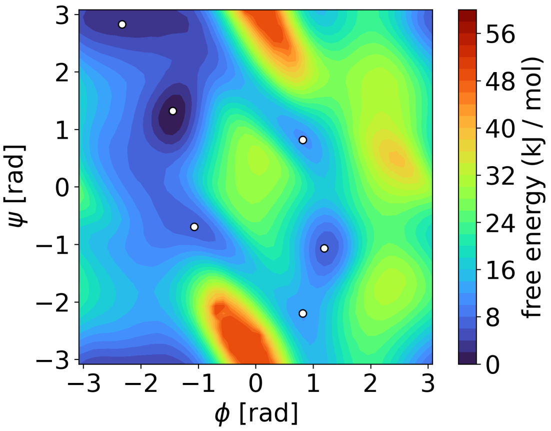

Figure S27: Free-energy landscape and local minima (white dots) calculated via umbrella sampling (600 ns overall sampling time) for alanine dipeptide **1** using the GFN2-xTB Hamiltonian.

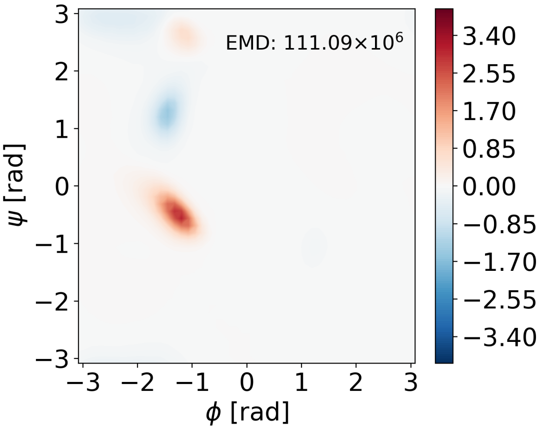

Figure S28: Difference in probability distribution and earth movers distance (EMD) between two simulations (600 ns overall sampling time each) using the AMP Hamiltonian trained according to entry 1 in Table 1 in the main text (600k parameter model) or using the GFN2-xTB Hamiltonian.

S3.4 Nickel Phosphine Complexes

Table S75: Comparison of free energies computed with static DFT calculations on the ωB97M-D4/def2-TZVPP, B3LYP-D3/def2-TZVP, and M06/def2-TZVP level of theories with geometries optimized with B3LYP-D3/6-31G(d,p)[SDD]. All values reported in kJ mol^{-1}. Experimental ligation state from *Science* **2021**, *374*, 301–308.

ligand	ligation state (exp.)	ωB97M-D4	B3LYP-D3	M06
PtBu3	1	-52.4	-45.1	-59.8
BrettPhos	1	-40.2	-32.7	-46.5
MeJohnPhos	2	42.7	61.7	32.7
CyJohnPhos	1	51.6	70.2	39.5
CataCXiumA	1	60.2	75.3	45.8
PCy3	2	78.6	92.3	71.6
CyTriceraPhos	2	80.5	111	80.6
CyTyrannoPhos	2	97.6	120	104
DrewPhos	2	124	147	126
PteroPhos	2	nan	nan	nan

Table S76: Comparison of free energies computed with AMP (600k parameter model) trained according to entry 1 in Table 3. All values reported in kJ mol^{-1}. Experimental ligation state from *Science* **2021**, *374*, 301–308.

ligand	ligation state (exp.)	$\Delta G^{\circ}_{diss,theo}$	error (theo.)
PtBu3	1	-41.8	nan
BrettPhos	1	-13.7	0.05
CyJohnPhos	1	-6.41	0.05
CataCXiumA	1	14.3	0.09
CyTriceraPhos	2	39.0	0.1
MeJohnPhos	2	40.3	0.1
PteroPhos	2	44.7	0.1
PCy3	2	48.3	0.3
CyTyrannoPhos	2	53.2	0.1
DrewPhos	2	70.1	0.1

Table S77: Comparison of free energies computed with AMP (2.7 parameter model) trained according to entry 1 in Table 3. All values reported in kJ mol^{-1}. Experimental ligation state from *Science* **2021**, *374*, 301–308.

ligand	ligation state (exp.)	$\Delta G^{\circ}_{diss,theo}$	error (theo.)
PtBu3	1	-41.8	nan
BrettPhos	1	-19.4	0.2
CataCXiumA	1	3.83	0.1
CyJohnPhos	1	7.60	0.2
PCy3	2	38.9	0.3
CyTriceraPhos	2	40.0	0.2
CyTyrannoPhos	2	44.3	0.2
PteroPhos	2	56.8	0.3
MeJohnPhos	2	62.1	0.2
DrewPhos	2	71.0	0.2

Table S78: Comparison of free energies computed with AMP (600k parameter model) trained according to entry 7 in Table 3. All values reported in kJ mol^{-1}. Experimental ligation state from *Science* **2021**, *374*, 301–308.

ligand	ligation state (exp.)	$\Delta G^{\circ}_{diss,theo}$	error (theo.)
PtBu3	1	-41.8	nan
BrettPhos	1	-26.5	0.1
CyJohnPhos	1	3.09	0.06
CataCXiumA	1	9.05	0.09
CyTriceraPhos	2	33.7	0.1
MeJohnPhos	2	34.9	0.1
PteroPhos	2	36.2	0.1
CyTyrannoPhos	2	47.8	0.1
PCy3	2	49.7	0.1
DrewPhos	2	53.7	0.1

Table S79: Comparison of free energies computed with AMP (2.7 parameter model) trained according to entry 7 in Table 3. All values reported in kJ mol^{-1}. Experimental ligation state from *Science* **2021**, *374*, 301–308.

ligand	ligation state (exp.)	$\Delta G^{\circ}_{diss,theo}$	error (theo.)
PtBu3	1	-41.8	nan
BrettPhos	1	-19.6	0.1
CyJohnPhos	1	7.62	0.2
CataCXiumA	1	10.2	0.2
PCy3	2	38.9	0.2
CyTriceraPhos	2	40.2	0.2
CyTyrannoPhos	2	42.4	0.2
PteroPhos	2	42.8	0.4
MeJohnPhos	2	64.5	0.2
DrewPhos	2	69.1	0.2

S3.5 Pyridine and Quinoline Dimers

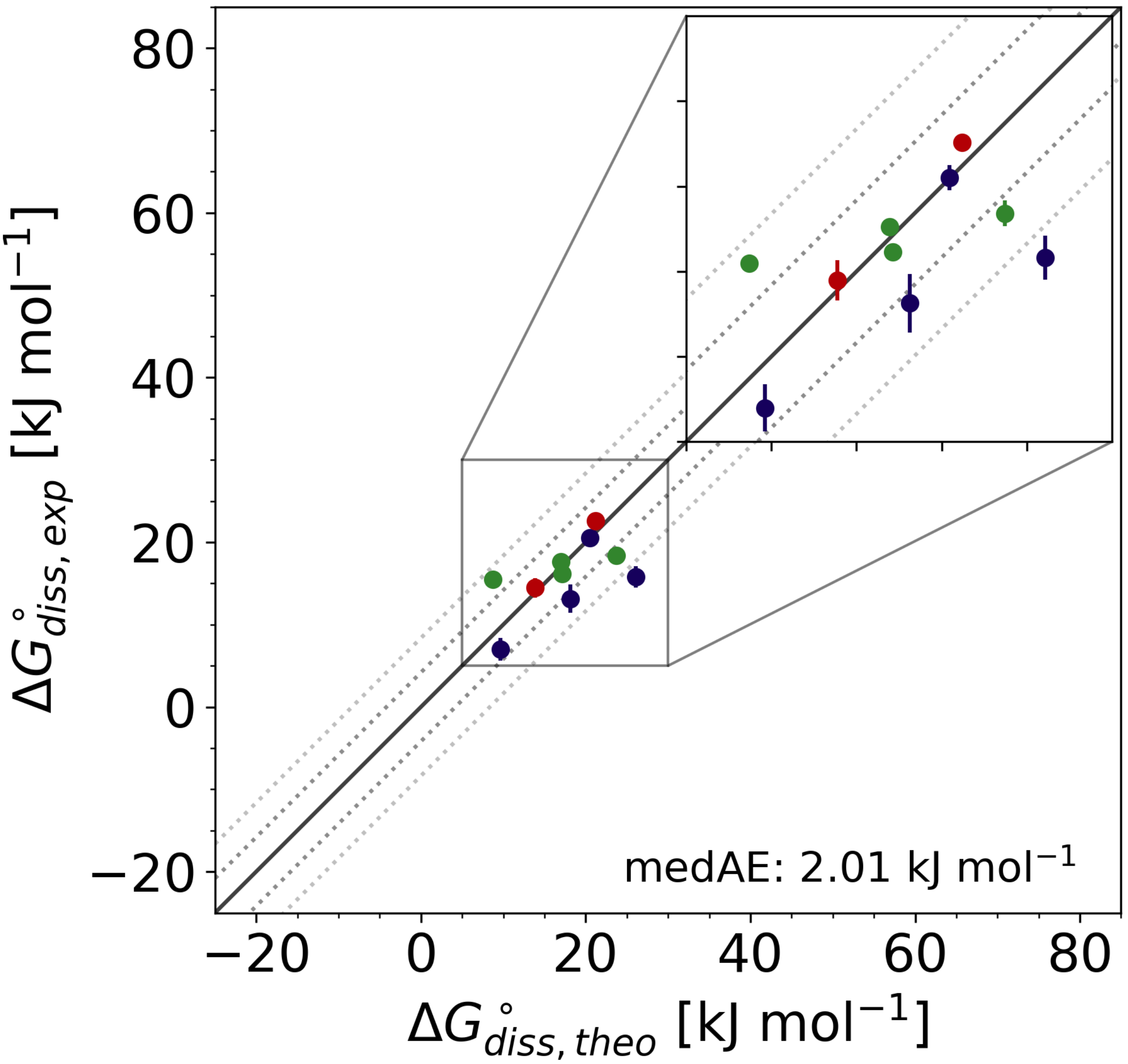

Figure S29: Correlation of experimental and theoretical dissociation free energies computed for a subset of dimers computed with AMP (600k parameter model) trained according to entry 1 in Table 4. The median absolute error (medAE) is shown. Windows of 4.184 and 8.368 kJ mol^{-1} are marked with dotted lines. Experimental values taken from *J. Am. Chem. Soc.* **2017**, *139*, 13126–13140.

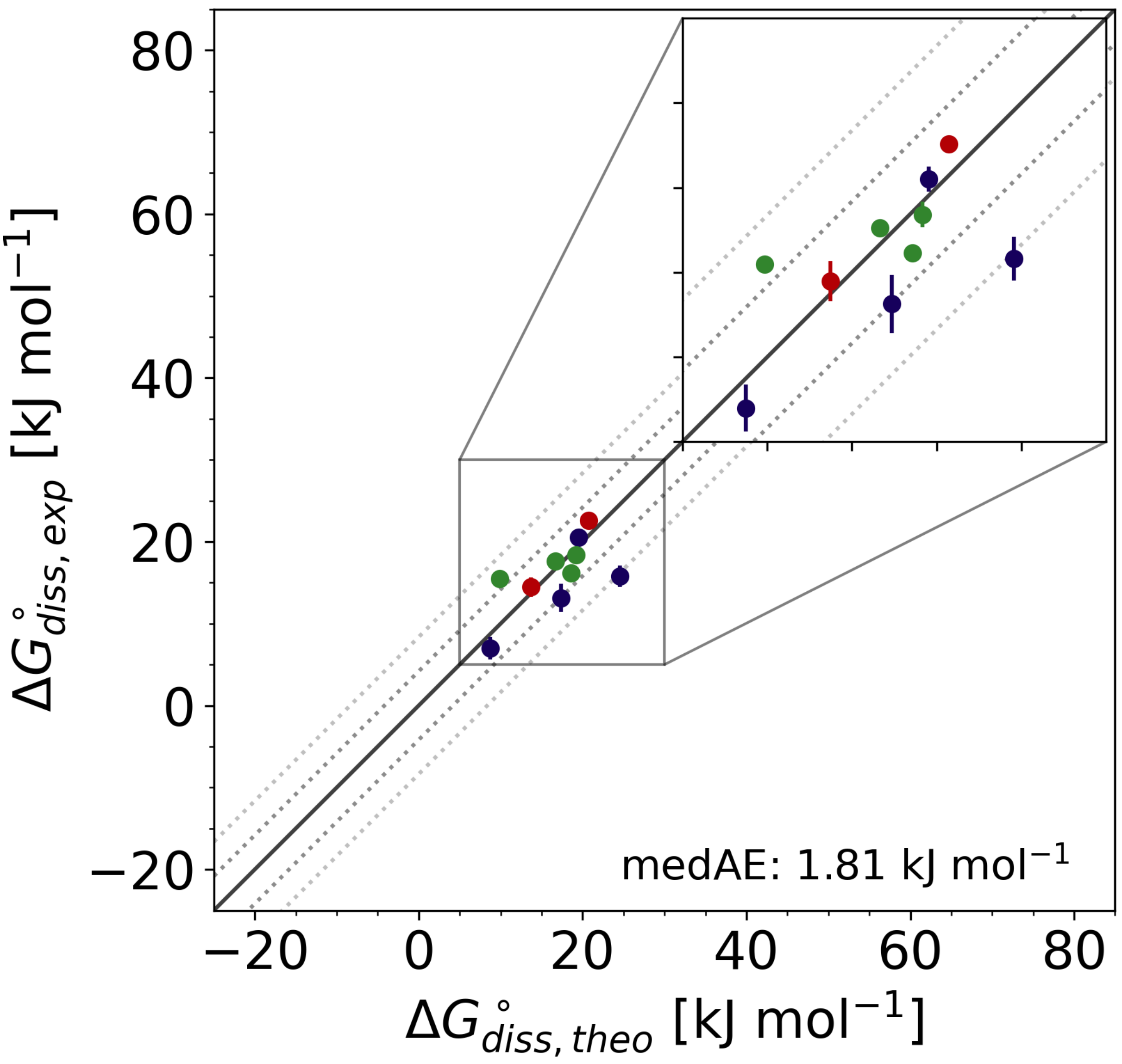

Figure S30: Correlation of experimental and theoretical dissociation free energies computed for a subset of dimers computed with AMP (2.7M parameter model) trained according to entry 1 in Table 4. The median absolute error (medAE) is shown. Windows of 4.184 and 8.368 kJ mol^{-1} are marked with dotted lines. Experimental values taken from *J. Am. Chem. Soc.* **2017**, *139*, 13126–13140.

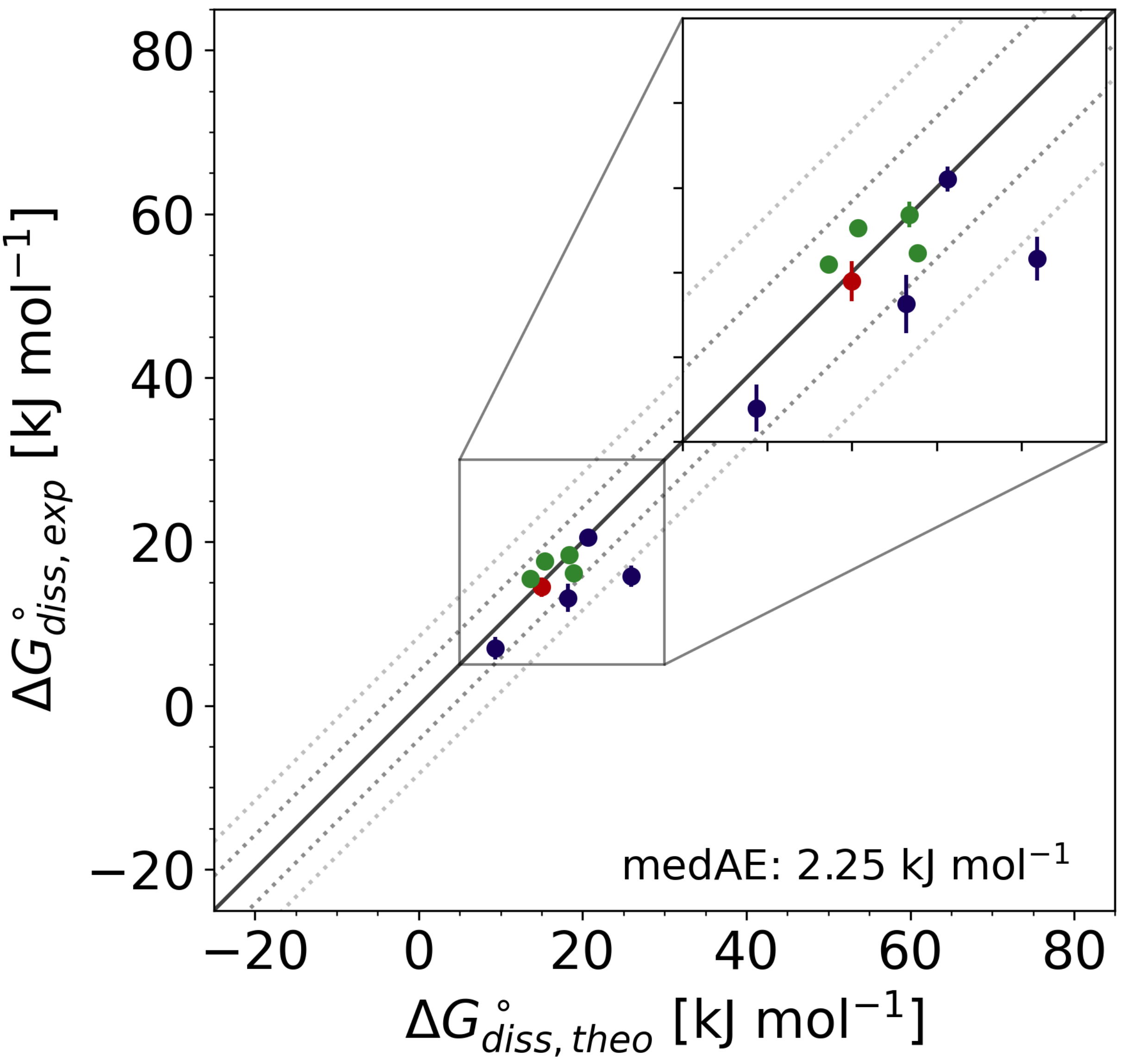

Figure S31: Correlation of experimental and theoretical dissociation free energies computed for a subset of dimers computed with AMP (600k parameter model) trained according to entry 7 in Table 4. The median absolute error (medAE) is shown. Windows of 4.184 and 8.368 kJ mol^{-1} are marked with dotted lines. Experimental values taken from *J. Am. Chem. Soc.* **2017**, *139*, 13126–13140.

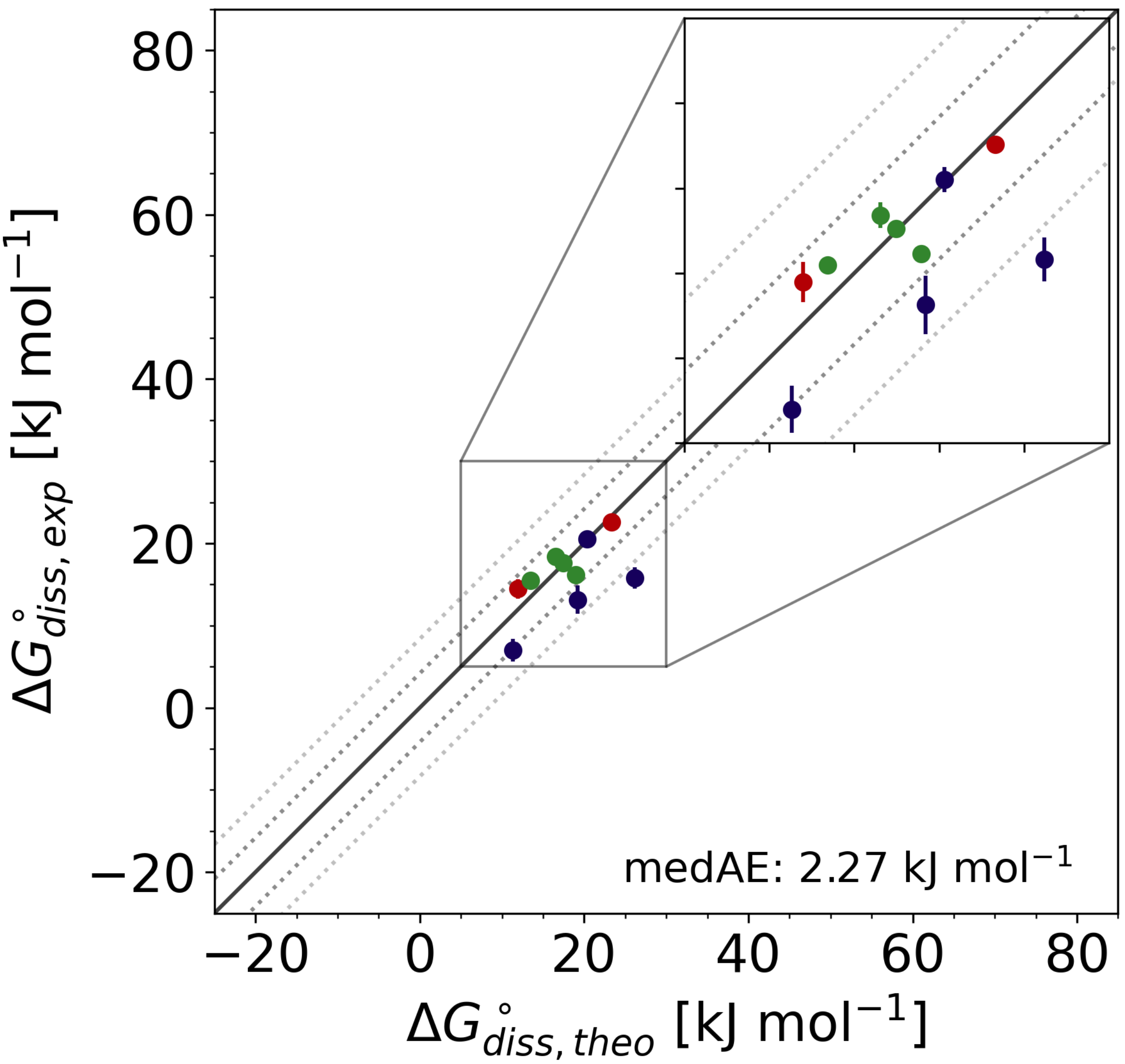

Figure S32: Correlation of experimental and theoretical dissociation free energies computed for a subset of dimers with computed with AMP (2.7M parameter model) trained according to entry 7 in Table 4. The median absolute error (medAE) is shown. Windows of 4.184 and 8.368 kJ mol^{-1} are marked with dotted lines. Experimental values taken from *J. Am. Chem. Soc.* **2017**, *139*, 13126–13140.

Table S80: Comparison of free energies computed with AMP (600k parameter model) trained according to entry 1 in Table 4. Median absolute error: 2.01 kJ mol^{-1}. All values reported in kJ mol^{-1}. Experimental values taken from *J. Am. Chem. Soc.* **2017**, *139*, 13126–13140.

Compound	$\Delta G^{\circ}_{diss,theo}$	$\Delta G^{\circ}_{diss,exp}$	$\Delta\Delta G^{\circ}_{diss}$	error (exp.)	error (theo.)
5a	20.5	20.5	0.0300	0.75	0.03
5b	26.1	15.8	10.3	1.3	0.008
5c	18.1	13.1	4.97	1.7	0.06
5e	9.62	6.99	2.63	1.4	0.04
6l	13.9	14.5	0.610	1.2	0.08
6m	21.2	22.6	1.39	0.46	0.08
7a	17.1	16.2	0.981	0.42	0.07
7e	8.70	15.5	6.78	0.084	0.02
7f	23.7	18.4	5.31	0.75	0.04
7g	17.0	17.6	0.655	0.50	0.07

Table S81: Comparison of free energies computed with AMP (2.7M parameter model) trained according to entry 1 in Table 4. Median absolute error: 1.81 kJ mol^{-1}. All values reported in kJ mol^{-1}. Experimental values taken from *J. Am. Chem. Soc.* **2017**, *139*, 13126–13140.

Compound	$\Delta G^{\circ}_{diss,theo}$	$\Delta G^{\circ}_{diss,exp}$	$\Delta\Delta G^{\circ}_{diss}$	error (exp.)	error (theo.)
5a	19.6	20.5	0.951	0.75	0.05
5b	24.5	15.8	8.73	1.3	0.008
5c	17.4	13.1	4.22	1.7	0.07
5e	8.75	6.99	1.76	1.4	0.03
6l	13.7	14.5	0.764	1.2	0.07
6m	20.7	22.6	1.87	0.46	0.08
7a	18.6	16.2	2.43	0.42	0.07
7e	9.86	15.5	5.63	0.084	0.03
7f	19.2	18.4	0.762	0.75	0.08
7g	16.7	17.6	0.953	0.50	0.07

Table S82: Comparison of free energies computed with AMP (600k parameter model) trained according to entry 7 in Table 4. Median absolute error: 2.25 kJ mol^{-1}. All values reported in kJ mol^{-1}. Experimental values taken from *J. Am. Chem. Soc.* **2017**, *139*, 13126–13140. Note that system **6m** is missing due to unstable simulations during prospective runs.

Compound	$\Delta G^{\circ}_{diss,theo}$	$\Delta G^{\circ}_{diss,exp}$	$\Delta\Delta G^{\circ}_{diss}$	error (exp.)	error (theo.)
5a	20.6	20.5	0.142	0.75	0.03
5b	25.9	15.8	10.1	1.3	0.008
5c	18.2	13.1	5.05	1.7	0.07
5e	9.35	6.99	2.36	1.4	0.04
6l	15.0	14.5	0.519	1.2	0.08
7a	18.9	16.2	2.73	0.42	0.06
7e	13.6	15.5	1.87	0.084	0.07
7f	18.4	18.4	0.0338	0.75	0.07
7g	15.4	17.6	2.25	0.50	0.07

Table S83: Comparison of free energies computed with AMP (2.7M parameter model) trained according to entry 7 in Table 4. Median absolute error: 2.27 kJ mol^{-1}. All values reported in kJ mol^{-1}. Experimental values taken from *J. Am. Chem. Soc.* **2017**, *139*, 13126–13140.

Compound	$\Delta G^{\circ}_{diss,theo}$	$\Delta G^{\circ}_{diss,exp}$	$\Delta\Delta G^{\circ}_{diss}$	error (exp.)	error (theo.)
5a	20.3	20.5	0.175	0.75	0.03
5b	26.2	15.8	10.4	1.3	0.008
5c	19.2	13.1	6.07	1.7	0.06
5e	11.3	6.99	4.33	1.4	0.06
6l	12.0	14.5	2.49	1.2	0.06
6m	23.3	22.6	0.706	0.46	0.07
7a	18.9	16.2	2.79	0.42	0.06
7e	13.4	15.5	2.04	0.084	0.07
7f	16.5	18.4	1.86	0.75	0.07
7g	17.5	17.6	0.135	0.50	0.06
